# Mental Illness Diagnoses May Not Cause All Mental Symptoms: A Simulation Study for Major Depressive Episodes, Dysthymic Disorder, and Manic Episodes

**DOI:** 10.7759/cureus.52234

**Published:** 2024-01-13

**Authors:** Yi-Sheng Chao, Chao-Jung Wu, June Y Po, Shih-Yu Huang, Hsing-Chien Wu, Hui-Ting Hsu, Yen-Po Cheng, Yi-Chun Lai, Wei-Chih Chen

**Affiliations:** 1 Epidemiology and Public Health, University of Montreal Hospital Centre Research Center, Montreal, CAN; 2 Computer Sciences, Université du Québec à Montréal, Montreal, CAN; 3 Epidemiology and Public Health, Natural Resources Institute, University of Greenwich, London, GBR; 4 Anesthesiology, Shuang Ho Hospital, New Taipei, TWN; 5 Internal Medicine, National Taiwan University Hospital, New Taipei, TWN; 6 Pathology, Changhua Christian Hospital, Changhua, TWN; 7 Neurological Surgery, Changhua Christian Hospital, Changhua, TWN; 8 Chest Medicine, National Yang Ming Chiao Tung University Hospital, Yilan, TWN; 9 Chest Medicine, Taipei Veterans General Hospital, Taipei, TWN

**Keywords:** diagnostic and statistical manual of mental disorders fourth edition text revision (dsm-iv-tr), correlations, odds ratios, causation, symptom mining, simulations

## Abstract

Objectives

This study aims to understand the statistical significance of the associations between diagnoses and symptoms based on simulations that have been used to understand the interpretability of mental illness diagnoses.

Methods

The symptoms for the diagnosis of major depressive episodes, dysthymic disorder, and manic episodes were extracted from the Diagnostic and Statistical Manual of Mental Disorders, Fourth Edition, Text Revision (DSM-IV-TR, American Psychiatric Association, Philadelphia, Pennsylvania). Without real-world symptom data, we simulated populations using various combinations of symptom prevalence and correlations. Assuming symptoms occurred with similar prevalence and correlations, for each combination of symptom prevalence (0.05, 0.1, 0.3, 0.5, and 0.7) and correlation (0, 0.1, 0.4, 0.7, and 0.9), 100 cohorts with 10,000 individuals were randomly created. Diagnoses were made according to the DSM-IV-TR criteria. The associations between the diagnoses and their input symptoms were quantified with odds ratios and correlation coefficients. P-values from 100 cohorts for each combination of symptom prevalence and correlation were summarized.

Results

Three mental illness diagnoses were not significantly correlated with their own symptoms in all simulations, particularly when symptoms were not correlated, except for the symptom in the major criteria of major depressive episodes or dysthymic disorder. The symptoms for the diagnosis of major depressive episodes and dysthymic disorder were significantly correlated with these two diagnoses in some simulations, assuming 0.1, 0.4, 0.7, or 0.9 symptom correlations, except for one symptom. The overlap in the input symptoms for the diagnosis of major depressive episodes and dysthymic disorder also leads to significant correlations between these two diagnoses, assuming 0.1, 0.4, 0.7, and 0.9 correlations between input symptoms. Manic episodes are not significantly associated with the input symptoms of major depressive episodes and dysthymic disorder.

Conclusion

There are challenges to establish the causation between psychiatric symptoms and mental illness diagnoses. There is insufficient prevalence and incidence data to show all psychiatric symptoms exist or can be observed in patients. The diagnostic accuracy of symptoms to detect a disease cause is far from perfect. Assuming the symptoms of three mood disorders may present in patients, three diagnoses are not significantly associated with all psychiatric symptoms used to diagnose them. The diagnostic criteria of the three diagnoses have not been designed to guarantee significant associations between symptoms and diagnoses. Because statistical associations are important for making causal inferences, there may be a lack of causation between diagnoses and symptoms. Previous research has identified factors that lead to insignificant associations between diagnoses and symptoms, including biases due to data processing and a lack of epidemiological evidence to support the design of mental illness diagnostic criteria.

## Introduction

Mental illnesses are associated with large disease burdens and adverse health outcomes, including mortality [1] and hypertension [2]. It is important to diagnose mental illnesses timely and treat them accordingly. Ideally, medical diagnoses represent underlying pathological changes or health conditions. The symptoms that are caused by these conditions and occur more frequently can be used to diagnose the underlying conditions [3]. Based on this principle, psychiatric symptoms have been identified by professionals to diagnose mental illnesses [4]. Mental illnesses are often diagnosed with complex criteria that consist of multiple psychiatric symptoms. The mental illnesses with the largest disease burden include major depressive disorder, dysthymic disorder, and bipolar disorder [5]. The diagnostic criteria of the above-mentioned three mental illnesses use different methods to aggregate information on the input symptoms [5]. For example, major depressive disorder can be confirmed in individuals with major depressive episodes according to the Diagnostic and Statistical Manual of Mental Disorders, Fourth Edition, Text Revision (DSM-IV-TR, American Psychiatric Association, Philadelphia, PA) [3,5]. To confirm each major depressive episode, individuals need to present five out of the nine symptoms, including depressed mood, feelings of worthlessness, and fatigue [5].

However, researchers have neglected how input symptoms are summarized and how they are used to diagnose health conditions can influence the relationships between these input symptoms and the diagnoses. For example, pairs of symptoms, such as sleeping too much and insomnia, are used to confirm a criterion in the minor criteria for the diagnosis of major depressive episodes [5]. When a patient has both symptoms and this individual is regarded the same as those with any one of the two symptoms, criteria of this kind ignore some information from input symptoms and induce biases to the diagnosis [5,6]. Several other methods to aggregate information from multiple input variables also introduce biases to the designated composite measures because of data censoring or inappropriate data transformation [5,6]. It has been recognized that biases of this sort have been introduced to the diagnostic criteria of major depressive episodes, dysthymic disorder, and manic episodes [5]. These biases lead to mental illness diagnoses that could not be well explained by their input symptoms [5]. In a previous study, assuming similar symptom prevalence, one of the consequences of these biases is that the diagnoses of the three mental illnesses cannot be fully explained by their own input variables, except for one situation, in which all of the symptoms of dysthymic disorder are prevalent among at least 70% of the population [5].

Despite the existence of the biases introduced by data processing, the role of these biases in the validity of mental illnesses and how they may influence the causal relationships between diagnoses and symptoms are not concluded. There are several approaches to establishing causal relationships [7], for example, the Bradford-Hill Criteria [8]. For this method and other causal inference methods, it is important to examine the strength of associations between the cause and its consequences [8]. To our knowledge, we did not find research that specifies the strengths of associations between psychiatric symptoms and mental illness diagnoses [5]. Confirming significant associations between them could be the first step to establish the causal relationships between them. This study aims to examine the statistical significance of the associations between the diagnoses of three mental illnesses and their input symptoms.

## Materials and methods

Three of the mental illnesses with the largest disease burden, major depressive disorder, dysthymic disorder, and bipolar disorder, were chosen for analysis [5]. The diagnosis of major depressive episodes (episodes required for the diagnosis of major depressive disorder), dysthymic disorder, and manic episodes (required for the diagnosis of bipolar disorder) was based on the DSM-IV-TR [4,9]. Populations with various symptom prevalence rates and symptom correlations were simulated [5] to understand the significance of associations between the diagnoses and their input symptoms. This simulation model and its assumptions were published elsewhere [5] and explained in detail in the next paragraph. The R codes to simulate the populations are available online [5].

In detail, the presence of major depressive episodes was important to confirm the diagnosis of major depressive disorder [4]. Manic episodes were used to confirm the diagnosis of bipolar disorder [4]. There were 15, 11, and 13 input symptoms extracted from the diagnosis of major depressive episodes, dysthymic disorder, and manic episodes, respectively, based on their DSM-IV-TR diagnostic criteria (see Table 1 for details) [5]. In the simulations, individuals were randomly assigned with the input symptoms based on the assumed prevalence rates and symptom correlations [5]. Due to random assignment, there were some variations in the prevalence rates and between-variable correlations of all input symptoms, but they remained similar to the assumed values [5]. The prevalence rates were assumed to be 0.05, 0.1, 0.3, 0.5, and 0.7 for all symptoms [5]. The correlations between the input symptoms were assumed to be 0, 0.1, 0.4, 0.7, and 0.9 for all symptoms [5]. There were 25 combinations of the assumed prevalence rates and between-variable correlations [5]. In this study, 100 cohorts with 10,000 individuals were randomly created for each combination [5]. Based on the presence of the input symptoms, the individuals qualified for the diagnoses were labeled according to the diagnostic criteria [5].

**Table 1:** Variables and mental symptoms for the diagnosis of major depressive episodes, dysthymic disorder, and manic episodes *input symptoms for both major depressive episodes and dysthymic disorder. Note: symptoms identified from the diagnostic criteria in the Diagnostic and Statistical Manual of Mental Disorders, Fourth Edition, Text Revision. Detailed descriptions about the variables are available online [5].

Variables	Major or minor criteria, diagnoses, or bias variables	Symptoms and definitions
mde	Diagnosis	Major Depressive Episodes for the diagnosis of Major Depressive Disorder
mde_ma1	Major	Depressed mood for more than two weeks.
mde_ma2	Major	Loss of interest or pleasure in daily activities for more than two weeks.
mde_mi3	Minor	Significant unintentional weight loss or gain
mde_mi3_1	Minor	Significant unintentional weight gain
mde_mi3_2	Minor	Significant unintentional weight loss
mde_mi3_bias	Bias	Information of the domain not explained by the input variables
mde_mi4*	Minor	Insomnia or sleeping too much
mde_mi4_1*	Minor	Insomnia
mde_mi4_2*	Minor	Sleeping too much
mde_mi4_bias*	Bias	Information of the domain not explained by the input variables
mde_mi5	Minor	Agitation or psychomotor retardation noticed by others
mde_mi5_1	Minor	Agitation
mde_mi5_2	Minor	Psychomotor retardation noticed by others
mde_mi5_bias	Bias	Information of the domain not explained by the input variables
mde_mi6*	Minor	Fatigue or loss of energy
mde_mi6_1*	Minor	Fatigue
mde_mi6_2*	Minor	Loss of energy
mde_mi6_bias*	Bias	Information of the domain not explained by the input variables
mde_mi7	Minor	Feelings of worthlessness or excessive guilt
mde_mi7_1	Minor	Feelings of worthlessness
mde_mi7_2	Minor	Feelings of excessive guilt
mde_mi7_bias	Bias	Information of the domain not explained by the input variables
mde_mi8*	Minor	Diminished ability to think or concentrate, or indecisiveness+
mde_mi8_1*	Minor	Diminished ability to think or concentrate
mde_mi8_2*	Minor	Indecisiveness
mde_mi8_bias*	Bias	Information of the domain not explained by the input variables
mde_mi9	Minor	Recurrent thoughts of death
mde_bias1	Minor	Information due to top censoring by choosing three domains in minor criteria
mde_bias2	Minor	Information due to top censoring by choosing four domains in minor criteria
mde_bias	Bias	Information of diagnosis not explained by the domains
dys	Diagnosis	Dysthymic Disorder
dys_ma	Major	Depressed mood most of the day for more days than not, for at least 2 years
dys_mi	Minor	Minor criteria (at least 2 items)
dys_mi1	Minor	Poor appetite or overeating
dys_mi1_1	Minor	Poor appetite
dys_mi1_2	Minor	Overeating
dys_mi1_bias	Bias	Information of the domain not explained by the input variables
dys_mi4	Minor	Low self-esteem
dys_mi6	Minor	Feelings of hopelessness
dys_mi_bias	Bias	Information of minor criteria not explained by input variables
dys_bias	Bias	Information of diagnosis not explained by major or minor criteria
manic	Diagnosis	Manic episodes for the diagnosis of bipolar disorder
man_ma1	Major	Elevated mood, lasting at least 1 week
man_ma2	Major	Expansive mood, lasting at least 1 week
man_ma3	Major	Irritable mood, lasting at least 1 week
man_mi1	Minor	Increased self-esteem or grandiosity
man_mi1_1	Minor	Increased self-esteem
man_mi1_2	Minor	Grandiosity
man_mi1_bias	Bias	Information of the domain not explained by the input variables
man_mi2	Minor	Decreased need for sleep (e.g., feels rested after only 3 hours of sleep)
man_mi3	Minor	More talkative than usual or pressure to keep talking
man_mi3_1	Minor	More talkative than usual
man_mi3_2	Minor	Pressure to keep talking
man_mi3_bias	Bias	Information of the domain not explained by the input variables
man_mi4	Minor	Flight of ideas or subjective experience that thoughts are racing
man_mi4_1	Minor	Flight of ideas
man_mi4_2	Minor	Subjective experience that thoughts are racing
man_mi4_bias	Bias	Information of the domain not explained by the input variables
man_mi5	Minor	Distractibility (i.e., attention too easily drawn to unimportant or irrelevant external stimuli)
man_mi6	Minor	Increase in goal-directed activity (either socially, at work or school, or sexually) or psychomotor agitation
man_mi6_1	Minor	Increase in goal-directed activity
man_mi6_2	Minor	Psychomotor agitation
man_mi6_bias	Bias	Information of the domain not explained by the input variables
man_mi7	Minor	Excessive involvement in pleasurable activities that have a high potential for painful consequences (e.g., engaging in unrestrained buying sprees, sexual indiscretions, or foolish business investments)"
man_bias1	Bias	Information of diagnosis due to top-censoring for choosing at least three symptoms
man_bias2	Bias	Information of diagnosis due to top-censoring for choosing at least four symptoms
man_bias	Bias	Information of diagnosis not explained by symptoms

Measures of association

The associations between the diagnoses and their input symptoms were quantified with odds ratios and correlation coefficients [10]. Specifically, the correlation between each symptom and each diagnosis was determined in a simulated population. The odds ratio of developing each symptom due to a diagnosis was also quantified in a simulated population. Correlations were negligible, weak, moderate, strong, or very strong, when the absolute values of the coefficients were between 0 and 0.10, 0.10 and 0.39, 0.40 and 0.69, 0.70 and 0.89, and 0.90 and 1, respectively [11]. The odds ratios were calculated by dividing the odds of developing symptoms among those diagnosed with the odds of developing symptoms among those not diagnosed [10,12,13]. The null hypothesis was that the odds of developing symptoms among those diagnosed were the same as that among those not diagnosed (odds ratios = 1). Statistical significance, p-values, was derived accordingly [14]. P-values, two-tailed, less than 0.05 were considered statistical significance [15,16]. All simulations and statistical analyses were conducted under R environment (v 4.0.4) [17] and RStudio (v 1.4.1103) [18].

## Results

The proportions of the populations diagnosed with major depressive episodes, dysthymic disorder, and manic episodes are summarized by assumed symptom prevalence and correlations in Table 2 [5]. In general, the prevalence of the three diagnoses is less than that of the input symptoms under all combinations of the prevalence and between-variable correlations of the input symptoms due to the design of the diagnostic criteria [5].

**Table 2:** Observed prevalence of major depressive episodes, dysthymic disorder, and manic episodes based on the assumed symptom prevalence and correlations Mean (95% CI) Median (range) in the cells. *normality assumed, since the p values of Shapiro-Wilk’s test > 0.05. Summary statistics obtained from 100 simulations, each of which included 10,000 simulated subjects. Note: individuals were randomly assigned according to the assumed prevalence and correlations. The observed symptom prevalence and correlations were similar to the assumed values. The simulations can be reproduced using R codes available online [5].

Correlations between input symptoms	Assumed prevalence of input symptoms	Major depressive episodes	Dysthymic disorder	Manic episodes
0	0.05	0 (95% CI = 0 to 0)	0.004 (95% CI = 0.004 to 0.004)	0 (95% CI = 0 to 0)
		0 (range = 0 to 0)	0.004 (range = 0.003 to 0.006)*	0 (range = 0 to 0)
0	0.1	0.001 (95% CI = 0.001 to 0.001)	0.024 (95% CI = 0.024 to 0.025)	0.002 (95% CI = 0.002 to 0.002)
		0.001 (range = 0 to 0.002)*	0.024 (range = 0.02 to 0.027)	0.002 (range = 0.001 to 0.004)*
0	0.3	0.067 (95% CI = 0.066 to 0.068)	0.249 (95% CI = 0.248 to 0.25)	0.136 (95% CI = 0.135 to 0.136)
		0.067 (range = 0.06 to 0.073)*	0.25 (range = 0.237 to 0.257)*	0.136 (range = 0.126 to 0.147)*
0	0.5	0.244 (95% CI = 0.244 to 0.245)	0.493 (95% CI = 0.492 to 0.494)	0.436 (95% CI = 0.435 to 0.437)
		0.244 (range = 0.235 to 0.257)*	0.493 (range = 0.483 to 0.506)*	0.435 (range = 0.426 to 0.45)*
0	0.7	0.49 (95% CI = 0.489 to 0.491)	0.7 (95% CI = 0.7 to 0.701)	0.691 (95% CI = 0.691 to 0.692)
		0.49 (range = 0.479 to 0.508)*	0.701 (range = 0.687 to 0.713)*	0.692 (range = 0.676 to 0.701)*
0.1	0.05	0.004 (95% CI = 0.003 to 0.004)	0.018 (95% CI = 0.018 to 0.018)	0.007 (95% CI = 0.007 to 0.007)
		0.004 (range = 0.002 to 0.006)*	0.018 (range = 0.015 to 0.021)*	0.007 (range = 0.005 to 0.01)*
0.1	0.1	0.011 (95% CI = 0.011 to 0.011)	0.049 (95% CI = 0.048 to 0.049)	0.022 (95% CI = 0.021 to 0.022)
		0.011 (range = 0.009 to 0.014)*	0.049 (range = 0.041 to 0.055)*	0.022 (range = 0.019 to 0.027)*
0.1	0.3	0.094 (95% CI = 0.093 to 0.095)	0.25 (95% CI = 0.249 to 0.25)	0.172 (95% CI = 0.171 to 0.172)
		0.094 (range = 0.087 to 0.1)*	0.25 (range = 0.242 to 0.258)*	0.171 (range = 0.162 to 0.18)*
0.1	0.5	0.268 (95% CI = 0.267 to 0.269)	0.482 (95% CI = 0.481 to 0.483)	0.426 (95% CI = 0.425 to 0.427)
		0.268 (range = 0.257 to 0.278)*	0.482 (range = 0.471 to 0.495)*	0.426 (range = 0.414 to 0.436)*
0.1	0.7	0.51 (95% CI = 0.509 to 0.51)	0.696 (95% CI = 0.696 to 0.697)	0.679 (95% CI = 0.678 to 0.68)
		0.509 (range = 0.499 to 0.52)*	0.696 (range = 0.687 to 0.705)*	0.679 (range = 0.662 to 0.69)*
0.4	0.05	0.019 (95% CI = 0.019 to 0.019)	0.037 (95% CI = 0.037 to 0.038)	0.028 (95% CI = 0.028 to 0.029)
		0.019 (range = 0.016 to 0.022)*	0.037 (range = 0.033 to 0.042)*	0.028 (range = 0.024 to 0.032)*
0.4	0.1	0.042 (95% CI = 0.042 to 0.042)	0.078 (95% CI = 0.077 to 0.079)	0.062 (95% CI = 0.062 to 0.063)
		0.042 (range = 0.036 to 0.047)*	0.078 (range = 0.071 to 0.085)*	0.062 (range = 0.057 to 0.066)*
0.4	0.3	0.167 (95% CI = 0.166 to 0.167)	0.267 (95% CI = 0.266 to 0.268)	0.231 (95% CI = 0.23 to 0.232)
		0.166 (range = 0.156 to 0.18)*	0.267 (range = 0.254 to 0.277)*	0.231 (range = 0.222 to 0.241)*
0.4	0.5	0.343 (95% CI = 0.342 to 0.344)	0.475 (95% CI = 0.474 to 0.476)	0.44 (95% CI = 0.439 to 0.441)
		0.343 (range = 0.331 to 0.354)*	0.475 (range = 0.465 to 0.484)*	0.44 (range = 0.428 to 0.451)*
0.4	0.7	0.571 (95% CI = 0.57 to 0.572)	0.689 (95% CI = 0.688 to 0.69)	0.666 (95% CI = 0.665 to 0.667)
		0.571 (range = 0.555 to 0.583)*	0.689 (range = 0.678 to 0.7)*	0.666 (range = 0.655 to 0.678)*
0.7	0.05	0.035 (95% CI = 0.035 to 0.035)	0.045 (95% CI = 0.045 to 0.046)	0.042 (95% CI = 0.041 to 0.042)
		0.035 (range = 0.031 to 0.039)*	0.046 (range = 0.039 to 0.051)*	0.042 (range = 0.035 to 0.046)*
0.7	0.1	0.071 (95% CI = 0.071 to 0.072)	0.092 (95% CI = 0.092 to 0.093)	0.085 (95% CI = 0.084 to 0.085)
		0.071 (range = 0.065 to 0.078)*	0.092 (range = 0.087 to 0.099)	0.085 (range = 0.078 to 0.092)*
0.7	0.3	0.234 (95% CI = 0.233 to 0.235)	0.286 (95% CI = 0.285 to 0.287)	0.27 (95% CI = 0.269 to 0.271)
		0.234 (range = 0.224 to 0.243)*	0.285 (range = 0.275 to 0.298)*	0.27 (range = 0.258 to 0.281)*
0.7	0.5	0.421 (95% CI = 0.42 to 0.422)	0.485 (95% CI = 0.485 to 0.486)	0.469 (95% CI = 0.468 to 0.47)
		0.421 (range = 0.402 to 0.434)*	0.486 (range = 0.472 to 0.497)*	0.468 (range = 0.455 to 0.481)*
0.7	0.7	0.635 (95% CI = 0.634 to 0.636)	0.69 (95% CI = 0.69 to 0.691)	0.678 (95% CI = 0.677 to 0.679)
		0.634 (range = 0.627 to 0.647)*	0.69 (range = 0.679 to 0.702)*	0.677 (range = 0.669 to 0.69)*
0.9	0.05	0.042 (95% CI = 0.041 to 0.042)	0.048 (95% CI = 0.047 to 0.048)	0.046 (95% CI = 0.046 to 0.046)
		0.042 (range = 0.037 to 0.046)*	0.048 (range = 0.042 to 0.054)*	0.046 (range = 0.041 to 0.05)*
0.9	0.1	0.085 (95% CI = 0.085 to 0.086)	0.097 (95% CI = 0.096 to 0.097)	0.093 (95% CI = 0.092 to 0.093)
		0.085 (range = 0.08 to 0.093)*	0.096 (range = 0.092 to 0.106)*	0.093 (range = 0.084 to 0.1)*
0.9	0.3	0.268 (95% CI = 0.268 to 0.269)	0.294 (95% CI = 0.293 to 0.294)	0.286 (95% CI = 0.285 to 0.287)
		0.269 (range = 0.26 to 0.281)*	0.293 (range = 0.283 to 0.306)*	0.286 (range = 0.272 to 0.295)*
0.9	0.5	0.463 (95% CI = 0.462 to 0.464)	0.492 (95% CI = 0.491 to 0.493)	0.485 (95% CI = 0.484 to 0.486)
		0.463 (range = 0.45 to 0.475)*	0.493 (range = 0.479 to 0.508)*	0.485 (range = 0.471 to 0.498)*
0.9	0.7	0.668 (95% CI = 0.667 to 0.669)	0.694 (95% CI = 0.693 to 0.695)	0.688 (95% CI = 0.687 to 0.689)
		0.668 (range = 0.653 to 0.681)*	0.695 (range = 0.678 to 0.705)*	0.688 (range = 0.678 to 0.7)*

Major depressive episodes and psychiatric symptoms

In Table 3, the statistical significance of the correlations or odds ratios between mental illness diagnoses and symptoms are listed. The median and mean values and standard deviations were derived from 100 simulations for each combination of symptom prevalence and correlations. The numbers of simulations with significant correlations or odds ratios were labelled. The p-values of correlations or odds ratios in the 100 simulations were all between 0.05 and 0.001 or less than 0.001 were separately labeled.

**Table 3:** Correlations between major depressive episodes and their input symptoms by assumed symptom prevalence and correlations Mean (standard deviation) *<number> = the numbers of significant statistics (p < 0.05) in 100 simulations !# = p < 0.001 in all of the 100 simulations !@ = p < 0.001 in some of the 100 simulations mde = Major Depressive Episodes for the diagnosis of Major Depressive Disorder; mde_ma1 = Depressed mood for more than two weeks.; mde_ma2 = Loss of interest or pleasure in daily activities for more than two weeks.; mde_mi3 = Significant unintentional weight loss or gain; mde_mi3_1 = Significant unintentional weight gain; mde_mi3_2 = Significant unintentional weight loss; mde_mi3_bias = Information of the domain not explained by the input variables; mde_mi4 = Insomnia or sleeping too much; mde_mi4_1 = Insomnia; mde_mi4_2 = Sleeping too much; mde_mi4_bias = Information of the domain not explained by the input variables; mde_mi5 = Agitation or psychomotor retardation noticed by others; mde_mi5_1 = Agitation; mde_mi5_2 = Psychomotor retardation noticed by others; mde_mi5_bias = Information of the domain not explained by the input variables; mde_mi6 = Fatigue or loss of energy; mde_mi6_1 = Fatigue; mde_mi6_2 = Loss of energy; mde_mi6_bias = Information of the domain not explained by the input variables; mde_mi7 = Feelings of worthlessness or excessive guilt; mde_mi7_1 = Feelings of worthlessness; mde_mi7_2 = Feelings of excessive guilt; mde_mi7_bias = Information of the domain not explained by the input variables; mde_mi8 = Diminished ability to think or concentrate, or indecisiveness+; mde_mi8_1 = Diminished ability to think or concentrate; mde_mi8_2 = Indecisiveness; mde_mi8_bias = Information of the domain not explained by the input variables; mde_mi9 = Recurrent thoughts of death; mde_bias1 = Information due to top censoring by choosing three domains in minor criteria; mde_bias2 = Information due to top censoring by choosing four domains in minor criteria; mde_bias = Information of diagnosis not explained by the domain

Assumed prevalence	0.05	0.1	0.3	0.5	0.7	0.05	0.1	0.3	0.5	0.7	0.05	0.1	0.3	0.5	0.7	0.05	0.1	0.3	0.5	0.7	0.05	0.1	0.3	0.5	0.7
Assumed correlations between symptoms	0	0	0	0	0	0.1	0.1	0.1	0.1	0.1	0.4	0.4	0.4	0.4	0.4	0.7	0.7	0.7	0.7	0.7	0.9	0.9	0.9	0.9	0.9
mde	1 (0) *<100>	1 (0) *<100>!#	1 (0) *<100>!#	1 (0) *<100>!#	1 (0) *<100>!#	1 (0) *<100>!#	1 (0) *<100>!#	1 (0) *<100>!#	1 (0) *<100>!#	1 (0) *<100>!#	1 (0) *<100>!#	1 (0) *<100>!#	1 (0) *<100>!#	1 (0) *<100>!#	1 (0) *<100>!#	1 (0) *<100>!#	1 (0) *<100>!#	1 (0) *<100>!#	1 (0) *<100>!#	1 (0) *<100>!#	1 (0) *<100>!#	1 (0) *<100>!#	1 (0) *<100>!#	1 (0) *<100>!#	1 (0) *<100>!#
mde_ma1	0.05 (0.01) *<100>	0.1 (0.01) *<100>!#	0.41 (0.01) *<100>!#	0.57 (0.01) *<100>!#	0.64 (0.01) *<100>!#	0.26 (0.02) *<100>!#	0.31 (0.02) *<100>!#	0.49 (0.01) *<100>!#	0.6 (0.01) *<100>!#	0.67 (0.01) *<100>!#	0.61 (0.02) *<100>!#	0.63 (0.01) *<100>!#	0.68 (0.01) *<100>!#	0.72 (0.01) *<100>!#	0.75 (0.01) *<100>!#	0.83 (0.01) *<100>!#	0.83 (0.01) *<100>!#	0.84 (0.01) *<100>!#	0.85 (0) *<100>!#	0.86 (0) *<100>!#	0.91 (0.01) *<100>!#	0.92 (0.01) *<100>!#	0.93 (0) *<100>!#	0.93 (0) *<100>!#	0.93 (0) *<100>!#
mde_ma2	0.05 (0.01) *<100>	0.1 (0.01) *<100>!#	0.41 (0.01) *<100>!#	0.57 (0.01) *<100>!#	0.64 (0.01) *<100>!#	0.26 (0.02) *<100>!#	0.32 (0.02) *<100>!#	0.49 (0.01) *<100>!#	0.6 (0.01) *<100>!#	0.67 (0.01) *<100>!#	0.61 (0.02) *<100>!#	0.63 (0.01) *<100>!#	0.68 (0.01) *<100>!#	0.72 (0.01) *<100>!#	0.75 (0.01) *<100>!#	0.83 (0.01) *<100>!#	0.83 (0.01) *<100>!#	0.84 (0.01) *<100>!#	0.85 (0) *<100>!#	0.86 (0) *<100>!#	0.91 (0.01) *<100>!#	0.91 (0.01) *<100>!#	0.92 (0) *<100>!#	0.93 (0) *<100>!#	0.93 (0) *<100>!#
mde_mi3	0.02 (0.02) *<52>	0.02 (0.01) *<63>	0.05 (0.01) *<100>	0.01 (0.01) *<28>	0 (0.01) *<4>	0.11 (0.02) *<100>!#	0.13 (0.01) *<100>!#	0.15 (0.01) *<100>!#	0.14 (0.01) *<100>!#	0.12 (0.01) *<100>!#	0.4 (0.02) *<100>!#	0.41 (0.01) *<100>!#	0.42 (0.01) *<100>!#	0.43 (0.01) *<100>!#	0.42 (0.01) *<100>!#	0.69 (0.01) *<100>!#	0.68 (0.01) *<100>!#	0.69 (0.01) *<100>!#	0.69 (0.01) *<100>!#	0.69 (0.01) *<100>!#	0.82 (0.01) *<100>!#	0.83 (0.01) *<100>!#	0.84 (0.01) *<100>!#	0.85 (0) *<100>!#	0.84 (0.01) *<100>!#
mde_mi3_1	0.01 (0.02) *<22>	0.02 (0.01) *<33>	0.03 (0.01) *<87>	0.01 (0.01) *<12>	0 (0.01) *<1>	0.09 (0.02) *<100>!#	0.1 (0.01) *<100>!#	0.12 (0.01) *<100>!#	0.13 (0.01) *<100>!#	0.13 (0.01) *<100>!#	0.4 (0.02) *<100>!#	0.41 (0.02) *<100>!#	0.42 (0.01) *<100>!#	0.43 (0.01) *<100>!#	0.44 (0.01) *<100>!#	0.71 (0.02) *<100>!#	0.7 (0.01) *<100>!#	0.71 (0.01) *<100>!#	0.71 (0.01) *<100>!#	0.72 (0.01) *<100>!#	0.84 (0.02) *<100>!#	0.85 (0.01) *<100>!#	0.86 (0.01) *<100>!#	0.86 (0.01) *<100>!#	0.86 (0.01) *<100>!#
mde_mi3_2	0.02 (0.02) *<40>	0.02 (0.01) *<44>	0.03 (0.01) *<84>	0.01 (0.01) *<12>	0 (0.01) *<6>	0.09 (0.02) *<100>!@	0.1 (0.02) *<100>!#	0.13 (0.01) *<100>!#	0.13 (0.01) *<100>!#	0.13 (0.01) *<100>!#	0.4 (0.02) *<100>!#	0.41 (0.02) *<100>!#	0.42 (0.01) *<100>!#	0.43 (0.01) *<100>!#	0.44 (0.01) *<100>!#	0.71 (0.02) *<100>!#	0.7 (0.01) *<100>!#	0.71 (0.01) *<100>!#	0.71 (0.01) *<100>!#	0.72 (0.01) *<100>!#	0.84 (0.01) *<100>!#	0.85 (0.01) *<100>!#	0.86 (0.01) *<100>!#	0.86 (0) *<100>!#	0.86 (0.01) *<100>!#
mde_mi3_bias	0 (0)	-0.01 (0.02) *<27>	-0.02 (0.01) *<38>	-0.01 (0.01) *<7>	0 (0.01) *<6>	-0.1 (0.04) *<99>	-0.1 (0.02) *<100>!#	-0.13 (0.01) *<100>!#	-0.14 (0.01) *<100>!#	-0.15 (0.01) *<100>!#	-0.46 (0.03) *<100>!#	-0.45 (0.02) *<100>!#	-0.46 (0.01) *<100>!#	-0.48 (0.01) *<100>!#	-0.49 (0.01) *<100>!#	-0.75 (0.02) *<100>!#	-0.75 (0.01) *<100>!#	-0.75 (0.01) *<100>!#	-0.75 (0.01) *<100>!#	-0.76 (0.01) *<100>!#	-0.87 (0.02) *<100>!#	-0.87 (0.01) *<100>!#	-0.88 (0.01) *<100>!#	-0.89 (0) *<100>!#	-0.89 (0.01) *<100>!#
mde_mi4	0.02 (0.02) *<55>	0.03 (0.01) *<66>	0.04 (0.01) *<99>	0.01 (0.01) *<28>	0 (0.01) *<5>	0.11 (0.02) *<100>!#	0.13 (0.02) *<100>!#	0.15 (0.01) *<100>!#	0.15 (0.01) *<100>!#	0.12 (0.01) *<100>!#	0.41 (0.02) *<100>!#	0.41 (0.01) *<100>!#	0.42 (0.01) *<100>!#	0.43 (0.01) *<100>!#	0.42 (0.01) *<100>!#	0.69 (0.01) *<100>!#	0.68 (0.01) *<100>!#	0.69 (0.01) *<100>!#	0.69 (0.01) *<100>!#	0.69 (0.01) *<100>!#	0.82 (0.01) *<100>!#	0.83 (0.01) *<100>!#	0.84 (0.01) *<100>!#	0.84 (0.01) *<100>!#	0.84 (0) *<100>!#
mde_mi4_1	0.01 (0.02) *<38>	0.02 (0.01) *<49>	0.03 (0.01) *<79>	0.01 (0.01) *<8>	0 (0.01) *<4>	0.09 (0.02) *<100>!#	0.1 (0.02) *<100>!#	0.12 (0.01) *<100>!#	0.13 (0.01) *<100>!#	0.13 (0.01) *<100>!#	0.4 (0.02) *<100>!#	0.41 (0.02) *<100>!#	0.42 (0.01) *<100>!#	0.43 (0.01) *<100>!#	0.44 (0.01) *<100>!#	0.71 (0.02) *<100>!#	0.7 (0.01) *<100>!#	0.71 (0.01) *<100>!#	0.71 (0.01) *<100>!#	0.72 (0.01) *<100>!#	0.84 (0.02) *<100>!#	0.84 (0.01) *<100>!#	0.86 (0) *<100>!#	0.86 (0.01) *<100>!#	0.86 (0) *<100>!#
mde_mi4_2	0.01 (0.02) *<38>	0.02 (0.01) *<44>	0.03 (0.01) *<85>	0.01 (0.01) *<11>	0 (0.01) *<2>	0.1 (0.02) *<100>!#	0.1 (0.02) *<100>!#	0.13 (0.01) *<100>!#	0.13 (0.01) *<100>!#	0.13 (0.01) *<100>!#	0.4 (0.02) *<100>!#	0.4 (0.02) *<100>!#	0.42 (0.01) *<100>!#	0.43 (0.01) *<100>!#	0.44 (0.01) *<100>!#	0.71 (0.02) *<100>!#	0.7 (0.01) *<100>!#	0.71 (0.01) *<100>!#	0.71 (0.01) *<100>!#	0.72 (0.01) *<100>!#	0.84 (0.01) *<100>!#	0.84 (0.01) *<100>!#	0.86 (0.01) *<100>!#	0.86 (0) *<100>!#	0.86 (0) *<100>!#
mde_mi4_bias	-0.01 (0.04) *<5>	0 (0.02) *<22>	-0.01 (0.01) *<31>	0 (0.01) *<6>	0 (0.01) *<3>	-0.1 (0.04) *<99>	-0.1 (0.03) *<100>!#	-0.13 (0.01) *<100>!#	-0.14 (0.01) *<100>!#	-0.16 (0.01) *<100>!#	-0.46 (0.03) *<100>!#	-0.45 (0.02) *<100>!#	-0.47 (0.01) *<100>!#	-0.48 (0.01) *<100>!#	-0.49 (0.01) *<100>!#	-0.75 (0.02) *<100>!#	-0.74 (0.01) *<100>!#	-0.75 (0.01) *<100>!#	-0.75 (0.01) *<100>!#	-0.76 (0.01) *<100>!#	-0.87 (0.01) *<100>!#	-0.87 (0.01) *<100>!#	-0.88 (0) *<100>!#	-0.89 (0) *<100>!#	-0.89 (0) *<100>!#
mde_mi5	0.01 (0.02) *<35>	0.03 (0.01) *<70>	0.05 (0.01) *<100>	0.01 (0.01) *<23>	0 (0.01) *<1>	0.11 (0.02) *<100>!#	0.13 (0.01) *<100>!#	0.15 (0.01) *<100>!#	0.15 (0.01) *<100>!#	0.12 (0.01) *<100>!#	0.41 (0.02) *<100>!#	0.41 (0.01) *<100>!#	0.42 (0.01) *<100>!#	0.43 (0.01) *<100>!#	0.42 (0.01) *<100>!#	0.69 (0.02) *<100>!#	0.68 (0.01) *<100>!#	0.69 (0.01) *<100>!#	0.69 (0.01) *<100>!#	0.69 (0.01) *<100>!#	0.82 (0.01) *<100>!#	0.83 (0.01) *<100>!#	0.84 (0.01) *<100>!#	0.84 (0) *<100>!#	0.84 (0) *<100>!#
mde_mi5_1	0.01 (0.02) *<18>	0.02 (0.02) *<40>	0.03 (0.01) *<82>	0.01 (0.01) *<11>	0 (0.01) *<4>	0.09 (0.02) *<100>!#	0.11 (0.02) *<100>!#	0.12 (0.01) *<100>!#	0.13 (0.01) *<100>!#	0.13 (0.01) *<100>!#	0.41 (0.02) *<100>!#	0.41 (0.02) *<100>!#	0.42 (0.01) *<100>!#	0.43 (0.01) *<100>!#	0.44 (0.01) *<100>!#	0.7 (0.02) *<100>!#	0.7 (0.01) *<100>!#	0.71 (0.01) *<100>!#	0.71 (0.01) *<100>!#	0.72 (0.01) *<100>!#	0.84 (0.01) *<100>!#	0.84 (0.01) *<100>!#	0.86 (0.01) *<100>!#	0.86 (0) *<100>!#	0.86 (0.01) *<100>!#
mde_mi5_2	0.01 (0.02) *<38>	0.02 (0.01) *<54>	0.03 (0.01) *<81>	0.01 (0.01) *<10>	0 (0.01) *<2>	0.09 (0.03) *<100>!#	0.1 (0.02) *<100>!#	0.12 (0.01) *<100>!#	0.13 (0.01) *<100>!#	0.13 (0.01) *<100>!#	0.41 (0.02) *<100>!#	0.41 (0.02) *<100>!#	0.42 (0.01) *<100>!#	0.43 (0.01) *<100>!#	0.44 (0.01) *<100>!#	0.71 (0.02) *<100>!#	0.7 (0.01) *<100>!#	0.71 (0.01) *<100>!#	0.71 (0.01) *<100>!#	0.72 (0.01) *<100>!#	0.84 (0.01) *<100>!#	0.84 (0.01) *<100>!#	0.86 (0.01) *<100>!#	0.86 (0.01) *<100>!#	0.86 (0.01) *<100>!#
mde_mi5_bias	0 (0)	-0.01 (0.02) *<27>	-0.01 (0.01) *<30>	0 (0.01) *<7>	0 (0.01) *<1>	-0.1 (0.04) *<99>	-0.1 (0.02) *<100>!#	-0.12 (0.01) *<100>!#	-0.15 (0.01) *<100>!#	-0.16 (0.01) *<100>!#	-0.46 (0.03) *<100>!#	-0.45 (0.02) *<100>!#	-0.46 (0.01) *<100>!#	-0.48 (0.01) *<100>!#	-0.49 (0.01) *<100>!#	-0.75 (0.02) *<100>!#	-0.75 (0.01) *<100>!#	-0.75 (0.01) *<100>!#	-0.75 (0.01) *<100>!#	-0.76 (0.01) *<100>!#	-0.87 (0.01) *<100>!#	-0.87 (0.01) *<100>!#	-0.88 (0.01) *<100>!#	-0.89 (0) *<100>!#	-0.89 (0) *<100>!#
mde_mi6	0.02 (0.02) *<50>	0.03 (0.01) *<63>	0.05 (0.01) *<100>!@	0.01 (0.01) *<22>	0 (0.01) *<2>	0.11 (0.02) *<100>!#	0.12 (0.01) *<100>!#	0.15 (0.01) *<100>!#	0.15 (0.01) *<100>!#	0.12 (0.01) *<100>!#	0.41 (0.02) *<100>!#	0.41 (0.01) *<100>!#	0.42 (0.01) *<100>!#	0.43 (0.01) *<100>!#	0.42 (0.01) *<100>!#	0.69 (0.01) *<100>!#	0.68 (0.01) *<100>!#	0.69 (0.01) *<100>!#	0.69 (0.01) *<100>!#	0.69 (0.01) *<100>!#	0.82 (0.01) *<100>!#	0.83 (0.01) *<100>!#	0.84 (0.01) *<100>!#	0.84 (0) *<100>!#	0.84 (0) *<100>!#
mde_mi6_1	0.01 (0.02) *<38>	0.02 (0.01) *<41>	0.03 (0.01) *<82>	0.01 (0.01) *<9>	0 (0.01) *<5>	0.09 (0.02) *<100>!#	0.1 (0.02) *<100>!#	0.12 (0.01) *<100>!#	0.13 (0.01) *<100>!#	0.13 (0.01) *<100>!#	0.4 (0.02) *<100>!#	0.41 (0.02) *<100>!#	0.42 (0.01) *<100>!#	0.43 (0.01) *<100>!#	0.44 (0.01) *<100>!#	0.7 (0.02) *<100>!#	0.7 (0.01) *<100>!#	0.71 (0.01) *<100>!#	0.71 (0.01) *<100>!#	0.72 (0.01) *<100>!#	0.84 (0.01) *<100>!#	0.85 (0.01) *<100>!#	0.86 (0.01) *<100>!#	0.86 (0) *<100>!#	0.86 (0) *<100>!#
mde_mi6_2	0.01 (0.02) *<30>	0.02 (0.01) *<45>	0.03 (0.01) *<88>	0.01 (0.01) *<14>	0 (0.01) *<5>	0.09 (0.03) *<100>!@	0.1 (0.02) *<100>!#	0.12 (0.01) *<100>!#	0.13 (0.01) *<100>!#	0.13 (0.01) *<100>!#	0.4 (0.02) *<100>!#	0.41 (0.02) *<100>!#	0.42 (0.01) *<100>!#	0.43 (0.01) *<100>!#	0.44 (0.01) *<100>!#	0.71 (0.02) *<100>!#	0.7 (0.01) *<100>!#	0.71 (0.01) *<100>!#	0.71 (0.01) *<100>!#	0.72 (0.01) *<100>!#	0.84 (0.01) *<100>!#	0.85 (0.01) *<100>!#	0.86 (0.01) *<100>!#	0.86 (0) *<100>!#	0.86 (0.01) *<100>!#
mde_mi6_bias	-0.01 (0.03) *<5>	0 (0.01) *<21>	-0.01 (0.01) *<32>	-0.01 (0.01) *<10>	0 (0.01) *<4>	-0.09 (0.04) *<95>	-0.1 (0.02) *<100>!#	-0.12 (0.01) *<100>!#	-0.15 (0.01) *<100>!#	-0.15 (0.01) *<100>!#	-0.46 (0.03) *<100>!#	-0.46 (0.02) *<100>!#	-0.47 (0.01) *<100>!#	-0.48 (0.01) *<100>!#	-0.49 (0.01) *<100>!#	-0.75 (0.02) *<100>!#	-0.74 (0.01) *<100>!#	-0.75 (0.01) *<100>!#	-0.75 (0.01) *<100>!#	-0.76 (0.01) *<100>!#	-0.87 (0.01) *<100>!#	-0.87 (0.01) *<100>!#	-0.88 (0.01) *<100>!#	-0.89 (0) *<100>!#	-0.89 (0) *<100>!#
mde_mi7	0.01 (0.02) *<38>	0.03 (0.01) *<66>	0.05 (0.01) *<100>	0.01 (0.01) *<27>	0 (0.01) *<6>	0.11 (0.02) *<100>!#	0.12 (0.02) *<100>!#	0.15 (0.01) *<100>!#	0.15 (0.01) *<100>!#	0.12 (0.01) *<100>!#	0.41 (0.02) *<100>!#	0.41 (0.01) *<100>!#	0.42 (0.01) *<100>!#	0.43 (0.01) *<100>!#	0.42 (0.01) *<100>!#	0.69 (0.02) *<100>!#	0.68 (0.01) *<100>!#	0.69 (0.01) *<100>!#	0.69 (0.01) *<100>!#	0.69 (0.01) *<100>!#	0.82 (0.01) *<100>!#	0.83 (0.01) *<100>!#	0.84 (0.01) *<100>!#	0.85 (0.01) *<100>!#	0.84 (0) *<100>!#
mde_mi7_1	0.01 (0.02) *<30>	0.02 (0.01) *<44>	0.03 (0.01) *<79>	0.01 (0.01) *<9>	0 (0.01) *<12>	0.09 (0.03) *<100>	0.1 (0.02) *<100>!#	0.12 (0.01) *<100>!#	0.13 (0.01) *<100>!#	0.13 (0.01) *<100>!#	0.41 (0.02) *<100>!#	0.4 (0.01) *<100>!#	0.42 (0.01) *<100>!#	0.43 (0.01) *<100>!#	0.44 (0.01) *<100>!#	0.7 (0.02) *<100>!#	0.7 (0.01) *<100>!#	0.71 (0.01) *<100>!#	0.71 (0.01) *<100>!#	0.72 (0.01) *<100>!#	0.84 (0.01) *<100>!#	0.85 (0.01) *<100>!#	0.86 (0.01) *<100>!#	0.86 (0.01) *<100>!#	0.86 (0.01) *<100>!#
mde_mi7_2	0.01 (0.02) *<28>	0.02 (0.01) *<43>	0.03 (0.01) *<90>	0.01 (0.01) *<5>	0 (0.01) *<3>	0.1 (0.03) *<100>!@	0.1 (0.02) *<100>!#	0.12 (0.01) *<100>!#	0.13 (0.01) *<100>!#	0.13 (0.01) *<100>!#	0.4 (0.02) *<100>!#	0.41 (0.02) *<100>!#	0.42 (0.01) *<100>!#	0.43 (0.01) *<100>!#	0.44 (0.01) *<100>!#	0.71 (0.02) *<100>!#	0.7 (0.01) *<100>!#	0.71 (0.01) *<100>!#	0.71 (0.01) *<100>!#	0.72 (0.01) *<100>!#	0.84 (0.01) *<100>!#	0.85 (0.01) *<100>!#	0.86 (0.01) *<100>!#	0.86 (0.01) *<100>!#	0.86 (0.01) *<100>!#
mde_mi7_bias	0 (0.03) *<2>	0 (0.01) *<22>	-0.01 (0.01) *<30>	0 (0.01) *<7>	0 (0.01) *<6>	-0.1 (0.04) *<96>	-0.1 (0.03) *<100>!#	-0.12 (0.01) *<100>!#	-0.15 (0.01) *<100>!#	-0.15 (0.01) *<100>!#	-0.46 (0.03) *<100>!#	-0.45 (0.02) *<100>!#	-0.47 (0.01) *<100>!#	-0.48 (0.01) *<100>!#	-0.49 (0.01) *<100>!#	-0.75 (0.02) *<100>!#	-0.74 (0.01) *<100>!#	-0.75 (0.01) *<100>!#	-0.75 (0.01) *<100>!#	-0.76 (0.01) *<100>!#	-0.87 (0.01) *<100>!#	-0.87 (0.01) *<100>!#	-0.88 (0.01) *<100>!#	-0.89 (0) *<100>!#	-0.88 (0.01) *<100>!#
mde_mi8	0.01 (0.02) *<30>	0.03 (0.01) *<67>	0.05 (0.01) *<98>	0.01 (0.01) *<28>	0 (0.01) *<9>	0.11 (0.02) *<100>!#	0.13 (0.01) *<100>!#	0.15 (0.01) *<100>!#	0.15 (0.01) *<100>!#	0.12 (0.01) *<100>!#	0.41 (0.02) *<100>!#	0.41 (0.01) *<100>!#	0.42 (0.01) *<100>!#	0.43 (0.01) *<100>!#	0.42 (0.01) *<100>!#	0.69 (0.02) *<100>!#	0.68 (0.01) *<100>!#	0.69 (0.01) *<100>!#	0.69 (0.01) *<100>!#	0.69 (0.01) *<100>!#	0.82 (0.01) *<100>!#	0.83 (0.01) *<100>!#	0.84 (0.01) *<100>!#	0.85 (0) *<100>!#	0.84 (0) *<100>!#
mde_mi8_1	0.01 (0.02) *<20>	0.02 (0.02) *<53>	0.03 (0.01) *<85>	0.01 (0.01) *<17>	0 (0.01) *<7>	0.09 (0.02) *<100>!#	0.1 (0.02) *<100>!#	0.12 (0.01) *<100>!#	0.13 (0.01) *<100>!#	0.12 (0.01) *<100>!#	0.4 (0.03) *<100>!#	0.41 (0.02) *<100>!#	0.42 (0.01) *<100>!#	0.43 (0.01) *<100>!#	0.44 (0.01) *<100>!#	0.7 (0.02) *<100>!#	0.7 (0.01) *<100>!#	0.71 (0.01) *<100>!#	0.71 (0.01) *<100>!#	0.72 (0.01) *<100>!#	0.84 (0.01) *<100>!#	0.85 (0.01) *<100>!#	0.86 (0.01) *<100>!#	0.86 (0) *<100>!#	0.86 (0) *<100>!#
mde_mi8_2	0.01 (0.02) *<30>	0.02 (0.02) *<44>	0.03 (0.01) *<80>	0.01 (0.01) *<12>	0 (0.01) *<7>	0.09 (0.02) *<100>!#	0.1 (0.02) *<100>!#	0.13 (0.01) *<100>!#	0.13 (0.01) *<100>!#	0.13 (0.01) *<100>!#	0.41 (0.02) *<100>!#	0.41 (0.02) *<100>!#	0.42 (0.01) *<100>!#	0.43 (0.01) *<100>!#	0.44 (0.01) *<100>!#	0.71 (0.02) *<100>!#	0.7 (0.01) *<100>!#	0.71 (0.01) *<100>!#	0.71 (0.01) *<100>!#	0.72 (0.01) *<100>!#	0.84 (0.01) *<100>!#	0.85 (0.01) *<100>!#	0.86 (0.01) *<100>!#	0.86 (0) *<100>!#	0.86 (0.01) *<100>!#
mde_mi8_bias	0 (0)	-0.01 (0.02) *<29>	-0.01 (0.01) *<26>	-0.01 (0.01) *<11>	0 (0.01) *<6>	-0.09 (0.04) *<97>	-0.11 (0.02) *<100>!#	-0.12 (0.01) *<100>!#	-0.14 (0.01) *<100>!#	-0.15 (0.01) *<100>!#	-0.46 (0.04) *<100>!#	-0.45 (0.02) *<100>!#	-0.47 (0.01) *<100>!#	-0.48 (0.01) *<100>!#	-0.49 (0.01) *<100>!#	-0.75 (0.02) *<100>!#	-0.74 (0.01) *<100>!#	-0.75 (0.01) *<100>!#	-0.75 (0.01) *<100>!#	-0.76 (0.01) *<100>!#	-0.86 (0.01) *<100>!#	-0.87 (0.01) *<100>!#	-0.88 (0.01) *<100>!#	-0.89 (0) *<100>!#	-0.89 (0.01) *<100>!#
mde_mi9	0.01 (0.02) *<32>	0.02 (0.02) *<53>	0.04 (0.01) *<95>	0.01 (0.01) *<17>	0 (0.01) *<4>	0.1 (0.02) *<100>!#	0.11 (0.02) *<100>!#	0.13 (0.01) *<100>!#	0.13 (0.01) *<100>!#	0.13 (0.01) *<100>!#	0.41 (0.02) *<100>!#	0.41 (0.02) *<100>!#	0.42 (0.01) *<100>!#	0.44 (0.01) *<100>!#	0.44 (0.01) *<100>!#	0.7 (0.02) *<100>!#	0.7 (0.01) *<100>!#	0.71 (0.01) *<100>!#	0.71 (0.01) *<100>!#	0.72 (0.01) *<100>!#	0.84 (0.01) *<100>!#	0.85 (0.01) *<100>!#	0.86 (0.01) *<100>!#	0.86 (0) *<100>!#	0.86 (0) *<100>!#
mde_bias1	-0.02 (0.01) *<70>	-0.05 (0.01) *<100>!@	-0.09 (0.01) *<100>!#	-0.02 (0.01) *<60>	0 (0.01) *<5>	-0.19 (0.02) *<100>!#	-0.21 (0.01) *<100>!#	-0.28 (0.01) *<100>!#	-0.28 (0.01) *<100>!#	-0.25 (0.01) *<100>!#	-0.55 (0.02) *<100>!#	-0.55 (0.01) *<100>!#	-0.58 (0.01) *<100>!#	-0.6 (0.01) *<100>!#	-0.6 (0.01) *<100>!#	-0.78 (0.01) *<100>!#	-0.78 (0.01) *<100>!#	-0.79 (0.01) *<100>!#	-0.8 (0.01) *<100>!#	-0.8 (0) *<100>!#	-0.88 (0.01) *<100>!#	-0.89 (0.01) *<100>!#	-0.9 (0) *<100>!#	-0.9 (0) *<100>!#	-0.9 (0) *<100>!#
mde_bias2	-0.04 (0.01) *<100>	-0.06 (0.01) *<100>!#	-0.12 (0.01) *<100>!#	-0.03 (0.01) *<73>	0 (0.01) *<6>	-0.19 (0.02) *<100>!#	-0.22 (0.01) *<100>!#	-0.28 (0.01) *<100>!#	-0.28 (0.01) *<100>!#	-0.26 (0.01) *<100>!#	-0.53 (0.02) *<100>!#	-0.54 (0.01) *<100>!#	-0.57 (0.01) *<100>!#	-0.59 (0.01) *<100>!#	-0.59 (0.01) *<100>!#	-0.78 (0.01) *<100>!#	-0.77 (0.01) *<100>!#	-0.78 (0.01) *<100>!#	-0.79 (0) *<100>!#	-0.79 (0) *<100>!#	-0.88 (0.01) *<100>!#	-0.88 (0.01) *<100>!#	-0.89 (0) *<100>!#	-0.9 (0) *<100>!#	-0.9 (0) *<100>!#
mde_bias	0.01 (0)	0 (0)	-0.09 (0.01) *<100>!#	-0.12 (0.01) *<100>!#	-0.1 (0.01) *<100>!#	0.09 (0.01) *<100>!#	0.09 (0.01) *<100>!#	0.1 (0.01) *<100>!#	0.13 (0.01) *<100>!#	0.14 (0.01) *<100>!#	0.46 (0.02) *<100>!#	0.47 (0.01) *<100>!#	0.5 (0.01) *<100>!#	0.52 (0.01) *<100>!#	0.53 (0.01) *<100>!#	0.75 (0.01) *<100>!#	0.75 (0.01) *<100>!#	0.76 (0.01) *<100>!#	0.77 (0.01) *<100>!#	0.77 (0.01) *<100>!#	0.87 (0.01) *<100>!#	0.87 (0.01) *<100>!#	0.89 (0) *<100>!#	0.89 (0) *<100>!#	0.89 (0) *<100>!#

The correlations between major depressive episodes and the symptoms of major depressive episodes, dysthymic disorder, and manic episodes are shown in Tables 3-5, respectively. Major depressive episodes were not significantly correlated with their symptoms in all of the 100 simulations when their symptoms were assumed not to correlate with each other (assumed correlation coefficients = 0), except for the two symptoms in the major criteria for the diagnosis of major depressive episodes (p < 0.05 for all, variable = mde_ma1 and mde_ma2, “depressed mood for more than two weeks” and “loss of interest or pleasure in daily activities for more than two weeks”, respectively) in Table 3. The correlations between major depressive episodes and these two symptoms were negligible (coefficients < 0.1) to moderate (coefficients ranging from 0.05 to 0.64, p < 0.05 for all), assuming symptoms were not correlated. Assuming symptoms were correlated with each other (assumed coefficients = 0.1, 0.4, 0.7, or 0.9), major depressive episodes were significantly correlated with their symptoms in all simulations (p < 0.001 for all). The correlations between major depressive episodes and their symptoms could be strong or very strong, assuming symptom prevalence as 0.7 and symptom correlations as 0.9.

**Table 4:** Correlations between major depressive episodes and the input symptoms of dysthymic disorder by assumed symptom prevalence and correlations Mean (standard deviation) *<number> = the numbers of significant statistics (p < 0.05) in 100 simulations !# = p < 0.001 in all of the 100 simulations !@ = p < 0.001 in some of the 100 simulations Note: see Table 1 for variable definitions and Table 2 for the prevalence of major depressive episodes, dysthymic disorder, and manic episodes. dys = Dysthymic Disorder; dys_ma = Depressed mood most of the day for more days than not, for at least 2 years; dys_mi = Minor criteria (at least 2 items); dys_mi1 = Poor appetite or overeating; dys_mi1_1 = Poor appetite; dys_mi1_2 = Overeating; dys_mi1_bias = Information of the domain not explained by the input variables; dys_mi4 = Low self-esteem; dys_mi6 = Feelings of hopelessness; dys_mi_bias = Information of minor criteria not explained by input variables; dys_bias = Information of diagnosis not explained by major or minor criteria

Assumed symptom prevalence	0.05	0.1	0.3	0.5	0.7	0.05	0.1	0.3	0.5	0.7	0.05	0.1	0.3	0.5	0.7	0.05	0.1	0.3	0.5	0.7	0.05	0.1	0.3	0.5	0.7
Assumed symptom correlations	0	0	0	0	0	0.1	0.1	0.1	0.1	0.1	0.4	0.4	0.4	0.4	0.4	0.7	0.7	0.7	0.7	0.7	0.9	0.9	0.9	0.9	0.9
dys	0.01 (0.04) *<8>	0.01 (0.02) *<13>	0.01 (0.01) *<27>	0 (0.01) *<7>	0 (0.01) *<3>	0.13 (0.04) *<100>!#	0.13 (0.02) *<100>!#	0.14 (0.01) *<100>!#	0.14 (0.01) *<100>!#	0.13 (0.01) *<100>!#	0.47 (0.02) *<100>!#	0.46 (0.02) *<100>!#	0.46 (0.01) *<100>!#	0.46 (0.01) *<100>!#	0.46 (0.01) *<100>!#	0.74 (0.02) *<100>!#	0.73 (0.01) *<100>!#	0.73 (0.01) *<100>!#	0.74 (0.01) *<100>!#	0.74 (0.01) *<100>!#	0.85 (0.01) *<100>!#	0.86 (0.01) *<100>!#	0.87 (0.01) *<100>!#	0.88 (0) *<100>!#	0.87 (0) *<100>!#
dys_ma	0 (0.01) *<15>	0 (0.01) *<5>	0 (0.01) *<9>	0 (0.01) *<8>	0 (0.01) *<3>	0.08 (0.02) *<100>	0.09 (0.02) *<100>!#	0.11 (0.01) *<100>!#	0.12 (0.01) *<100>!#	0.12 (0.01) *<100>!#	0.4 (0.02) *<100>!#	0.4 (0.02) *<100>!#	0.42 (0.01) *<100>!#	0.43 (0.01) *<100>!#	0.43 (0.01) *<100>!#	0.7 (0.01) *<100>!#	0.7 (0.01) *<100>!#	0.71 (0.01) *<100>!#	0.71 (0.01) *<100>!#	0.72 (0.01) *<100>!#	0.84 (0.01) *<100>!#	0.85 (0.01) *<100>!#	0.86 (0.01) *<100>!#	0.86 (0) *<100>!#	0.86 (0.01) *<100>!#
dys_mi	0.02 (0.02) *<70>	0.03 (0.01) *<84>	0.05 (0.01) *<100>!@	0.01 (0.01) *<25>	0 (0.01) *<3>	0.15 (0.01) *<100>!#	0.16 (0.01) *<100>!#	0.17 (0.01) *<100>!#	0.13 (0.01) *<100>!#	0.07 (0.01) *<100>!#	0.42 (0.01) *<100>!#	0.41 (0.01) *<100>!#	0.4 (0.01) *<100>!#	0.38 (0.01) *<100>!#	0.34 (0.01) *<100>!#	0.67 (0.01) *<100>!#	0.66 (0.01) *<100>!#	0.66 (0.01) *<100>!#	0.65 (0.01) *<100>!#	0.64 (0.01) *<100>!#	0.8 (0.01) *<100>!#	0.81 (0.01) *<100>!#	0.82 (0) *<100>!#	0.82 (0) *<100>!#	0.82 (0) *<100>!#
dys_mi1	0 (0.01) *<8>	0 (0.01) *<3>	0 (0.01) *<4>	0 (0.01) *<5>	0 (0.01) *<4>	0.09 (0.02) *<100>!#	0.1 (0.01) *<100>!#	0.13 (0.01) *<100>!#	0.13 (0.01) *<100>!#	0.12 (0.01) *<100>!#	0.4 (0.02) *<100>!#	0.4 (0.01) *<100>!#	0.41 (0.01) *<100>!#	0.42 (0.01) *<100>!#	0.41 (0.01) *<100>!#	0.68 (0.02) *<100>!#	0.68 (0.01) *<100>!#	0.68 (0.01) *<100>!#	0.69 (0.01) *<100>!#	0.69 (0.01) *<100>!#	0.82 (0.01) *<100>!#	0.83 (0.01) *<100>!#	0.84 (0) *<100>!#	0.84 (0) *<100>!#	0.84 (0) *<100>!#
dys_mi1_1	0 (0.01) *<2>	0 (0.01) *<2>	0 (0.01) *<6>	0 (0.01) *<4>	0 (0.01) *<4>	0.08 (0.02) *<100>!#	0.09 (0.02) *<100>!#	0.11 (0.01) *<100>!#	0.12 (0.01) *<100>!#	0.13 (0.01) *<100>!#	0.4 (0.02) *<100>!#	0.4 (0.02) *<100>!#	0.41 (0.01) *<100>!#	0.43 (0.01) *<100>!#	0.44 (0.01) *<100>!#	0.7 (0.02) *<100>!#	0.7 (0.01) *<100>!#	0.71 (0.01) *<100>!#	0.71 (0.01) *<100>!#	0.72 (0.01) *<100>!#	0.84 (0.01) *<100>!#	0.84 (0.01) *<100>!#	0.86 (0.01) *<100>!#	0.86 (0) *<100>!#	0.86 (0.01) *<100>!#
dys_mi1_2	0 (0.01) *<8>	0 (0.01) *<7>	0 (0.01) *<2>	0 (0.01) *<9>	0 (0.01) *<8>	0.07 (0.02) *<100>!@	0.09 (0.02) *<100>!#	0.11 (0.01) *<100>!#	0.12 (0.01) *<100>!#	0.12 (0.01) *<100>!#	0.4 (0.02) *<100>!#	0.4 (0.02) *<100>!#	0.42 (0.01) *<100>!#	0.43 (0.01) *<100>!#	0.43 (0.01) *<100>!#	0.7 (0.02) *<100>!#	0.7 (0.01) *<100>!#	0.71 (0.01) *<100>!#	0.71 (0.01) *<100>!#	0.72 (0.01) *<100>!#	0.84 (0.01) *<100>!#	0.85 (0.01) *<100>!#	0.86 (0.01) *<100>!#	0.86 (0) *<100>!#	0.86 (0) *<100>!#
dys_mi1_bias	0 (0)	0 (0.01) *<13>	0 (0.01) *<2>	0 (0.01) *<6>	0 (0.01) *<7>	-0.08 (0.04) *<94>	-0.09 (0.02) *<100>!#	-0.12 (0.01) *<100>!#	-0.14 (0.01) *<100>!#	-0.15 (0.01) *<100>!#	-0.46 (0.03) *<100>!#	-0.45 (0.02) *<100>!#	-0.46 (0.01) *<100>!#	-0.48 (0.01) *<100>!#	-0.49 (0.01) *<100>!#	-0.75 (0.02) *<100>!#	-0.74 (0.01) *<100>!#	-0.75 (0.01) *<100>!#	-0.75 (0.01) *<100>!#	-0.76 (0.01) *<100>!#	-0.86 (0.01) *<100>!#	-0.87 (0.01) *<100>!#	-0.88 (0.01) *<100>!#	-0.88 (0) *<100>!#	-0.88 (0) *<100>!#
dys_mi4	0 (0.01) *<15>	0 (0.01) *<4>	0 (0.01) *<5>	0 (0.01) *<7>	0 (0.01) *<4>	0.08 (0.03) *<100>!@	0.09 (0.01) *<100>!#	0.11 (0.01) *<100>!#	0.12 (0.01) *<100>!#	0.12 (0.01) *<100>!#	0.39 (0.02) *<100>!#	0.4 (0.02) *<100>!#	0.42 (0.01) *<100>!#	0.43 (0.01) *<100>!#	0.43 (0.01) *<100>!#	0.7 (0.02) *<100>!#	0.7 (0.01) *<100>!#	0.71 (0.01) *<100>!#	0.71 (0.01) *<100>!#	0.72 (0.01) *<100>!#	0.84 (0.01) *<100>!#	0.85 (0.01) *<100>!#	0.86 (0.01) *<100>!#	0.86 (0) *<100>!#	0.86 (0.01) *<100>!#
dys_mi6	0 (0.01) *<5>	0 (0.01) *<4>	0 (0.01) *<4>	0 (0.01) *<6>	0 (0.01) *<2>	0.08 (0.02) *<99>	0.08 (0.02) *<100>!#	0.11 (0.01) *<100>!#	0.12 (0.01) *<100>!#	0.12 (0.01) *<100>!#	0.4 (0.02) *<100>!#	0.4 (0.02) *<100>!#	0.42 (0.01) *<100>!#	0.43 (0.01) *<100>!#	0.43 (0.01) *<100>!#	0.7 (0.02) *<100>!#	0.7 (0.01) *<100>!#	0.71 (0.01) *<100>!#	0.71 (0.01) *<100>!#	0.72 (0.01) *<100>!#	0.84 (0.01) *<100>!#	0.85 (0.01) *<100>!#	0.86 (0.01) *<100>!#	0.86 (0) *<100>!#	0.86 (0.01) *<100>!#
dys_mi_bias	-0.01 (0.01) *<18>	-0.03 (0.01) *<87>	-0.05 (0.01) *<100>!@	-0.01 (0.01) *<23>	0 (0.01) *<3>	-0.18 (0.03) *<100>!#	-0.2 (0.02) *<100>!#	-0.25 (0.01) *<100>!#	-0.26 (0.01) *<100>!#	-0.24 (0.01) *<100>!#	-0.56 (0.02) *<100>!#	-0.56 (0.01) *<100>!#	-0.59 (0.01) *<100>!#	-0.6 (0.01) *<100>!#	-0.6 (0.01) *<100>!#	-0.8 (0.01) *<100>!#	-0.79 (0.01) *<100>!#	-0.8 (0.01) *<100>!#	-0.81 (0) *<100>!#	-0.81 (0) *<100>!#	-0.89 (0.01) *<100>!#	-0.9 (0.01) *<100>!#	-0.91 (0) *<100>!#	-0.91 (0) *<100>!#	-0.91 (0) *<100>!#
dys_bias	-0.02 (0.02) *<57>	-0.03 (0.01) *<71>	-0.04 (0.01) *<99>	-0.01 (0.01) *<21>	0 (0.01)	-0.13 (0.01) *<100>!#	-0.14 (0.01) *<100>!#	-0.15 (0.01) *<100>!#	-0.11 (0.01) *<100>!#	-0.06 (0.01) *<100>!#	-0.39 (0.01) *<100>!#	-0.39 (0.01) *<100>!#	-0.38 (0.01) *<100>!#	-0.36 (0) *<100>!#	-0.32 (0.01) *<100>!#	-0.65 (0.01) *<100>!#	-0.64 (0.01) *<100>!#	-0.64 (0.01) *<100>!#	-0.63 (0.01) *<100>!#	-0.62 (0.01) *<100>!#	-0.79 (0.01) *<100>!#	-0.79 (0.01) *<100>!#	-0.81 (0) *<100>!#	-0.81 (0) *<100>!#	-0.8 (0.01) *<100>!#

**Table 5:** Correlations between major depressive episodes and the input symptoms of manic episodes by assumed symptom prevalence and correlations Mean (standard deviation) *<number> = the numbers of significant statistics (p < 0.05) in 100 simulations !# = p < 0.001 in all of the 100 simulations !@ = p < 0.001 in the 100 simulations manic = Manic episodes for the diagnosis of bipolar disorder; man_ma1 = Elevated mood, lasting at least 1 week; man_ma2 = Expansive mood, lasting at least 1 week; man_ma3 = Irritable mood, lasting at least 1 week; man_mi1 = Increased self-esteem or grandiosity; man_mi1_1 = Increased self-esteem; man_mi1_2 = Grandiosity; man_mi1_bias = Information of the domain not explained by the input variables; man_mi2 = Decreased need for sleep (e.g., feels rested after only 3 hours of sleep); man_mi3 = More talkative than usual or pressure to keep talking; man_mi3_1 = More talkative than usual; man_mi3_2 = Pressure to keep talking; man_mi3_bias = Information of the domain not explained by the input variables; man_mi4 = Flight of ideas or subjective experience that thoughts are racing; man_mi4_1 = Flight of ideas; man_mi4_2 = Subjective experience that thoughts are racing; man_mi4_bias = Information of the domain not explained by the input variables; man_mi5 = Distractibility (i.e., attention too easily drawn to unimportant or irrelevant external stimuli); man_mi6 = Increase in goal-directed activity (either socially, at work or school, or sexually) or psychomotor agitation; man_mi6_1 = Increase in goal-directed activity ; man_mi6_2 = Psychomotor agitation; man_mi6_bias = Information of the domain not explained by the input variables; man_mi7 = Excessive involvement in pleasurable activities that have a high potential for painful consequences (e.g., engaging in unrestrained buying sprees, sexual indiscretions, or foolish business investments); man_bias1 = Information of diagnosis due to top-censoring for choosing at least three symptoms; man_bias2 = Information of diagnosis due to top-censoring for choosing at least four symptoms; man_bias = Information of diagnosis not explained by symptoms

Assumed symptom prevalence	0.05	0.1	0.3	0.5	0.7	0.05	0.1	0.3	0.5	0.7	0.05	0.1	0.3	0.5	0.7	0.05	0.1	0.3	0.5	0.7	0.05	0.1	0.3	0.5	0.7
Assumed symptom correlations	0	0	0	0	0	0.1	0.1	0.1	0.1	0.1	0.4	0.4	0.4	0.4	0.4	0.7	0.7	0.7	0.7	0.7	0.9	0.9	0.9	0.9	0.9
manic	0 (0)	0 (0.01) *<1>	0 (0.01) *<8>	0 (0.01) *<4>	0 (0.01) *<8>	0 (0.01) *<7>	0 (0.01) *<4>	0 (0.01) *<7>	0 (0.01) *<7>	0 (0.01) *<5>	0 (0.01) *<4>	0 (0.01) *<9>	0 (0.01) *<4>	0 (0.01) *<5>	0 (0.01) *<6>	0 (0.01) *<3>	0 (0.01) *<3>	0 (0.01) *<7>	0 (0.01) *<5>	0 (0.01) *<4>	0 (0.01) *<5>	0 (0.01) *<7>	0 (0.01) *<3>	0 (0.01)	0 (0.01) *<4>
man_ma1	0 (0.01) *<10>	0 (0.01) *<1>	0 (0.01) *<2>	0 (0.01) *<3>	0 (0.01) *<6>	0 (0.01) *<3>	0 (0.01) *<6>	0 (0.01) *<7>	0 (0.01) *<7>	0 (0.01) *<5>	0 (0.01) *<8>	0 (0.01) *<7>	0 (0.01) *<4>	0 (0.01) *<7>	0 (0.01) *<8>	0 (0.01) *<1>	0 (0.01) *<4>	0 (0.01) *<8>	0 (0.01) *<9>	0 (0.01) *<5>	0 (0.01) *<7>	0 (0.01) *<6>	0 (0.01) *<3>	0 (0.01) *<1>	0 (0.01) *<3>
man_ma2	0 (0.01) *<2>	0 (0.01) *<3>	0 (0.01) *<5>	0 (0.01) *<12>	0 (0.01) *<6>	0 (0.01) *<2>	0 (0.01) *<5>	0 (0.01) *<8>	0 (0.01) *<10>	0 (0.01) *<5>	0 (0.01) *<5>	0 (0.01) *<3>	0 (0.01) *<2>	0 (0.01) *<8>	0 (0.01) *<6>	0 (0.01) *<2>	0 (0.01) *<5>	0 (0.01) *<9>	0 (0.01) *<4>	0 (0.01) *<4>	0 (0.01) *<6>	0 (0.01) *<8>	0 (0.01) *<2>	0 (0.01) *<2>	0 (0.01) *<5>
man_ma3	0 (0.01) *<8>	0 (0.01) *<3>	0 (0.01) *<2>	0 (0.01) *<3>	0 (0.01) *<7>	0 (0.01) *<3>	0 (0.01) *<8>	0 (0.01) *<6>	0 (0.01) *<5>	0 (0.01) *<6>	0 (0.01)	0 (0.01) *<5>	0 (0.01) *<4>	0 (0.01) *<7>	0 (0.01) *<2>	0 (0.01) *<6>	0 (0.01) *<3>	0 (0.01) *<7>	0 (0.01) *<6>	0 (0.01) *<1>	0 (0.01) *<4>	0 (0.01) *<8>	0 (0.01) *<3>	0 (0.01) *<1>	0 (0.01) *<3>
man_mi1	0 (0.01) *<8>	0 (0.01) *<5>	0 (0.01) *<3>	0 (0.01) *<8>	0 (0.01) *<5>	0 (0.01) *<11>	0 (0.01) *<6>	0 (0.01) *<4>	0 (0.01) *<3>	0 (0.01) *<2>	0 (0.01) *<5>	0 (0.01) *<5>	0 (0.01) *<6>	0 (0.01) *<5>	0 (0.01) *<8>	0 (0.01) *<6>	0 (0.01) *<3>	0 (0.01) *<9>	0 (0.01) *<3>	0 (0.01) *<6>	0 (0.01) *<8>	0 (0.01) *<5>	0 (0.01) *<3>	0 (0.01) *<1>	0 (0.01) *<3>
man_mi1_1	0 (0.01) *<8>	0 (0.01) *<6>	0 (0.01) *<7>	0 (0.01) *<10>	0 (0.01) *<6>	0 (0.01) *<4>	0 (0.01) *<6>	0 (0.01) *<9>	0 (0.01) *<7>	0 (0.01) *<6>	0 (0.01) *<8>	0 (0.01) *<8>	0 (0.01) *<6>	0 (0.01) *<4>	0 (0.01) *<7>	0 (0.01) *<2>	0 (0.01) *<6>	0 (0.01) *<8>	0 (0.01) *<5>	0 (0.01) *<3>	0 (0.01) *<4>	0 (0.01) *<6>	0 (0.01) *<3>	0 (0.01) *<1>	0 (0.01) *<2>
man_mi1_2	0 (0.01) *<8>	0 (0.01) *<4>	0 (0.01) *<4>	0 (0.01) *<6>	0 (0.01) *<4>	0 (0.01) *<8>	0 (0.01) *<6>	0 (0.01) *<3>	0 (0.01) *<6>	0 (0.01) *<5>	0 (0.01) *<6>	0 (0.01) *<6>	0 (0.01) *<1>	0 (0.01) *<4>	0 (0.01) *<8>	0 (0.01) *<7>	0 (0.01) *<3>	0 (0.01) *<5>	0 (0.01) *<5>	0 (0.01) *<5>	0 (0.01) *<7>	0 (0.01) *<10>	0 (0.01) *<1>	0 (0.01)	0 (0.01) *<3>
man_mi1_bias	0 (0)	0 (0.01) *<12>	0 (0.01) *<5>	0 (0.01) *<4>	0 (0.01) *<6>	0 (0.01) *<6>	0 (0.01) *<9>	0 (0.01) *<4>	0 (0.01) *<2>	0 (0.01) *<4>	0 (0.01) *<9>	0 (0.01) *<11>	0 (0.01) *<3>	0 (0.01) *<4>	0 (0.01) *<3>	0 (0.01) *<4>	0 (0.01) *<3>	0 (0.01) *<6>	0 (0.01) *<7>	0 (0.01) *<4>	0 (0.01) *<5>	0 (0.01) *<9>	0 (0.01) *<4>	0 (0.01)	0 (0.01) *<2>
man_mi2	0 (0.01) *<5>	0 (0.01) *<2>	0 (0.01) *<4>	0 (0.01) *<3>	0 (0.01) *<3>	0 (0.01) *<3>	0 (0.01) *<5>	0 (0.01) *<4>	0 (0.01) *<6>	0 (0.01) *<9>	0 (0.01) *<8>	0 (0.01) *<7>	0 (0.01) *<8>	0 (0.01) *<4>	0 (0.01) *<8>	0 (0.01) *<3>	0 (0.01) *<4>	0 (0.01) *<10>	0 (0.01) *<5>	0 (0.01) *<4>	0 (0.01) *<6>	0 (0.01) *<10>	0 (0.01) *<3>	0 (0.01) *<2>	0 (0.01) *<3>
man_mi3	0 (0.01) *<10>	0 (0.01) *<9>	0 (0.01) *<8>	0 (0.01) *<4>	0 (0.01) *<5>	0 (0.01) *<1>	0 (0.01) *<3>	0 (0.01) *<7>	0 (0.01) *<5>	0 (0.01) *<4>	0 (0.01) *<7>	0 (0.01) *<9>	0 (0.01) *<3>	0 (0.01) *<9>	0 (0.01) *<8>	0 (0.01) *<2>	0 (0.01) *<6>	0 (0.01) *<6>	0 (0.01) *<5>	0 (0.01) *<4>	0 (0.01) *<6>	0 (0.01) *<4>	0 (0.01) *<4>	0 (0.01) *<1>	0 (0.01) *<3>
man_mi3_1	0 (0.01) *<5>	0 (0.01) *<7>	0 (0.01) *<6>	0 (0.01) *<6>	0 (0.01) *<5>	0 (0.01) *<3>	0 (0.01) *<5>	0 (0.01) *<10>	0 (0.01) *<6>	0 (0.01) *<6>	0 (0.01) *<6>	0 (0.01) *<7>	0 (0.01) *<6>	0 (0.01) *<7>	0 (0.01) *<3>	0 (0.01) *<1>	0 (0.01) *<3>	0 (0.01) *<9>	0 (0.01) *<4>	0 (0.01) *<5>	0 (0.01) *<5>	0 (0.01) *<6>	0 (0.01) *<3>	0 (0.01) *<2>	0 (0.01) *<1>
man_mi3_2	0 (0.01) *<8>	0 (0.01) *<6>	0 (0.01) *<7>	0 (0.01) *<8>	0 (0.01) *<2>	0 (0.01) *<3>	0 (0.01) *<6>	0 (0.01) *<6>	0 (0.01) *<8>	0 (0.01) *<4>	0 (0.01) *<7>	0 (0.01) *<10>	0 (0.01) *<7>	0 (0.01) *<5>	0 (0.01) *<6>	0 (0.01) *<5>	0 (0.01) *<4>	0 (0.01) *<1>	0 (0.01) *<8>	0 (0.01) *<5>	0 (0.01) *<9>	0 (0.01) *<7>	0 (0.01) *<2>	0 (0.01)	0 (0.01) *<3>
man_mi3_bias	0 (0.03) *<2>	0 (0.01) *<12>	0 (0.01) *<6>	0 (0.01) *<5>	0 (0.01) *<4>	0 (0.01) *<3>	0 (0.01) *<3>	0 (0.01) *<9>	0 (0.01) *<4>	0 (0.01) *<5>	0 (0.01) *<5>	0 (0.01) *<5>	0 (0.01) *<8>	0 (0.01) *<7>	0 (0.01) *<5>	0 (0.01) *<3>	0 (0.01) *<2>	0 (0.01) *<7>	0 (0.01) *<6>	0 (0.01) *<4>	0 (0.01) *<6>	0 (0.01) *<10>	0 (0.01) *<5>	0 (0.01) *<1>	0 (0.01) *<1>
man_mi4	0 (0.01) *<10>	0 (0.01) *<2>	0 (0.01) *<5>	0 (0.01) *<1>	0 (0.01) *<5>	0 (0.01) *<3>	0 (0.01) *<8>	0 (0.01) *<7>	0 (0.01) *<4>	0 (0.01) *<7>	0 (0.01) *<6>	0 (0.01) *<9>	0 (0.01) *<2>	0 (0.01) *<3>	0 (0.01) *<4>	0 (0.01) *<5>	0 (0.01) *<3>	0 (0.01) *<4>	0 (0.01) *<4>	0 (0.01) *<3>	0 (0.01) *<5>	0 (0.01) *<5>	0 (0.01) *<5>	0 (0.01) *<3>	0 (0.01) *<5>
man_mi4_1	0 (0.01) *<10>	0 (0.01) *<1>	0 (0.01) *<6>	0 (0.01) *<4>	0 (0.01) *<8>	0 (0.01) *<2>	0 (0.01) *<4>	0 (0.01) *<5>	0 (0.01) *<4>	0 (0.01) *<6>	0 (0.01) *<9>	0 (0.01) *<5>	0 (0.01) *<4>	0 (0.01) *<3>	0 (0.01) *<5>	0 (0.01) *<5>	0 (0.01) *<2>	0 (0.01) *<3>	0 (0.01) *<7>	0 (0.01) *<4>	0 (0.01) *<5>	0 (0.01) *<8>	0 (0.01) *<5>	0 (0.01) *<2>	0 (0.01) *<4>
man_mi4_2	0 (0.01) *<8>	0 (0.01) *<5>	0 (0.01) *<4>	0 (0.01) *<4>	0 (0.01) *<7>	0 (0.01) *<5>	0 (0.01) *<5>	0 (0.01) *<4>	0 (0.01) *<6>	0 (0.01) *<8>	0 (0.01) *<8>	0 (0.01) *<5>	0 (0.01) *<3>	0 (0.01) *<3>	0 (0.01) *<4>	0 (0.01) *<4>	0 (0.01) *<7>	0 (0.01) *<2>	0 (0.01) *<4>	0 (0.01) *<4>	0 (0.01) *<6>	0 (0.01) *<7>	0 (0.01) *<3>	0 (0.01) *<2>	0 (0.01) *<4>
man_mi4_bias	0 (0)	0 (0.01) *<8>	0 (0.01) *<5>	0 (0.01) *<7>	0 (0.01) *<3>	0 (0.01) *<3>	0 (0.01) *<3>	0 (0.01) *<2>	0 (0.01) *<5>	0 (0.01) *<3>	0 (0.01) *<8>	0 (0.01) *<2>	0 (0.01) *<5>	0 (0.01) *<4>	0 (0.01) *<4>	0 (0.01) *<9>	0 (0.01) *<4>	0 (0.01) *<4>	0 (0.01) *<9>	0 (0.01) *<4>	0 (0.01) *<9>	0 (0.01) *<8>	0 (0.01) *<3>	0 (0.01)	0 (0.01) *<4>
man_mi5	0 (0.01) *<5>	0 (0.01) *<6>	0 (0.01) *<1>	0 (0.01) *<2>	0 (0.01) *<4>	0 (0.01) *<2>	0 (0.01) *<4>	0 (0.01) *<6>	0 (0.01) *<6>	0 (0.01) *<2>	0 (0.01) *<5>	0 (0.01) *<6>	0 (0.01) *<4>	0 (0.01) *<5>	0 (0.01) *<6>	0 (0.01) *<4>	0 (0.01) *<4>	0 (0.01) *<6>	0 (0.01) *<4>	0 (0.01) *<5>	0 (0.01) *<6>	0 (0.01) *<6>	0 (0.01) *<3>	0 (0.01) *<2>	0 (0.01) *<3>
man_mi6	0 (0.01) *<8>	0 (0.01) *<2>	0 (0.01) *<7>	0 (0.01) *<7>	0 (0.01) *<1>	0 (0.01) *<6>	0 (0.01) *<4>	0 (0.01) *<3>	0 (0.01) *<6>	0 (0.01) *<3>	0 (0.01) *<7>	0 (0.01) *<6>	0 (0.01) *<9>	0 (0.01) *<6>	0 (0.01) *<6>	0 (0.01) *<4>	0 (0.01) *<4>	0 (0.01) *<6>	0 (0.01) *<7>	0 (0.01) *<2>	0 (0.01) *<6>	0 (0.01) *<6>	0 (0.01) *<6>	0 (0.01) *<2>	0 (0.01) *<5>
man_mi6_1	0 (0.01) *<2>	0 (0.01) *<2>	0 (0.01) *<4>	0 (0.01) *<7>	0 (0.01) *<3>	0 (0.01) *<8>	0 (0.01) *<7>	0 (0.01) *<4>	0 (0.01) *<2>	0 (0.01) *<5>	0 (0.01) *<5>	0 (0.01) *<5>	0 (0.01) *<5>	0 (0.01) *<5>	0 (0.01) *<5>	0 (0.01) *<4>	0 (0.01) *<2>	0 (0.01) *<5>	0 (0.01) *<5>	0 (0.01) *<5>	0 (0.01) *<6>	0 (0.01) *<11>	0 (0.01) *<5>	0 (0.01)	0 (0.01) *<6>
man_mi6_2	0 (0.01) *<5>	0 (0.01) *<6>	0 (0.01) *<4>	0 (0.01) *<7>	0 (0.01) *<5>	0 (0.01) *<4>	0 (0.01) *<5>	0 (0.01) *<8>	0 (0.01) *<2>	0 (0.01) *<3>	0 (0.01) *<4>	0 (0.01) *<8>	0 (0.01) *<6>	0 (0.01) *<4>	0 (0.01) *<5>	0 (0.01) *<4>	0 (0.01) *<4>	0 (0.01) *<7>	0 (0.01) *<7>	0 (0.01) *<2>	0 (0.01) *<6>	0 (0.01) *<7>	0 (0.01) *<3>	0 (0.01)	0 (0.01) *<3>
man_mi6_bias	0 (0)	0 (0.01) *<11>	0 (0.01) *<4>	0 (0.01) *<7>	0 (0.01) *<8>	0 (0.01) *<3>	0 (0.01) *<3>	0 (0.01) *<8>	0 (0.01) *<4>	0 (0.01) *<5>	0 (0.01) *<3>	0 (0.01) *<7>	0 (0.01) *<4>	0 (0.01) *<5>	0 (0.01) *<8>	0 (0.01) *<5>	0 (0.01) *<4>	0 (0.01) *<7>	0 (0.01) *<8>	0 (0.01) *<4>	0 (0.01) *<6>	0 (0.01) *<9>	0 (0.01) *<2>	0 (0.01) *<1>	0 (0.01) *<2>
man_mi7	0 (0.01) *<5>	0 (0.01) *<7>	0 (0.01) *<8>	0 (0.01) *<6>	0 (0.01) *<1>	0 (0.01) *<7>	0 (0.01) *<4>	0 (0.01) *<4>	0 (0.01) *<4>	0 (0.01) *<5>	0 (0.01) *<8>	0 (0.01) *<7>	0 (0.01) *<4>	0 (0.01) *<6>	0 (0.01) *<4>	0 (0.01) *<4>	0 (0.01) *<1>	0 (0.01) *<6>	0 (0.01) *<4>	0 (0.01) *<5>	0 (0.01) *<7>	0 (0.01) *<6>	0 (0.01) *<5>	0 (0.01) *<4>	0 (0.01) *<4>
man_bias1	0 (0.01) *<5>	0 (0.01) *<6>	0 (0.01) *<4>	0 (0.01) *<5>	0 (0.01) *<7>	0 (0.01) *<9>	0 (0.01) *<6>	0 (0.01) *<6>	0 (0.01) *<6>	0 (0.01) *<7>	0 (0.01) *<9>	0 (0.01) *<8>	0 (0.01) *<4>	0 (0.01) *<3>	0 (0.01) *<6>	0 (0.01) *<4>	0 (0.01) *<3>	0 (0.01) *<7>	0 (0.01) *<3>	0 (0.01) *<5>	0 (0.01) *<7>	0 (0.01) *<8>	0 (0.01) *<4>	0 (0.01) *<1>	0 (0.01) *<2>
man_bias2	0 (0.01) *<2>	0 (0.01) *<6>	0 (0.01) *<6>	0 (0.01) *<6>	0 (0.01) *<8>	0 (0.01) *<10>	0 (0.01) *<7>	0 (0.01) *<5>	0 (0.01) *<7>	0 (0.01) *<7>	0 (0.01) *<10>	0 (0.01) *<9>	0 (0.01) *<5>	0 (0.01) *<2>	0 (0.01) *<8>	0 (0.01) *<4>	0 (0.01) *<3>	0 (0.01) *<7>	0 (0.01) *<4>	0 (0.01) *<5>	0 (0.01) *<8>	0 (0.01) *<8>	0 (0.01) *<4>	0 (0.01) *<1>	0 (0.01) *<2>
man_bias	0 (0.01) *<2>	0 (0.01) *<6>	0 (0.01) *<5>	0 (0.01) *<4>	0 (0.01) *<5>	0 (0.01) *<3>	0 (0.01) *<5>	0 (0.01) *<7>	0 (0.01) *<11>	0 (0.01) *<5>	0 (0.01) *<6>	0 (0.01) *<8>	0 (0.01) *<5>	0 (0.01) *<1>	0 (0.01) *<5>	0 (0.01) *<4>	0 (0.01) *<1>	0 (0.01) *<7>	0 (0.01) *<3>	0 (0.01) *<4>	0 (0.01) *<9>	0 (0.01) *<7>	0 (0.01) *<4>	0 (0.01) *<1>	0 (0.01) *<2>

In Table 4, the correlations between major depressive episodes and the symptoms for the diagnosis of dysthymic disorder were not significant in all simulations assuming symptoms were not correlated (assumed coefficients = 0), although six symptoms were shared by the diagnoses of major depressive episodes and dysthymic disorder. In contrast, the correlations between major depressive episodes and the symptoms of dysthymic disorder were all significant (p < 0.05 for all), assuming symptom correlations were 0.1, 0.4, 0.7, or 0.9. The correlations between major depressive episodes and the symptoms of dysthymic disorder could be strong at most, assuming symptom prevalence as 0.7 and symptom correlations as 0.9.

In Table 5, the correlations between major depressive episodes and the symptoms of manic episodes were negligible and not significant in all simulations under all combinations of assumed symptom prevalence and correlations.

In Tables 6-8, the odds ratios between major depressive episodes and psychiatric symptoms are shown. However, odds ratios were not available for symptoms with more than two categories or the symptoms that often occurred with major depressive episodes by causing 0 values in the denominators for the calculations of odds ratios.

**Table 6:** Odds ratios between major depressive episodes and their input symptoms by assumed symptom prevalence and correlations NA = not applicable due to 0 case in the denominators for the calculation of odds ratios Mean (standard deviation) *<number> = the numbers of significant statistics (p < 0.05) in 100 simulations !# = p < 0.001 in all of the 100 simulations !@ = p < 0.001 in the 100 simulations mde = Major Depressive Episodes for the diagnosis; mde_ma1 = Depressed mood for more than two weeks.; mde_ma2 = Loss of interest or pleasure in daily activities for more than two weeks.; mde_mi3 = Significant unintentional weight loss or gain; mde_mi3_1 = Significant unintentional weight gain; mde_mi3_2 = Significant unintentional weight loss; mde_mi3_bias = Information of the domain not explained by the input variables; mde_mi4 = Insomnia or sleeping too much; mde_mi4_1 = Insomnia; mde_mi4_2 = Sleeping too much; mde_mi4_bias = Information of the domain not explained by the input variables; mde_mi5 = Agitation or psychomotor retardation noticed by others; mde_mi5_1 = Agitation; mde_mi5_2 = Psychomotor retardation noticed by others; mde_mi5_bias = Information of the domain not explained by the input variables; mde_mi6 = Fatigue or loss of energy; mde_mi6_1 = Fatigue; mde_mi6_2 = Loss of energy; mde_mi6_bias = Information of the domain not explained by the input variables; mde_mi7 = Feelings of worthlessness or excessive guilt; mde_mi7_1 = Feelings of worthlessness; mde_mi7_2 = Feelings of excessive guilt; mde_mi7_bias = Information of the domain not explained by the input variables; mde_mi8 = Diminished ability to think or concentrate, or indecisiveness+; mde_mi8_1 = Diminished ability to think or concentrate; mde_mi8_2 = Indecisiveness; mde_mi8_bias = Information of the domain not explained by the input variables; mde_mi9 = Recurrent thoughts of death; mde_bias1 = Information due to top censoring by choosing three domains in minor criteria; mde_bias2 = Information due to top censoring by choosing four domains in minor criteria; mde_bias = Information of diagnosis not explained by the domain

Assumed symptom prevalence	0.05	0.1	0.3	0.5	0.7	0.05	0.1	0.3	0.5	0.7	0.05	0.1	0.3	0.5	0.7	0.05	0.1	0.3	0.5	0.7	0.05	0.1	0.3	0.5	0.7
Assumed symptom correlations	0	0	0	0	0	0.1	0.1	0.1	0.1	0.1	0.4	0.4	0.4	0.4	0.4	0.7	0.7	0.7	0.7	0.7	0.9	0.9	0.9	0.9	0.9
mde	NA	NA	NA	NA	NA	NA	NA	NA	NA	NA	NA	NA	NA	NA	NA	NA	NA	NA	NA	NA	NA	NA	NA	NA	NA
mde_ma1	NA	NA	NA	NA	NA	NA	NA	NA	NA	NA	NA	NA	NA	NA	NA	NA	NA	NA	NA	NA	NA	NA	NA	NA	NA
mde_ma2	NA	NA	NA	NA	NA	NA	NA	NA	NA	NA	NA	NA	NA	NA	NA	NA	NA	NA	NA	NA	NA	NA	NA	NA	NA
mde_mi3	NA	4.47 (2.51) *<56>	1.48 (0.12) *<100>	1.07 (0.06) *<28>	1 (0.07) *<4>	18.6 (7.47) *<100>!#	8.66 (1.83) *<100>!#	3.01 (0.22) *<100>!#	2.26 (0.14) *<100>!#	2.22 (0.15) *<100>!#	88.41 (19.68) *<100>!#	45.19 (6.39) *<100>!#	16.96 (1.46) *<100>!#	13.34 (0.94) *<100>!#	14.47 (1.08) *<100>!#	602.31 (149.79) *<100>!#	285.31 (51) *<100>!#	116.22 (14.63) *<100>!#	93.56 (10.37) *<100>!#	111.57 (11.09) *<100>!#	2542.06 (1053.63) *<100>!#	1351.1 (395) *<100>!#	663.56 (99.61) *<100>!#	562.95 (97.54) *<100>!#	635.52 (127.2) *<100>!#
mde_mi3_1	NA	3.3 (2.18) *<27>	1.3 (0.11) *<87>	1.03 (0.05) *<12>	1 (0.04) *<1>	13.04 (4.46) *<100>!#	6.26 (1.16) *<100>!#	2.33 (0.17) *<100>!#	1.8 (0.09) *<100>!#	1.74 (0.08) *<100>!#	53.47 (9.39) *<100>!#	26.48 (2.94) *<100>!#	10 (0.6) *<100>!#	7.65 (0.37) *<100>!#	7.88 (0.36) *<100>!#	291.3 (44.96) *<100>!#	143.88 (17.8) *<100>!#	57.59 (4.37) *<100>!#	45.82 (3.32) *<100>!#	51.98 (3.79) *<100>!#	1107.96 (296.23) *<100>!#	597.78 (83.7) *<100>!#	299.44 (31.45) *<100>!#	251.02 (23.61) *<100>!#	280.37 (32.4) *<100>!#
mde_mi3_2	NA	3.57 (2.7) *<26>	1.3 (0.11) *<84>	1.04 (0.05) *<12>	1 (0.05) *<6>	12.61 (4.22) *<100>	6.22 (1.22) *<100>!#	2.35 (0.16) *<100>!#	1.8 (0.09) *<100>!#	1.75 (0.08) *<100>!#	52.53 (8.8) *<100>!#	26.74 (2.86) *<100>!#	10.02 (0.63) *<100>!#	7.66 (0.41) *<100>!#	7.86 (0.37) *<100>!#	292.67 (47.4) *<100>!#	144.26 (19.26) *<100>!#	58.01 (5.02) *<100>!#	45.51 (2.97) *<100>!#	52.01 (3.58) *<100>!#	1091.04 (234.94) *<100>!#	608.13 (96.87) *<100>!#	299.6 (27.86) *<100>!#	250.11 (22.58) *<100>!#	285.73 (29.94) *<100>!#
mde_mi3_bias	NA	NA	0.82 (0.12) *<37>	0.97 (0.05) *<7>	1 (0.04) *<6>	0.05 (0.03) *<99>	0.11 (0.03) *<100>!@	0.36 (0.03) *<100>!#	0.51 (0.03) *<100>!#	0.54 (0.02) *<100>!#	0.01 (0) *<100>!#	0.03 (0) *<100>!#	0.08 (0.01) *<100>!#	0.11 (0.01) *<100>!#	0.11 (0.01) *<100>!#	0 (0) *<100>!#	0.01 (0) *<100>!#	0.01 (0) *<100>!#	0.02 (0) *<100>!#	0.02 (0) *<100>!#	0 (0) *<100>!#	0 (0) *<100>!#	0 (0) *<100>!#	0 (0) *<100>!#	0 (0) *<100>!#
mde_mi4	NA	4.93 (4.25) *<59>	1.44 (0.12) *<99>	1.08 (0.05) *<28>	1.01 (0.07) *<5>	19.77 (8.47) *<100>!#	8.73 (1.96) *<100>!#	2.98 (0.26) *<100>!#	2.28 (0.14) *<100>!#	2.26 (0.15) *<100>!#	88.44 (17.2) *<100>!#	44.56 (6.05) *<100>!#	16.89 (1.27) *<100>!#	13.3 (1.04) *<100>!#	14.58 (1.09) *<100>!#	604.27 (181.26) *<100>!#	289.97 (54.12) *<100>!#	118.12 (12.92) *<100>!#	95.67 (9.66) *<100>!#	112.59 (12.08) *<100>!#	2560.39 (1543.02) *<100>!#	1271.49 (323.25) *<100>!#	652.67 (108.66) *<100>!#	542.72 (77.74) *<100>!#	620.22 (93.58) *<100>!#
mde_mi4_1	NA	3.73 (2.84) *<35>	1.28 (0.11) *<79>	1.04 (0.05) *<8>	1 (0.05) *<4>	13.64 (5.45) *<100>!@	6.07 (1.27) *<100>!#	2.32 (0.18) *<100>!#	1.81 (0.09) *<100>!#	1.76 (0.08) *<100>!#	52.78 (8.92) *<100>!#	27.01 (2.89) *<100>!#	10.04 (0.61) *<100>!#	7.68 (0.38) *<100>!#	7.94 (0.38) *<100>!#	292.79 (47.94) *<100>!#	143.95 (19.33) *<100>!#	57.93 (4.22) *<100>!#	46.24 (2.83) *<100>!#	51.97 (3.57) *<100>!#	1106.12 (297.94) *<100>!#	586.98 (86.76) *<100>!#	301.03 (28.63) *<100>!#	248.59 (24.78) *<100>!#	282.5 (27.05) *<100>!#
mde_mi4_2	NA	3.32 (2.79) *<27>	1.28 (0.11) *<84>	1.04 (0.05) *<11>	1 (0.04) *<2>	13.5 (4.59) *<100>!#	6.32 (1.27) *<100>!#	2.35 (0.17) *<100>!#	1.8 (0.09) *<100>!#	1.74 (0.07) *<100>!#	53.28 (8.35) *<100>!#	26.31 (3.29) *<100>!#	10.03 (0.64) *<100>!#	7.71 (0.41) *<100>!#	7.89 (0.44) *<100>!#	293.41 (51.75) *<100>!#	142.34 (17.12) *<100>!#	57.89 (4.06) *<100>!#	46.24 (3.27) *<100>!#	51.92 (3.5) *<100>!#	1061.36 (222.02) *<100>!#	590.81 (101.13) *<100>!#	299.2 (29.96) *<100>!#	253.26 (25.66) *<100>!#	278.55 (23.53) *<100>!#
mde_mi4_bias	NA	NA	0.84 (0.11) *<31>	0.98 (0.05) *<6>	1 (0.04) *<3>	0.04 (0.03) *<99>	0.11 (0.03) *<100>!@	0.36 (0.03) *<100>!#	0.5 (0.03) *<100>!#	0.53 (0.02) *<100>!#	0.01 (0) *<100>!#	0.03 (0) *<100>!#	0.08 (0.01) *<100>!#	0.11 (0) *<100>!#	0.11 (0.01) *<100>!#	0 (0) *<100>!#	0.01 (0) *<100>!#	0.01 (0) *<100>!#	0.02 (0) *<100>!#	0.02 (0) *<100>!#	0 (0) *<100>!#	0 (0) *<100>!#	0 (0) *<100>!#	0 (0) *<100>!#	0 (0) *<100>!#
mde_mi5	NA	4.9 (3.11) *<62>	1.45 (0.12) *<100>	1.07 (0.05) *<24>	1 (0.07) *<1>	18.11 (7.69) *<100>!#	8.71 (1.94) *<100>!#	2.97 (0.2) *<100>!#	2.27 (0.13) *<100>!#	2.28 (0.16) *<100>!#	94.67 (23.66) *<100>!#	45.36 (7.38) *<100>!#	16.81 (1.44) *<100>!#	13.42 (0.91) *<100>!#	14.54 (1.05) *<100>!#	612.22 (193.51) *<100>!#	294.97 (53.93) *<100>!#	116.78 (12.74) *<100>!#	95.64 (10.67) *<100>!#	110.37 (10.77) *<100>!#	2571.79 (1329.97) *<100>!#	1263.99 (341.79) *<100>!#	669.08 (123.09) *<100>!#	540.76 (72.91) *<100>!#	646.99 (116.32) *<100>!#
mde_mi5_1	NA	3.37 (2.38) *<30>	1.28 (0.1) *<82>	1.04 (0.05) *<11>	1 (0.04) *<4>	12.89 (4.48) *<100>!@	6.33 (1.19) *<100>!#	2.3 (0.16) *<100>!#	1.82 (0.08) *<100>!#	1.74 (0.09) *<100>!#	54.54 (9.19) *<100>!#	26.59 (3.41) *<100>!#	9.98 (0.65) *<100>!#	7.65 (0.37) *<100>!#	7.92 (0.37) *<100>!#	289.26 (49.21) *<100>!#	145.47 (16.02) *<100>!#	57.86 (4.16) *<100>!#	46.07 (3.04) *<100>!#	52.05 (3.43) *<100>!#	1095.73 (240) *<100>!#	593.09 (94.13) *<100>!#	299.18 (34.13) *<100>!#	248.21 (23.19) *<100>!#	283.14 (28.06) *<100>!#
mde_mi5_2	NA	3.79 (2.79) *<34>	1.28 (0.1) *<81>	1.03 (0.05) *<10>	1 (0.04) *<2>	12.56 (5.5) *<100>!@	5.98 (1.25) *<100>!#	2.33 (0.12) *<100>!#	1.81 (0.08) *<100>!#	1.77 (0.08) *<100>!#	54.87 (8.4) *<100>!#	26.83 (3.18) *<100>!#	9.96 (0.63) *<100>!#	7.65 (0.35) *<100>!#	7.85 (0.38) *<100>!#	288.43 (45.66) *<100>!#	145.31 (16.57) *<100>!#	57.87 (4.22) *<100>!#	45.65 (3.54) *<100>!#	51.56 (3.7) *<100>!#	1119.14 (243.68) *<100>!#	591.49 (90.06) *<100>!#	303.76 (30.89) *<100>!#	249.51 (22.87) *<100>!#	282.48 (29.02) *<100>!#
mde_mi5_bias	NA	NA	0.84 (0.1) *<27>	0.98 (0.05) *<6>	1 (0.04) *<1>	0.05 (0.03) *<99>	0.11 (0.03) *<100>!@	0.36 (0.03) *<100>!#	0.5 (0.02) *<100>!#	0.54 (0.02) *<100>!#	0.01 (0) *<100>!#	0.03 (0) *<100>!#	0.08 (0.01) *<100>!#	0.11 (0.01) *<100>!#	0.11 (0.01) *<100>!#	0 (0) *<100>!#	0.01 (0) *<100>!#	0.01 (0) *<100>!#	0.02 (0) *<100>!#	0.02 (0) *<100>!#	0 (0) *<100>!#	0 (0) *<100>!#	0 (0) *<100>!#	0 (0) *<100>!#	0 (0) *<100>!#
mde_mi6	NA	4.8 (3.97) *<57>	1.48 (0.11) *<100>!@	1.07 (0.06) *<22>	1 (0.07) *<2>	18.46 (7.73) *<100>!#	8.45 (1.56) *<100>!#	2.92 (0.23) *<100>!#	2.3 (0.13) *<100>!#	2.27 (0.15) *<100>!#	90.75 (22.11) *<100>!#	44.93 (7.1) *<100>!#	17.07 (1.4) *<100>!#	13.35 (1) *<100>!#	14.7 (1.27) *<100>!#	596.96 (168.29) *<100>!#	283.21 (55.78) *<100>!#	116.68 (13.13) *<100>!#	95.61 (9.04) *<100>!#	112.7 (12.43) *<100>!#	2531.72 (1035.98) *<100>!#	1353.57 (461.6) *<100>!#	645.91 (98.59) *<100>!#	558.98 (93.97) *<100>!#	632.58 (103.33) *<100>!#
mde_mi6_1	NA	3.37 (2.29) *<30>	1.28 (0.11) *<82>	1.04 (0.05) *<9>	1 (0.05) *<5>	13.34 (4.47) *<100>!@	6.19 (1.25) *<100>!#	2.28 (0.17) *<100>!#	1.82 (0.09) *<100>!#	1.75 (0.07) *<100>!#	53.39 (9.46) *<100>!#	26.97 (3.25) *<100>!#	10.04 (0.63) *<100>!#	7.67 (0.38) *<100>!#	7.87 (0.42) *<100>!#	288.01 (48.61) *<100>!#	144.12 (17.77) *<100>!#	57.69 (3.86) *<100>!#	46.41 (3.28) *<100>!#	51.96 (3.81) *<100>!#	1073.59 (241.05) *<100>!#	600.61 (89.45) *<100>!#	299.55 (30.35) *<100>!#	250.66 (20.64) *<100>!#	284.49 (27.1) *<100>!#
mde_mi6_2	NA	3.37 (2.23) *<26>	1.31 (0.12) *<88>	1.03 (0.05) *<14>	1 (0.04) *<5>	12.19 (4.99) *<100>	6.28 (1.24) *<100>!#	2.32 (0.16) *<100>!#	1.83 (0.07) *<100>!#	1.75 (0.07) *<100>!#	54.24 (9.42) *<100>!#	26.59 (2.96) *<100>!#	10.03 (0.61) *<100>!#	7.67 (0.37) *<100>!#	7.9 (0.41) *<100>!#	291.88 (48.61) *<100>!#	142.26 (17.46) *<100>!#	57.97 (4.2) *<100>!#	45.86 (3.31) *<100>!#	52.08 (3.39) *<100>!#	1089.51 (241.81) *<100>!#	616.05 (91.46) *<100>!#	299.25 (30.58) *<100>!#	251.02 (22.46) *<100>!#	282.89 (29.52) *<100>!#
mde_mi6_bias	NA	NA	0.85 (0.11) *<30>	0.97 (0.05) *<10>	1.01 (0.04) *<4>	0.06 (0.05) *<93>	0.1 (0.03) *<100>!#	0.36 (0.03) *<100>!#	0.5 (0.02) *<100>!#	0.54 (0.02) *<100>!#	0.01 (0) *<100>!#	0.03 (0) *<100>!#	0.08 (0.01) *<100>!#	0.11 (0) *<100>!#	0.11 (0.01) *<100>!#	0 (0) *<100>!#	0.01 (0) *<100>!#	0.01 (0) *<100>!#	0.02 (0) *<100>!#	0.02 (0) *<100>!#	0 (0) *<100>!#	0 (0) *<100>!#	0 (0) *<100>!#	0 (0) *<100>!#	0 (0) *<100>!#
mde_mi7	NA	NA	1.47 (0.12) *<100>	1.08 (0.06) *<27>	1 (0.07) *<6>	18.39 (6.27) *<100>!#	8.49 (1.99) *<100>!#	2.97 (0.21) *<100>!#	2.28 (0.13) *<100>!#	2.27 (0.14) *<100>!#	94.66 (24.03) *<100>!#	45.53 (6.72) *<100>!#	16.98 (1.33) *<100>!#	13.37 (1.04) *<100>!#	14.65 (1.19) *<100>!#	585.71 (146.09) *<100>!#	286.94 (54.9) *<100>!#	118.51 (13.22) *<100>!#	97.39 (10.68) *<100>!#	110.76 (12.52) *<100>!#	2460.45 (967.12) *<100>!#	1309.05 (321.77) *<100>!#	671 (110.97) *<100>!#	555.82 (87.97) *<100>!#	646.91 (103.59) *<100>!#
mde_mi7_1	NA	3.68 (2.92) *<33>	1.28 (0.11) *<79>	1.04 (0.05) *<9>	1 (0.05) *<12>	12.55 (5.09) *<100>	6.09 (1.15) *<100>!#	2.31 (0.15) *<100>!#	1.81 (0.08) *<100>!#	1.75 (0.07) *<100>!#	55.47 (9.56) *<100>!#	26.44 (2.9) *<100>!#	10.12 (0.66) *<100>!#	7.62 (0.35) *<100>!#	7.91 (0.43) *<100>!#	287.38 (46.37) *<100>!#	143 (16.4) *<100>!#	58.3 (4.35) *<100>!#	46.16 (3.12) *<100>!#	51.74 (3.17) *<100>!#	1104.8 (245.52) *<100>!#	608.32 (105.21) *<100>!#	298.02 (32.98) *<100>!#	251.49 (24.35) *<100>!#	281.14 (28.16) *<100>!#
mde_mi7_2	NA	3.31 (2.09) *<28>	1.29 (0.1) *<90>	1.03 (0.04) *<5>	1 (0.04) *<3>	13.67 (4.96) *<100>	6.23 (1.42) *<100>!#	2.33 (0.16) *<100>!#	1.81 (0.08) *<100>!#	1.75 (0.08) *<100>!#	54.12 (9.56) *<100>!#	27.02 (2.89) *<100>!#	9.92 (0.52) *<100>!#	7.68 (0.45) *<100>!#	7.93 (0.4) *<100>!#	294.94 (50.2) *<100>!#	142.37 (17.24) *<100>!#	57.42 (3.98) *<100>!#	46.68 (2.63) *<100>!#	51.8 (3.91) *<100>!#	1095.48 (249.5) *<100>!#	602.28 (92.98) *<100>!#	298.76 (29.91) *<100>!#	250.68 (25.69) *<100>!#	282.48 (26.66) *<100>!#
mde_mi7_bias	NA	NA	0.85 (0.11) *<30>	0.98 (0.05) *<7>	1 (0.04) *<6>	0.05 (0.04) *<96>	0.11 (0.03) *<100>!#	0.36 (0.03) *<100>!#	0.5 (0.02) *<100>!#	0.54 (0.02) *<100>!#	0.01 (0) *<100>!#	0.03 (0) *<100>!#	0.08 (0.01) *<100>!#	0.11 (0.01) *<100>!#	0.11 (0.01) *<100>!#	0 (0) *<100>!#	0.01 (0) *<100>!#	0.01 (0) *<100>!#	0.02 (0) *<100>!#	0.02 (0) *<100>!#	0 (0) *<100>!#	0 (0) *<100>!#	0 (0) *<100>!#	0 (0) *<100>!#	0 (0) *<100>!#
mde_mi8	NA	6.53 (7.25) *<60>	1.47 (0.12) *<98>	1.07 (0.06) *<28>	1.01 (0.08) *<9>	18.89 (9.11) *<100>!#	8.65 (1.73) *<100>!#	2.99 (0.23) *<100>!#	2.28 (0.13) *<100>!#	2.25 (0.15) *<100>!#	94.38 (19.67) *<100>!#	44.89 (6.41) *<100>!#	17.02 (1.46) *<100>!#	13.54 (1.01) *<100>!#	14.41 (1.14) *<100>!#	581.25 (139.91) *<100>!#	286.09 (49.5) *<100>!#	118.6 (13.82) *<100>!#	95.78 (10.53) *<100>!#	110.77 (10.85) *<100>!#	2389.16 (1259.87) *<100>!#	1369.9 (381.11) *<100>!#	656.31 (107.92) *<100>!#	556.24 (91.17) *<100>!#	662.91 (137.79) *<100>!#
mde_mi8_1	NA	4.46 (3.67) *<38>	1.29 (0.1) *<83>	1.04 (0.05) *<17>	1 (0.05) *<7>	13.45 (5.03) *<100>!@	6.31 (1.23) *<100>!#	2.31 (0.18) *<100>!#	1.82 (0.09) *<100>!#	1.73 (0.07) *<100>!#	54.26 (9.39) *<100>!#	26.6 (2.77) *<100>!#	10.05 (0.63) *<100>!#	7.68 (0.35) *<100>!#	7.85 (0.38) *<100>!#	286.35 (47.27) *<100>!#	141.87 (15.88) *<100>!#	57.92 (4.26) *<100>!#	45.95 (3.42) *<100>!#	51.98 (3.24) *<100>!#	1069.45 (304.54) *<100>!#	608.7 (106.3) *<100>!#	298.64 (30.38) *<100>!#	253.19 (22.74) *<100>!#	286.3 (27.62) *<100>!#
mde_mi8_2	NA	3.65 (3.05) *<34>	1.28 (0.11) *<80>	1.03 (0.05) *<12>	1.01 (0.05) *<7>	12.19 (4.41) *<100>!@	6.31 (1.2) *<100>!#	2.35 (0.16) *<100>!#	1.8 (0.07) *<100>!#	1.74 (0.08) *<100>!#	54.7 (8.67) *<100>!#	26.67 (3.04) *<100>!#	10.06 (0.68) *<100>!#	7.71 (0.41) *<100>!#	7.82 (0.39) *<100>!#	292.91 (51.26) *<100>!#	144.06 (16.52) *<100>!#	58.29 (4.28) *<100>!#	46.69 (3.35) *<100>!#	51.8 (3.62) *<100>!#	1062.14 (302.96) *<100>!#	616.91 (102.05) *<100>!#	296.88 (28.42) *<100>!#	248.8 (22.76) *<100>!#	283.56 (29.11) *<100>!#
mde_mi8_bias	NA	NA	0.85 (0.11) *<26>	0.97 (0.06) *<11>	0.99 (0.04) *<6>	0.06 (0.04) *<96>	0.1 (0.02) *<100>!#	0.36 (0.03) *<100>!#	0.5 (0.02) *<100>!#	0.54 (0.02) *<100>!#	0.01 (0) *<100>!#	0.03 (0) *<100>!#	0.08 (0.01) *<100>!#	0.11 (0.01) *<100>!#	0.11 (0.01) *<100>!#	0 (0) *<100>!#	0.01 (0) *<100>!#	0.01 (0) *<100>!#	0.02 (0) *<100>!#	0.02 (0) *<100>!#	0 (0) *<100>!#	0 (0) *<100>!#	0 (0) *<100>!#	0 (0) *<100>!#	0 (0) *<100>!#
mde_mi9	NA	4.45 (3.55) *<42>	1.35 (0.11) *<95>	1.05 (0.04) *<17>	1.01 (0.04) *<4>	14.77 (4.98) *<100>!#	6.39 (1.14) *<100>!#	2.35 (0.16) *<100>!#	1.83 (0.09) *<100>!#	1.75 (0.07) *<100>!#	55.12 (9.57) *<100>!#	27.4 (3.35) *<100>!#	10.05 (0.65) *<100>!#	7.74 (0.38) *<100>!#	7.86 (0.44) *<100>!#	287.19 (53.5) *<100>!#	144.73 (16.37) *<100>!#	58.41 (3.91) *<100>!#	46.15 (3.42) *<100>!#	51.92 (3.38) *<100>!#	1108.8 (254.78) *<100>!#	613.72 (102.07) *<100>!#	297.54 (29.41) *<100>!#	251.14 (24.34) *<100>!#	279.49 (25.83) *<100>!#
mde_bias1	NA	NA	NA	NA	NA	NA	NA	NA	NA	NA	NA	NA	NA	NA	NA	NA	NA	NA	NA	NA	NA	NA	NA	NA	NA
mde_bias2	NA	NA	NA	NA	NA	NA	NA	NA	NA	NA	NA	NA	NA	NA	NA	NA	NA	NA	NA	NA	NA	NA	NA	NA	NA
mde_bias	NA	NA	NA	NA	NA	NA	NA	NA	NA	NA	NA	NA	NA	NA	NA	NA	NA	NA	NA	NA	NA	NA	NA	NA	NA

**Table 7:** Odds ratios between major depressive episodes and the input symptoms of dysthymic disorder by assumed symptom prevalence and correlations NA = not applicable due to 0 case in the denominators for the calculation of odds ratios, except for dys_mi_bias that is a continuous variable Mean (standard deviation) *<number> = the numbers of significant statistics (p < 0.05) in 100 simulations !# = p < 0.001 in all of the 100 simulations !@ = p < 0.001 in some of the 100 simulations Note: See Table 1 for variable definitions and Table 2 for the prevalence of major depressive episodes, dysthymic disorder, and manic episodes. dys = Dysthymic Disorder; dys_ma = Depressed mood most of the day for more days than not, for at least 2 years; dys_mi = Minor criteria (at least 2 items); dys_mi1 = Poor appetite or overeating; dys_mi1_1 = Poor appetite; dys_mi1_2 = Overeating; dys_mi1_bias = Information of the domain not explained by the input variables; dys_mi4 = Low self-esteem; dys_mi6 = Feelings of hopelessness; dys_mi_bias = Information of minor criteria not explained by input variables; dys_bias = Information of diagnosis not explained by major or minor criteria

Assumed symptom prevalence	0.05	0.1	0.3	0.5	0.7	0.05	0.1	0.3	0.5	0.7	0.05	0.1	0.3	0.5	0.7	0.05	0.1	0.3	0.5	0.7	0.05	0.1	0.3	0.5	0.7
Assumed symptom correlations	0	0	0	0	0	0.1	0.1	0.1	0.1	0.1	0.4	0.4	0.4	0.4	0.4	0.7	0.7	0.7	0.7	0.7	0.9	0.9	0.9	0.9	0.9
dys	NA	2.48 (3.75) *<8>	1.11 (0.12) *<27>	1 (0.05) *<7>	1 (0.04) *<3>	27 (10.7) *<100>!@	10.07 (1.97) *<100>!#	2.65 (0.18) *<100>!#	1.88 (0.08) *<100>!#	1.76 (0.07) *<100>!#	78.67 (13.07) *<100>!#	36.46 (4.02) *<100>!#	12.25 (0.82) *<100>!#	8.72 (0.39) *<100>!#	8.55 (0.46) *<100>!#	356.91 (53.77) *<100>!#	177.77 (21.1) *<100>!#	68.38 (4.77) *<100>!#	53.02 (3.61) *<100>!#	58.16 (3.96) *<100>!#	1227.02 (279.43) *<100>!#	714.1 (104.4) *<100>!#	340.29 (34.83) *<100>!#	285.01 (25.67) *<100>!#	320.39 (31.92) *<100>!#
dys_ma	NA	1.11 (1.04) *<2>	0.99 (0.1) *<9>	1 (0.05) *<8>	1 (0.04) *<3>	10.36 (3.88) *<100>	4.98 (0.93) *<100>!#	2.11 (0.14) *<100>!#	1.74 (0.08) *<100>!#	1.73 (0.07) *<100>!#	51.78 (8.02) *<100>!#	25.41 (2.85) *<100>!#	9.74 (0.61) *<100>!#	7.49 (0.34) *<100>!#	7.74 (0.42) *<100>!#	280.71 (39.7) *<100>!#	142.28 (15.82) *<100>!#	56.82 (3.94) *<100>!#	45.36 (3.19) *<100>!#	50.97 (3.39) *<100>!#	1012.97 (224.02) *<100>!#	599.55 (84.39) *<100>!#	290.76 (29.9) *<100>!#	246.11 (22.12) *<100>!#	278.11 (27.45) *<100>!#
dys_mi	NA	NA	1.89 (0.26) *<100>!@	1.34 (0.3) *<26>	NA	NA	32.86 (12.62) *<100>!#	9.29 (1.4) *<100>!#	7.98 (1.73) *<100>!#	9.98 (5.98) *<100>!#	NA	336.98 (230.36) *<100>!#	121.52 (33.61) *<100>!#	111.54 (37.55) *<100>!#	132.69 (43.23) *<100>!#	NA	NA	1266.74 (844.11) *<100>!#	1094.52 (693.04) *<100>!#	1396.27 (848.02) *<100>!#	NA	NA	NA	NA	NA
dys_mi1	NA	1.16 (0.79) *<2>	1 (0.07) *<4>	1 (0.05) *<5>	0.99 (0.07) *<4>	12.17 (4.89) *<100>!#	6.04 (1.16) *<100>!#	2.46 (0.18) *<100>!#	2.09 (0.11) *<100>!#	2.23 (0.17) *<100>!#	81.03 (17.26) *<100>!#	40.34 (6.35) *<100>!#	15.17 (1.08) *<100>!#	12.42 (0.79) *<100>!#	13.89 (1.03) *<100>!#	542.69 (151.88) *<100>!#	256.7 (45.35) *<100>!#	108.49 (11.47) *<100>!#	89.25 (8.69) *<100>!#	105.56 (11.06) *<100>!#	2231.04 (811.46) *<100>!#	1227.09 (337.24) *<100>!#	629.17 (93.69) *<100>!#	514.39 (79.49) *<100>!#	603.69 (91.62) *<100>!#
dys_mi1_1	NA	1.05 (1.01) *<1>	1 (0.09) *<6>	1 (0.05) *<4>	1 (0.04) *<4>	10.35 (3.78) *<100>!@	5.18 (1.08) *<100>!#	2.11 (0.14) *<100>!#	1.76 (0.08) *<100>!#	1.74 (0.07) *<100>!#	51.94 (8.92) *<100>!#	25.44 (3.13) *<100>!#	9.65 (0.6) *<100>!#	7.55 (0.38) *<100>!#	7.83 (0.41) *<100>!#	280.79 (43.37) *<100>!#	139.81 (16.44) *<100>!#	56.56 (4.41) *<100>!#	45.84 (3.03) *<100>!#	51.63 (3.76) *<100>!#	1061.63 (233.17) *<100>!#	595.36 (95.11) *<100>!#	296.28 (31.39) *<100>!#	243.2 (22.49) *<100>!#	278.09 (25.88) *<100>!#
dys_mi1_2	NA	1.28 (1.07) *<2>	1 (0.07) *<2>	1 (0.05) *<9>	1 (0.05) *<8>	9.67 (3.94) *<100>	4.96 (0.97) *<100>!#	2.08 (0.13) *<100>!#	1.76 (0.08) *<100>!#	1.73 (0.08) *<100>!#	51.24 (8.37) *<100>!#	25.87 (2.78) *<100>!#	9.71 (0.56) *<100>!#	7.54 (0.37) *<100>!#	7.76 (0.4) *<100>!#	277.41 (42.55) *<100>!#	139.09 (15.72) *<100>!#	56.83 (4.09) *<100>!#	45.25 (2.9) *<100>!#	50.95 (3.29) *<100>!#	1048.81 (237.28) *<100>!#	598.57 (113.61) *<100>!#	294.79 (26) *<100>!#	244.96 (22.64) *<100>!#	279.28 (27.78) *<100>!#
dys_mi1_bias	NA	NA	1.02 (0.13) *<3>	1 (0.06) *<6>	0.99 (0.04) *<7>	NA	0.12 (0.03) *<100>!@	0.38 (0.03) *<100>!#	0.5 (0.02) *<100>!#	0.54 (0.02) *<100>!#	0.01 (0) *<100>!#	0.03 (0) *<100>!#	0.08 (0) *<100>!#	0.11 (0.01) *<100>!#	0.11 (0) *<100>!#	0 (0) *<100>!#	0.01 (0) *<100>!#	0.02 (0) *<100>!#	0.02 (0) *<100>!#	0.02 (0) *<100>!#	0 (0) *<100>!#	0 (0) *<100>!#	0 (0) *<100>!#	0 (0) *<100>!#	0 (0) *<100>!#
dys_mi4	NA	1.14 (1.1) *<1>	1 (0.08) *<5>	1 (0.05) *<7>	1 (0.04) *<4>	10.63 (4.7) *<100>	4.91 (0.9) *<100>!#	2.09 (0.18) *<100>!#	1.75 (0.09) *<100>!#	1.73 (0.06) *<100>!#	49.85 (7.44) *<100>!#	25.46 (3.09) *<100>!#	9.73 (0.59) *<100>!#	7.52 (0.34) *<100>!#	7.75 (0.41) *<100>!#	285.02 (52.61) *<100>!#	140.69 (17.48) *<100>!#	56.47 (3.91) *<100>!#	45.67 (2.84) *<100>!#	51.33 (3.38) *<100>!#	1078.69 (255.85) *<100>!#	603.36 (98.29) *<100>!#	287.72 (30.29) *<100>!#	245.89 (21.14) *<100>!#	276.99 (26.99) *<100>!#
dys_mi6	NA	1.1 (1.08) *<3>	1.01 (0.09) *<4>	1 (0.04) *<6>	1.01 (0.04) *<2>	9.96 (3.89) *<99>	4.87 (1) *<100>!#	2.09 (0.15) *<100>!#	1.75 (0.08) *<100>!#	1.73 (0.06) *<100>!#	50.49 (8.29) *<100>!#	25.69 (2.93) *<100>!#	9.74 (0.54) *<100>!#	7.5 (0.36) *<100>!#	7.72 (0.4) *<100>!#	282.15 (48.38) *<100>!#	139.82 (16.34) *<100>!#	56.84 (3.95) *<100>!#	45.87 (3.19) *<100>!#	51.29 (3.8) *<100>!#	1095.48 (261.71) *<100>!#	599.47 (101.65) *<100>!#	292.4 (26.04) *<100>!#	244.12 (20.86) *<100>!#	279.26 (28.9) *<100>!#
dys_mi_bias	NA	NA	NA	NA	NA	NA	NA	NA	NA	NA	NA	NA	NA	NA	NA	NA	NA	NA	NA	NA	NA	NA	NA	NA	NA
dys_bias	NA	0.28 (0.26) *<70>	0.55 (0.09) *<99>	0.78 (0.26) *<21>	NA	0.02 (0.01) *<100>!#	0.03 (0.01) *<100>!#	0.1 (0.02) *<100>!#	0.11 (0.03) *<100>!#	0.1 (0.05) *<100>!#	0 (0) *<100>!#	0 (0) *<100>!#	0.01 (0) *<100>!#	0.01 (0) *<100>!#	0.01 (0) *<100>!#	0 (0) *<100>!#	0 (0) *<100>!#	0 (0) *<100>!#	0 (0) *<100>!#	0 (0) *<100>!#	0 (0) *<100>!#	0 (0) *<100>!#	0 (0) *<100>!#	0 (0) *<100>!#	0 (0) *<100>!#

**Table 8:** Odds ratios between major depressive episodes and the input symptoms of manic episodes by assumed symptom prevalence and correlations NA = not applicable due to 0 case in the denominators for the calculation of odds ratios Mean (standard deviation) *<number> = the numbers of significant statistics (p < 0.05) in 100 simulations !# = p < 0.001 in all of the 100 simulations !@ = p < 0.001 in some of the 100 simulations manic = Manic episodes; man_ma1 = Elevated mood; man_ma2 = Expansive mood; man_ma3 = Irritable mood; man_mi1 = Increased self-esteem or grandiosity; man_mi1_1 = Increased self-esteem; man_mi1_2 = Grandiosity; man_mi1_bias = Information of the domain not explained by the input variables; man_mi2 = Decreased need for sleep (e.g., feels rested after only 3 hours of sleep); man_mi3 = More talkative than usual or pressure to keep talking; man_mi3_1 = More talkative than usual; man_mi3_2 = Pressure to keep talking; man_mi3_bias = Information of the domain not explained by the input variables; man_mi4 = Flight of ideas or subjective experience that thoughts are racing; man_mi4_1 = Flight of ideas; man_mi4_2 = Subjective experience that thoughts are racing; man_mi4_bias = Information of the domain not explained by the input variables; man_mi5 = Distractibility (i.e., attention too easily drawn to unimportant or irrelevant external stimuli); man_mi6 = Increase in goal-directed activity (either socially, at work or school, or sexually) or psychomotor agitation; man_mi6_1 = Increase in goal-directed activity ; man_mi6_2 = Psychomotor agitation; man_mi6_bias = Information of the domain not explained by the input variables; man_mi7 = Excessive involvement in pleasurable activities that have a high potential for painful consequences (e.g., engaging in unrestrained buying sprees, sexual indiscretions, or foolish business investments); man_bias1 = Information of diagnosis due to top-censoring for choosing at least three symptoms; man_bias2 = Information of diagnosis due to top-censoring for choosing at least four symptoms; man_bias = Information of diagnosis not explained by symptoms

Assumed symptom prevalence	0.05	0.1	0.3	0.5	0.7	0.05	0.1	0.3	0.5	0.7	0.05	0.1	0.3	0.5	0.7	0.05	0.1	0.3	0.5	0.7	0.05	0.1	0.3	0.5	0.7
Assumed symptom correlations	0	0	0	0	0	0.1	0.1	0.1	0.1	0.1	0.4	0.4	0.4	0.4	0.4	0.7	0.7	0.7	0.7	0.7	0.9	0.9	0.9	0.9	0.9
manic	0 (0)	0.38 (3.77) *<1>	1.01 (0.13) *<9>	1 (0.04) *<4>	1.01 (0.05) *<8>	0.78 (1.9) *<3>	0.96 (0.67) *<2>	1 (0.09) *<6>	1.01 (0.05) *<7>	1 (0.04) *<5>	1.02 (0.42) *<4>	1.01 (0.23) *<10>	1.01 (0.06) *<4>	1 (0.05) *<5>	1 (0.04) *<6>	0.99 (0.27) *<4>	0.98 (0.12) *<3>	0.99 (0.05) *<7>	1 (0.04) *<5>	1 (0.04) *<4>	0.97 (0.24) *<5>	1.02 (0.13) *<7>	1 (0.05) *<3>	1 (0.04)	1.01 (0.04) *<4>
man_ma1	NA	1.07 (0.98)	1 (0.08) *<2>	1 (0.04) *<3>	1 (0.04) *<6>	1.02 (0.82) *<1>	1 (0.35) *<4>	1.01 (0.07) *<7>	1 (0.05) *<7>	1 (0.04) *<5>	1.01 (0.38) *<9>	1.01 (0.17) *<7>	1 (0.06) *<4>	1 (0.04) *<7>	1.01 (0.05) *<8>	0.97 (0.21) *<2>	0.99 (0.12) *<4>	1 (0.06) *<8>	1 (0.04) *<9>	1 (0.05) *<5>	0.97 (0.23) *<7>	1.02 (0.13) *<6>	1 (0.05) *<3>	0.99 (0.04) *<1>	1.01 (0.05) *<3>
man_ma2	NA	0.96 (1.07) *<1>	0.99 (0.08) *<5>	1 (0.06) *<12>	1 (0.04) *<6>	0.96 (0.77) *<2>	1.02 (0.35) *<7>	1 (0.08) *<8>	1.01 (0.05) *<10>	1 (0.04) *<5>	1.01 (0.32) *<3>	1.01 (0.15) *<3>	1.01 (0.06) *<2>	1 (0.05) *<8>	0.99 (0.05) *<6>	0.97 (0.21) *<2>	0.99 (0.12) *<5>	0.99 (0.06) *<9>	1 (0.04) *<4>	1 (0.05) *<4>	0.98 (0.25) *<6>	1.02 (0.12) *<8>	1 (0.05) *<2>	0.99 (0.04) *<2>	1 (0.05) *<5>
man_ma3	NA	1.05 (1.07) *<1>	1 (0.09) *<2>	1 (0.04) *<3>	1.01 (0.05) *<7>	0.93 (0.76) *<3>	0.97 (0.31) *<8>	1 (0.08) *<6>	1.01 (0.05) *<5>	1 (0.04) *<6>	1 (0.27) *<1>	1 (0.17) *<7>	1.01 (0.06) *<4>	1 (0.05) *<7>	1 (0.04) *<2>	0.98 (0.25) *<6>	0.99 (0.12) *<3>	1 (0.05) *<7>	1 (0.04) *<6>	1 (0.04) *<1>	0.98 (0.23) *<4>	1.02 (0.13) *<8>	1 (0.05) *<3>	1 (0.04) *<1>	1.01 (0.05) *<3>
man_mi1	NA	1.14 (0.94) *<2>	1 (0.07) *<3>	1.01 (0.06) *<8>	1.01 (0.07) *<5>	1.09 (0.71) *<8>	1.03 (0.27) *<6>	1.01 (0.07) *<4>	1 (0.05) *<3>	1.01 (0.06) *<2>	1.05 (0.27) *<5>	1.02 (0.14) *<5>	1.02 (0.05) *<6>	1 (0.04) *<5>	1 (0.06) *<8>	0.99 (0.22) *<6>	0.99 (0.11) *<4>	1 (0.06) *<9>	1 (0.04) *<3>	1 (0.05) *<6>	0.97 (0.23) *<8>	1.02 (0.12) *<6>	1 (0.05) *<3>	1 (0.04) *<1>	1 (0.05) *<3>
man_mi1_1	NA	1.26 (1.12) *<1>	1 (0.08) *<7>	1.01 (0.05) *<9>	1.01 (0.05) *<6>	1.1 (0.82) *<3>	1.02 (0.34) *<6>	1 (0.09) *<9>	1 (0.05) *<7>	1.01 (0.05) *<6>	1.07 (0.36) *<5>	1.01 (0.17) *<8>	1.02 (0.06) *<6>	0.99 (0.04) *<4>	1 (0.04) *<7>	0.97 (0.22) *<2>	0.98 (0.13) *<6>	1 (0.05) *<8>	1 (0.04) *<5>	1 (0.05) *<3>	0.97 (0.23) *<4>	1.02 (0.13) *<6>	1 (0.05) *<3>	0.99 (0.03) *<1>	1 (0.05) *<2>
man_mi1_2	NA	1 (1.03) *<2>	0.99 (0.09) *<4>	1.01 (0.05) *<6>	1 (0.05) *<4>	1.1 (0.96) *<7>	1.04 (0.33) *<6>	1.02 (0.07) *<3>	1 (0.05) *<6>	1.01 (0.04) *<5>	1.06 (0.37) *<5>	1.01 (0.19) *<6>	1.01 (0.05) *<1>	1 (0.04) *<4>	1 (0.05) *<8>	1 (0.27) *<7>	1 (0.12) *<3>	1 (0.06) *<5>	1 (0.04) *<5>	1.01 (0.05) *<5>	0.96 (0.25) *<7>	1.01 (0.13) *<9>	1 (0.04) *<1>	1 (0.03)	1.01 (0.04) *<3>
man_mi1_bias	NA	NA	1.02 (0.15) *<5>	1 (0.05) *<4>	1 (0.04) *<6>	NA	NA	0.99 (0.12) *<4>	1 (0.05) *<2>	1 (0.04) *<4>	NA	1.09 (0.32) *<9>	0.99 (0.06) *<3>	1 (0.04) *<4>	1 (0.04) *<3>	1.13 (0.39) *<5>	1.04 (0.15) *<3>	1 (0.06) *<6>	1 (0.04) *<7>	1 (0.05) *<4>	1.14 (0.34) *<6>	1 (0.15) *<9>	1 (0.05) *<4>	1.01 (0.03)	1 (0.04) *<2>
man_mi2	NA	1.07 (1.12) *<2>	1.01 (0.08) *<4>	1 (0.04) *<3>	1 (0.04) *<3>	0.94 (0.8) *<2>	1.01 (0.29) *<4>	0.99 (0.07) *<4>	1.01 (0.05) *<6>	1.01 (0.05) *<9>	1.01 (0.36) *<7>	1.02 (0.17) *<6>	1 (0.06) *<8>	1 (0.04) *<4>	1 (0.05) *<8>	0.99 (0.25) *<3>	0.99 (0.13) *<5>	1 (0.06) *<10>	1 (0.04) *<5>	1 (0.04) *<4>	0.97 (0.23) *<7>	1.02 (0.13) *<9>	1 (0.05) *<3>	1 (0.04) *<2>	1.01 (0.05) *<3>
man_mi3	NA	1.25 (1.13) *<5>	1 (0.09) *<8>	1 (0.05) *<4>	1.01 (0.06) *<5>	0.99 (0.59) *<1>	1.01 (0.25) *<3>	1 (0.08) *<7>	1 (0.05) *<5>	1 (0.06) *<4>	1.06 (0.3) *<7>	1.01 (0.16) *<8>	1.01 (0.06) *<3>	1 (0.05) *<9>	1 (0.06) *<8>	0.97 (0.2) *<2>	0.99 (0.11) *<6>	1 (0.05) *<6>	1 (0.04) *<5>	1 (0.05) *<4>	0.97 (0.21) *<6>	1.01 (0.11) *<4>	1 (0.04) *<4>	1 (0.04) *<1>	1.01 (0.04) *<3>
man_mi3_1	NA	1.3 (1.39) *<3>	0.99 (0.09) *<6>	1 (0.05) *<6>	1 (0.04) *<5>	1 (0.77) *<1>	0.97 (0.32) *<5>	0.99 (0.08) *<10>	1 (0.04) *<6>	1.01 (0.05) *<6>	1.03 (0.36) *<7>	1.01 (0.18) *<8>	1.01 (0.06) *<6>	1 (0.05) *<7>	1 (0.04) *<3>	0.96 (0.2) *<1>	0.98 (0.12) *<3>	1 (0.05) *<9>	1 (0.04) *<4>	1 (0.04) *<5>	0.97 (0.22) *<7>	1.01 (0.12) *<6>	1 (0.05) *<3>	1 (0.04) *<2>	1.01 (0.04) *<1>
man_mi3_2	NA	1.12 (1.24) *<3>	1 (0.1) *<7>	1 (0.05) *<8>	1 (0.04) *<2>	1 (0.77) *<2>	1.04 (0.34) *<5>	1 (0.08) *<6>	1 (0.05) *<8>	1 (0.04) *<4>	1.06 (0.37) *<6>	1 (0.18) *<8>	1.01 (0.06) *<7>	1 (0.05) *<5>	1 (0.05) *<6>	0.99 (0.25) *<6>	1 (0.13) *<4>	1 (0.05) *<1>	1 (0.04) *<8>	1 (0.04) *<5>	0.96 (0.24) *<8>	1.02 (0.12) *<7>	1 (0.05) *<2>	1 (0.03)	1 (0.04) *<3>
man_mi3_bias	NA	NA	1.02 (0.15) *<6>	1 (0.05) *<5>	1 (0.04) *<4>	NA	NA	1.03 (0.13) *<10>	1.01 (0.05) *<4>	1 (0.04) *<5>	NA	1.08 (0.3) *<6>	0.99 (0.08) *<8>	1 (0.05) *<7>	1 (0.04) *<5>	1.1 (0.29) *<1>	1.03 (0.16) *<3>	1 (0.06) *<7>	1 (0.04) *<6>	1 (0.04) *<4>	1.13 (0.34) *<8>	0.99 (0.14) *<10>	1 (0.05) *<5>	1.01 (0.03) *<1>	1 (0.04) *<1>
man_mi4	NA	0.93 (0.67)	1 (0.08) *<5>	1.01 (0.05) *<1>	1 (0.08) *<5>	1 (0.51) *<3>	1.02 (0.27) *<7>	1.01 (0.07) *<7>	1 (0.05) *<4>	1 (0.07) *<7>	1.02 (0.3) *<9>	1.02 (0.15) *<9>	1.01 (0.05) *<2>	1 (0.04) *<3>	1.01 (0.05) *<5>	0.98 (0.22) *<5>	0.99 (0.11) *<4>	1 (0.05) *<4>	1 (0.04) *<4>	1 (0.05) *<3>	0.98 (0.21) *<6>	1.02 (0.11) *<5>	1 (0.04) *<5>	1 (0.04) *<3>	1 (0.05) *<5>
man_mi4_1	NA	0.87 (0.85) *<1>	0.99 (0.09) *<7>	1 (0.04) *<4>	1 (0.05) *<8>	0.98 (0.7)	1 (0.3) *<3>	1 (0.07) *<5>	1.01 (0.04) *<4>	1 (0.04) *<6>	1.05 (0.4) *<8>	1.02 (0.18) *<4>	1.01 (0.06) *<4>	1 (0.04) *<3>	1 (0.04) *<5>	0.98 (0.24) *<4>	0.99 (0.11) *<2>	1 (0.05) *<3>	1 (0.04) *<7>	1 (0.04) *<4>	0.97 (0.23) *<4>	1.02 (0.12) *<8>	1 (0.05) *<5>	1 (0.03) *<2>	1 (0.05) *<4>
man_mi4_2	NA	1.03 (1.1) *<3>	1 (0.09) *<4>	1.01 (0.04) *<4>	1 (0.05) *<7>	1.02 (0.8) *<2>	1.05 (0.34) *<6>	1 (0.08) *<4>	1 (0.05) *<6>	0.99 (0.05) *<8>	1.01 (0.38) *<10>	1.02 (0.18) *<6>	1.01 (0.05) *<3>	1 (0.04) *<3>	1.01 (0.04) *<4>	0.97 (0.26) *<5>	1 (0.13) *<7>	1 (0.05) *<2>	1 (0.04) *<4>	1 (0.05) *<4>	0.97 (0.24) *<6>	1.02 (0.12) *<7>	1 (0.05) *<3>	0.99 (0.04) *<2>	1.01 (0.05) *<4>
man_mi4_bias	NA	NA	1.05 (0.15) *<5>	0.99 (0.06) *<7>	1 (0.04) *<3>	NA	NA	1.02 (0.09) *<1>	0.99 (0.05) *<5>	1 (0.04) *<3>	NA	1.04 (0.26) *<2>	1 (0.07) *<4>	1 (0.05) *<4>	1 (0.04) *<4>	1.15 (0.44) *<7>	1.02 (0.15) *<3>	1.01 (0.05) *<4>	1 (0.04) *<9>	1 (0.04) *<4>	1.13 (0.37) *<9>	1 (0.14) *<8>	1 (0.05) *<3>	1.01 (0.03)	1 (0.04) *<4>
man_mi5	NA	1.2 (1.17) *<2>	1.01 (0.09) *<1>	1 (0.04) *<2>	0.99 (0.04) *<4>	0.9 (0.7)	0.97 (0.34) *<6>	0.99 (0.07) *<6>	1 (0.04) *<6>	1 (0.04) *<2>	0.99 (0.36) *<6>	1.01 (0.17) *<6>	1.01 (0.06) *<4>	1 (0.04) *<5>	1 (0.04) *<6>	0.96 (0.24) *<4>	0.99 (0.12) *<5>	1 (0.05) *<6>	1 (0.04) *<4>	1 (0.05) *<5>	0.97 (0.24) *<5>	1.02 (0.13) *<6>	1 (0.05) *<3>	0.99 (0.04) *<2>	1 (0.04) *<3>
man_mi6	NA	1.09 (0.82) *<2>	1.01 (0.09) *<7>	1 (0.06) *<7>	0.99 (0.06) *<1>	1.08 (0.7) *<8>	1.03 (0.24) *<4>	0.99 (0.06) *<3>	1 (0.05) *<6>	1.01 (0.06) *<3>	1.04 (0.28) *<6>	1.02 (0.15) *<6>	1.02 (0.06) *<9>	1 (0.04) *<6>	1 (0.05) *<6>	1.01 (0.21) *<4>	0.99 (0.1) *<4>	1 (0.05) *<6>	1.01 (0.04) *<7>	1 (0.05) *<2>	0.99 (0.22) *<6>	1.01 (0.12) *<6>	1 (0.05) *<6>	1 (0.04) *<2>	1.01 (0.05) *<5>
man_mi6_1	NA	1 (0.91) *<1>	1.01 (0.09) *<4>	1 (0.05) *<7>	0.99 (0.04) *<3>	1.06 (0.91) *<6>	1.06 (0.34) *<7>	1 (0.08) *<5>	1 (0.04) *<2>	1 (0.04) *<5>	0.98 (0.35) *<6>	1.02 (0.17) *<4>	1.02 (0.06) *<5>	1 (0.04) *<5>	1 (0.04) *<5>	0.99 (0.25) *<4>	0.99 (0.12) *<2>	1 (0.05) *<5>	1 (0.04) *<5>	1 (0.04) *<5>	0.97 (0.24) *<5>	1.01 (0.13) *<11>	1 (0.05) *<5>	1 (0.03)	1.01 (0.05) *<6>
man_mi6_2	NA	1.18 (1.29) *<4>	1 (0.08) *<4>	1.01 (0.05) *<7>	0.99 (0.04) *<5>	1.06 (0.85) *<3>	1 (0.32) *<6>	0.98 (0.08) *<8>	1 (0.04) *<2>	1 (0.04) *<3>	1.06 (0.35) *<3>	1.01 (0.18) *<9>	1.02 (0.06) *<5>	1 (0.04) *<4>	1 (0.05) *<5>	1.01 (0.24) *<4>	0.99 (0.12) *<4>	1 (0.06) *<7>	1 (0.04) *<7>	1 (0.04) *<2>	0.97 (0.24) *<7>	1.02 (0.12) *<7>	1 (0.05) *<3>	0.99 (0.04)	1.01 (0.04) *<3>
man_mi6_bias	NA	NA	1.01 (0.14) *<5>	0.99 (0.06) *<7>	1.01 (0.04) *<8>	NA	NA	1.03 (0.14) *<8>	1 (0.05) *<4>	1.01 (0.04) *<5>	NA	1.05 (0.29) *<7>	0.99 (0.06) *<4>	1.01 (0.04) *<5>	1 (0.04) *<8>	1.12 (0.41) *<4>	1.03 (0.15) *<5>	1 (0.06) *<7>	1 (0.05) *<8>	1 (0.04) *<4>	1.15 (0.37) *<8>	1 (0.14) *<8>	1 (0.05) *<2>	1.01 (0.03) *<1>	1 (0.04) *<2>
man_mi7	NA	1.27 (1.22) *<1>	1 (0.1) *<8>	1 (0.05) *<6>	1.01 (0.04) *<1>	0.99 (0.89) *<5>	1 (0.31) *<4>	1 (0.08) *<4>	1 (0.05) *<4>	1 (0.04) *<5>	1.07 (0.36) *<6>	1 (0.17) *<7>	1.01 (0.05) *<4>	1 (0.04) *<6>	1.01 (0.04) *<4>	0.99 (0.24) *<4>	0.98 (0.11) *<2>	1 (0.05) *<6>	1 (0.04) *<4>	1.01 (0.05) *<5>	0.97 (0.24) *<9>	1.02 (0.12) *<6>	1 (0.05) *<5>	1 (0.04) *<4>	1 (0.04) *<4>
man_bias1	NA	NA	NA	NA	NA	NA	NA	NA	NA	NA	NA	NA	NA	NA	NA	NA	NA	NA	NA	NA	NA	NA	NA	NA	NA
man_bias2	NA	NA	NA	NA	NA	NA	NA	NA	NA	NA	NA	NA	NA	NA	NA	NA	NA	NA	NA	NA	NA	NA	NA	NA	NA
man_bias	NA	NA	NA	NA	NA	NA	NA	NA	NA	NA	NA	NA	NA	NA	NA	NA	NA	NA	NA	NA	NA	NA	NA	NA	NA

In Table 6, the odds ratios of developing two symptoms in the major criteria due to the diagnosis of major depressive episodes (mde_ma1 and mde_ma2) were not available for their frequent co-occurrence with the diagnosis. The occurrence of symptoms and major depressive episodes was not sufficient to calculate odds ratios, assuming 0.05 symptom prevalence and no symptom correlations. Major depressive episodes were not significantly associated with their symptoms in all simulations based on odds ratios, assuming no symptom correlations. Three bias variables (mde_bias1, mde_bias2, and mde_bias) were continuous variables and odds ratios were not available.

In Table 7, the minor criteria for the diagnosis of dysthymic disorder (dys_mi) often occurred with major depressive disorder and their odds ratios were not available. The other symptoms for the diagnosis of dysthymic disorder were not significantly associated with major depressive episodes in all simulations based on odds ratios, assuming no symptom correlations. One bias variable (dys_mi_bias) was a continuous variable and its odds ratios were not available.

In Table 8, there were insufficient numbers of patients with symptoms for the diagnosis of manic disorder and their odds ratios were not available. Three bias variables (man_bias1, man_bias2, and man_bias) were continuous variables and their odds ratios were not available. Major depressive episodes were not significantly associated with manic episodes’ symptoms in all simulations, when odds ratios were available.

Dysthymic disorder and psychiatric symptoms

Tables 9-11 present the correlations between dysthymic disorder and psychiatric symptoms. In Table 9, the correlations between dysthymic disorder and the symptoms of major depressive disorder were not significant in all simulations, assuming no correlations between symptoms. The correlations between dysthymic disorder and these symptoms could be strong (coefficients > 0.85, assuming 0.7 symptom prevalence and 0.9 symptom correlations, p < 0.05 for all) in the simulations.

**Table 9:** Correlations between dysthymic disorder and the input symptoms of major depressive episodes by assumed symptom prevalence and correlations Mean (standard deviation) *<number> = the numbers of significant statistics (p < 0.05) in 100 simulations !# = p < 0.001 in all of the 100 simulations !@ = p < 0.001 in some of the 100 simulations mde = Major Depressive Episodes for the diagnosis of Major Depressive Disorder; mde_ma1 = Depressed mood for more than two weeks.; mde_ma2 = Loss of interest or pleasure in daily activities for more than two weeks.; mde_mi3 = Significant unintentional weight loss or gain; mde_mi3_1 = Significant unintentional weight gain; mde_mi3_2 = Significant unintentional weight loss; mde_mi3_bias = Information of the domain not explained by the input variables; mde_mi4 = Insomnia or sleeping too much; mde_mi4_1 = Insomnia; mde_mi4_2 = Sleeping too much; mde_mi4_bias = Information of the domain not explained by the input variables; mde_mi5 = Agitation or psychomotor retardation noticed by others; mde_mi5_1 = Agitation; mde_mi5_2 = Psychomotor retardation noticed by others; mde_mi5_bias = Information of the domain not explained by the input variables; mde_mi6 = Fatigue or loss of energy; mde_mi6_1 = Fatigue; mde_mi6_2 = Loss of energy; mde_mi6_bias = Information of the domain not explained by the input variables; mde_mi7 = Feelings of worthlessness or excessive guilt; mde_mi7_1 = Feelings of worthlessness; mde_mi7_2 = Feelings of excessive guilt; mde_mi7_bias = Information of the domain not explained by the input variables; mde_mi8 = Diminished ability to think or concentrate, or indecisiveness+; mde_mi8_1 = Diminished ability to think or concentrate; mde_mi8_2 = Indecisiveness; mde_mi8_bias = Information of the domain not explained by the input variables; mde_mi9 = Recurrent thoughts of death; mde_bias1 = Information due to top censoring by choosing three domains in minor criteria; mde_bias2 = Information due to top censoring by choosing four domains in minor criteria; mde_bias = Information of diagnosis not explained by the domain

Assumed symptom prevalence	0.05	0.1	0.3	0.5	0.7	0.05	0.1	0.3	0.5	0.7	0.05	0.1	0.3	0.5	0.7	0.05	0.1	0.3	0.5	0.7	0.05	0.1	0.3	0.5	0.7
Assumed symptom correlations	0	0	0	0	0	0.1	0.1	0.1	0.1	0.1	0.4	0.4	0.4	0.4	0.4	0.7	0.7	0.7	0.7	0.7	0.9	0.9	0.9	0.9	0.9
mde	0.01 (0.04) *<8>	0.01 (0.02) *<13>	0.01 (0.01) *<27>	0 (0.01) *<7>	0 (0.01) *<3>	0.13 (0.04) *<100>!#	0.13 (0.02) *<100>!#	0.14 (0.01) *<100>!#	0.14 (0.01) *<100>!#	0.13 (0.01) *<100>!#	0.47 (0.02) *<100>!#	0.46 (0.02) *<100>!#	0.46 (0.01) *<100>!#	0.46 (0.01) *<100>!#	0.46 (0.01) *<100>!#	0.74 (0.02) *<100>!#	0.73 (0.01) *<100>!#	0.73 (0.01) *<100>!#	0.74 (0.01) *<100>!#	0.74 (0.01) *<100>!#	0.85 (0.01) *<100>!#	0.86 (0.01) *<100>!#	0.87 (0.01) *<100>!#	0.88 (0) *<100>!#	0.87 (0) *<100>!#
mde_ma1	0 (0.01) *<9>	0 (0.01) *<4>	0 (0.01) *<8>	0 (0.01) *<3>	0 (0.01) *<6>	0.12 (0.02) *<100>!#	0.12 (0.02) *<100>!#	0.12 (0.01) *<100>!#	0.12 (0.01) *<100>!#	0.11 (0.01) *<100>!#	0.44 (0.02) *<100>!#	0.44 (0.02) *<100>!#	0.44 (0.01) *<100>!#	0.43 (0.01) *<100>!#	0.42 (0.01) *<100>!#	0.72 (0.02) *<100>!#	0.72 (0.01) *<100>!#	0.72 (0.01) *<100>!#	0.72 (0.01) *<100>!#	0.72 (0.01) *<100>!#	0.85 (0.01) *<100>!#	0.85 (0.01) *<100>!#	0.87 (0.01) *<100>!#	0.87 (0) *<100>!#	0.87 (0.01) *<100>!#
mde_ma2	0 (0.01) *<4>	0 (0.01) *<3>	0 (0.01) *<7>	0 (0.01) *<5>	0 (0.01) *<3>	0.12 (0.02) *<100>!#	0.12 (0.01) *<100>!#	0.12 (0.01) *<100>!#	0.12 (0.01) *<100>!#	0.11 (0.01) *<100>!#	0.44 (0.02) *<100>!#	0.44 (0.02) *<100>!#	0.44 (0.01) *<100>!#	0.43 (0.01) *<100>!#	0.42 (0.01) *<100>!#	0.72 (0.02) *<100>!#	0.72 (0.01) *<100>!#	0.72 (0.01) *<100>!#	0.72 (0.01) *<100>!#	0.72 (0.01) *<100>!#	0.85 (0.01) *<100>!#	0.85 (0.01) *<100>!#	0.87 (0.01) *<100>!#	0.87 (0) *<100>!#	0.87 (0.01) *<100>!#
mde_mi3	0 (0.01) *<9>	0 (0.01) *<5>	0 (0.01) *<3>	0 (0.01) *<6>	0 (0.01) *<8>	0.15 (0.02) *<100>!#	0.15 (0.01) *<100>!#	0.15 (0.01) *<100>!#	0.13 (0.01) *<100>!#	0.1 (0.01) *<100>!#	0.47 (0.02) *<100>!#	0.47 (0.01) *<100>!#	0.47 (0.01) *<100>!#	0.45 (0.01) *<100>!#	0.43 (0.01) *<100>!#	0.73 (0.01) *<100>!#	0.73 (0.01) *<100>!#	0.73 (0.01) *<100>!#	0.72 (0.01) *<100>!#	0.72 (0.01) *<100>!#	0.85 (0.01) *<100>!#	0.86 (0.01) *<100>!#	0.87 (0.01) *<100>!#	0.87 (0) *<100>!#	0.87 (0.01) *<100>!#
mde_mi3_1	0 (0.01) *<5>	0 (0.01) *<8>	0 (0.01) *<1>	0 (0.01) *<5>	0 (0.01) *<4>	0.12 (0.02) *<100>!#	0.12 (0.01) *<100>!#	0.12 (0.01) *<100>!#	0.12 (0.01) *<100>!#	0.11 (0.01) *<100>!#	0.44 (0.02) *<100>!#	0.44 (0.02) *<100>!#	0.44 (0.01) *<100>!#	0.43 (0.01) *<100>!#	0.42 (0.01) *<100>!#	0.72 (0.02) *<100>!#	0.72 (0.01) *<100>!#	0.72 (0.01) *<100>!#	0.72 (0.01) *<100>!#	0.72 (0.01) *<100>!#	0.85 (0.01) *<100>!#	0.85 (0.01) *<100>!#	0.87 (0) *<100>!#	0.87 (0) *<100>!#	0.87 (0.01) *<100>!#
mde_mi3_2	0 (0.01) *<5>	0 (0.01) *<8>	0 (0.01) *<4>	0 (0.01) *<3>	0 (0.01) *<2>	0.12 (0.02) *<100>!#	0.12 (0.01) *<100>!#	0.12 (0.01) *<100>!#	0.12 (0.01) *<100>!#	0.11 (0.01) *<100>!#	0.44 (0.02) *<100>!#	0.44 (0.02) *<100>!#	0.44 (0.01) *<100>!#	0.43 (0.01) *<100>!#	0.42 (0.01) *<100>!#	0.72 (0.02) *<100>!#	0.72 (0.01) *<100>!#	0.72 (0.01) *<100>!#	0.72 (0.01) *<100>!#	0.72 (0.01) *<100>!#	0.85 (0.01) *<100>!#	0.86 (0.01) *<100>!#	0.87 (0.01) *<100>!#	0.87 (0) *<100>!#	0.87 (0.01) *<100>!#
mde_mi3_bias	0 (0.01) *<10>	0 (0.01) *<5>	0 (0.01) *<3>	0 (0.01) *<5>	0 (0.01) *<2>	-0.11 (0.03) *<100>!#	-0.11 (0.02) *<100>!#	-0.12 (0.01) *<100>!#	-0.13 (0.01) *<100>!#	-0.13 (0.01) *<100>!#	-0.44 (0.02) *<100>!#	-0.45 (0.02) *<100>!#	-0.45 (0.01) *<100>!#	-0.45 (0.01) *<100>!#	-0.45 (0.01) *<100>!#	-0.73 (0.02) *<100>!#	-0.73 (0.01) *<100>!#	-0.73 (0.01) *<100>!#	-0.73 (0.01) *<100>!#	-0.73 (0.01) *<100>!#	-0.85 (0.01) *<100>!#	-0.86 (0.01) *<100>!#	-0.87 (0.01) *<100>!#	-0.87 (0) *<100>!#	-0.87 (0.01) *<100>!#
mde_mi4	0.07 (0.02) *<100>	0.1 (0.01) *<100>!#	0.08 (0.01) *<100>!#	0.02 (0.01) *<48>	0 (0.01) *<7>	0.21 (0.02) *<100>!#	0.22 (0.01) *<100>!#	0.2 (0.01) *<100>!#	0.16 (0.01) *<100>!#	0.12 (0.01) *<100>!#	0.5 (0.02) *<100>!#	0.5 (0.01) *<100>!#	0.49 (0.01) *<100>!#	0.47 (0.01) *<100>!#	0.45 (0.01) *<100>!#	0.74 (0.01) *<100>!#	0.74 (0.01) *<100>!#	0.74 (0.01) *<100>!#	0.73 (0.01) *<100>!#	0.73 (0.01) *<100>!#	0.86 (0.01) *<100>!#	0.86 (0.01) *<100>!#	0.87 (0.01) *<100>!#	0.87 (0) *<100>!#	0.87 (0.01) *<100>!#
mde_mi4_1	0.05 (0.02) *<89>	0.07 (0.01) *<100>!@	0.05 (0.01) *<100>	0.01 (0.01) *<16>	0 (0.01) *<8>	0.16 (0.02) *<100>!#	0.17 (0.02) *<100>!#	0.16 (0.01) *<100>!#	0.13 (0.01) *<100>!#	0.11 (0.01) *<100>!#	0.46 (0.02) *<100>!#	0.46 (0.02) *<100>!#	0.45 (0.01) *<100>!#	0.44 (0.01) *<100>!#	0.43 (0.01) *<100>!#	0.73 (0.01) *<100>!#	0.73 (0.01) *<100>!#	0.73 (0.01) *<100>!#	0.73 (0.01) *<100>!#	0.72 (0.01) *<100>!#	0.85 (0.01) *<100>!#	0.86 (0.01) *<100>!#	0.87 (0.01) *<100>!#	0.87 (0) *<100>!#	0.87 (0.01) *<100>!#
mde_mi4_2	0.05 (0.02) *<93>	0.07 (0.02) *<100>!@	0.05 (0.01) *<100>	0.01 (0.01) *<24>	0 (0.01) *<8>	0.16 (0.02) *<100>!#	0.17 (0.02) *<100>!#	0.16 (0.01) *<100>!#	0.13 (0.01) *<100>!#	0.11 (0.01) *<100>!#	0.46 (0.02) *<100>!#	0.46 (0.02) *<100>!#	0.45 (0.01) *<100>!#	0.44 (0.01) *<100>!#	0.43 (0.01) *<100>!#	0.73 (0.02) *<100>!#	0.72 (0.01) *<100>!#	0.73 (0.01) *<100>!#	0.73 (0.01) *<100>!#	0.72 (0.01) *<100>!#	0.85 (0.01) *<100>!#	0.86 (0.01) *<100>!#	0.87 (0.01) *<100>!#	0.87 (0) *<100>!#	0.87 (0.01) *<100>!#
mde_mi4_bias	-0.01 (0.02) *<33>	-0.02 (0.01) *<54>	-0.02 (0.01) *<62>	-0.01 (0.01) *<9>	0 (0.01) *<3>	-0.13 (0.03) *<100>!#	-0.13 (0.02) *<100>!#	-0.13 (0.01) *<100>!#	-0.14 (0.01) *<100>!#	-0.13 (0.01) *<100>!#	-0.46 (0.03) *<100>!#	-0.45 (0.02) *<100>!#	-0.45 (0.01) *<100>!#	-0.46 (0.01) *<100>!#	-0.45 (0.01) *<100>!#	-0.73 (0.02) *<100>!#	-0.73 (0.01) *<100>!#	-0.73 (0.01) *<100>!#	-0.73 (0.01) *<100>!#	-0.73 (0.01) *<100>!#	-0.85 (0.01) *<100>!#	-0.86 (0.01) *<100>!#	-0.87 (0.01) *<100>!#	-0.87 (0) *<100>!#	-0.87 (0) *<100>!#
mde_mi5	0 (0.01) *<3>	0 (0.01) *<4>	0 (0.01) *<3>	0 (0.01) *<1>	0 (0.01) *<5>	0.15 (0.02) *<100>!#	0.15 (0.01) *<100>!#	0.15 (0.01) *<100>!#	0.13 (0.01) *<100>!#	0.11 (0.01) *<100>!#	0.47 (0.02) *<100>!#	0.47 (0.01) *<100>!#	0.46 (0.01) *<100>!#	0.45 (0.01) *<100>!#	0.43 (0.01) *<100>!#	0.73 (0.01) *<100>!#	0.73 (0.01) *<100>!#	0.73 (0.01) *<100>!#	0.72 (0.01) *<100>!#	0.72 (0.01) *<100>!#	0.85 (0.01) *<100>!#	0.86 (0.01) *<100>!#	0.87 (0.01) *<100>!#	0.87 (0) *<100>!#	0.87 (0.01) *<100>!#
mde_mi5_1	0 (0.01) *<3>	0 (0.01) *<5>	0 (0.01) *<2>	0 (0.01) *<1>	0 (0.01) *<7>	0.12 (0.02) *<100>!#	0.12 (0.01) *<100>!#	0.12 (0.01) *<100>!#	0.12 (0.01) *<100>!#	0.1 (0.01) *<100>!#	0.44 (0.02) *<100>!#	0.44 (0.02) *<100>!#	0.43 (0.01) *<100>!#	0.43 (0.01) *<100>!#	0.42 (0.01) *<100>!#	0.72 (0.02) *<100>!#	0.72 (0.01) *<100>!#	0.72 (0.01) *<100>!#	0.72 (0.01) *<100>!#	0.72 (0.01) *<100>!#	0.85 (0.01) *<100>!#	0.85 (0.01) *<100>!#	0.87 (0.01) *<100>!#	0.87 (0) *<100>!#	0.87 (0.01) *<100>!#
mde_mi5_2	0 (0.01) *<1>	0 (0.01) *<1>	0 (0.01) *<2>	0 (0.01) *<6>	0 (0.01) *<2>	0.12 (0.02) *<100>!#	0.12 (0.01) *<100>!#	0.12 (0.01) *<100>!#	0.12 (0.01) *<100>!#	0.11 (0.01) *<100>!#	0.44 (0.02) *<100>!#	0.44 (0.01) *<100>!#	0.43 (0.01) *<100>!#	0.43 (0.01) *<100>!#	0.42 (0.01) *<100>!#	0.72 (0.02) *<100>!#	0.72 (0.01) *<100>!#	0.72 (0.01) *<100>!#	0.72 (0.01) *<100>!#	0.72 (0.01) *<100>!#	0.85 (0.01) *<100>!#	0.85 (0.01) *<100>!#	0.87 (0.01) *<100>!#	0.87 (0) *<100>!#	0.87 (0.01) *<100>!#
mde_mi5_bias	0 (0.01) *<13>	0 (0.01) *<4>	0 (0.01) *<2>	0 (0.01) *<5>	0 (0.01) *<7>	-0.11 (0.03) *<100>!#	-0.11 (0.02) *<100>!#	-0.12 (0.01) *<100>!#	-0.13 (0.01) *<100>!#	-0.13 (0.01) *<100>!#	-0.45 (0.03) *<100>!#	-0.44 (0.02) *<100>!#	-0.45 (0.01) *<100>!#	-0.45 (0.01) *<100>!#	-0.45 (0.01) *<100>!#	-0.73 (0.02) *<100>!#	-0.73 (0.01) *<100>!#	-0.73 (0.01) *<100>!#	-0.73 (0.01) *<100>!#	-0.73 (0.01) *<100>!#	-0.85 (0.01) *<100>!#	-0.86 (0.01) *<100>!#	-0.87 (0.01) *<100>!#	-0.87 (0) *<100>!#	-0.87 (0) *<100>!#
mde_mi6	0.07 (0.02) *<100>!@	0.1 (0.01) *<100>!#	0.08 (0.01) *<100>!#	0.02 (0.01) *<44>	0 (0.01) *<8>	0.21 (0.02) *<100>!#	0.22 (0.01) *<100>!#	0.2 (0.01) *<100>!#	0.16 (0.01) *<100>!#	0.12 (0.01) *<100>!#	0.5 (0.02) *<100>!#	0.5 (0.01) *<100>!#	0.49 (0.01) *<100>!#	0.47 (0.01) *<100>!#	0.45 (0.01) *<100>!#	0.74 (0.01) *<100>!#	0.74 (0.01) *<100>!#	0.74 (0.01) *<100>!#	0.73 (0.01) *<100>!#	0.73 (0.01) *<100>!#	0.86 (0.01) *<100>!#	0.86 (0.01) *<100>!#	0.87 (0.01) *<100>!#	0.87 (0) *<100>!#	0.87 (0.01) *<100>!#
mde_mi6_1	0.05 (0.02) *<96>	0.07 (0.02) *<100>!#	0.05 (0.01) *<100>	0.01 (0.01) *<24>	0 (0.01) *<5>	0.17 (0.02) *<100>!#	0.17 (0.02) *<100>!#	0.16 (0.01) *<100>!#	0.13 (0.01) *<100>!#	0.11 (0.01) *<100>!#	0.46 (0.02) *<100>!#	0.46 (0.02) *<100>!#	0.45 (0.01) *<100>!#	0.44 (0.01) *<100>!#	0.43 (0.01) *<100>!#	0.73 (0.02) *<100>!#	0.72 (0.01) *<100>!#	0.73 (0.01) *<100>!#	0.72 (0.01) *<100>!#	0.72 (0.01) *<100>!#	0.85 (0.01) *<100>!#	0.86 (0.01) *<100>!#	0.87 (0) *<100>!#	0.87 (0) *<100>!#	0.87 (0.01) *<100>!#
mde_mi6_2	0.04 (0.02) *<91>	0.07 (0.01) *<100>!#	0.05 (0.01) *<100>	0.01 (0.01) *<19>	0 (0.01) *<8>	0.17 (0.02) *<100>!#	0.17 (0.01) *<100>!#	0.16 (0.01) *<100>!#	0.13 (0.01) *<100>!#	0.11 (0.01) *<100>!#	0.46 (0.02) *<100>!#	0.46 (0.02) *<100>!#	0.45 (0.01) *<100>!#	0.44 (0.01) *<100>!#	0.43 (0.01) *<100>!#	0.73 (0.02) *<100>!#	0.72 (0.01) *<100>!#	0.73 (0.01) *<100>!#	0.72 (0.01) *<100>!#	0.72 (0.01) *<100>!#	0.85 (0.01) *<100>!#	0.86 (0.01) *<100>!#	0.87 (0.01) *<100>!#	0.87 (0.01) *<100>!#	0.87 (0.01) *<100>!#
mde_mi6_bias	-0.01 (0.02) *<29>	-0.02 (0.01) *<54>	-0.03 (0.01) *<70>	-0.01 (0.01) *<13>	0 (0.01) *<3>	-0.13 (0.03) *<100>!#	-0.13 (0.02) *<100>!#	-0.14 (0.01) *<100>!#	-0.14 (0.01) *<100>!#	-0.13 (0.01) *<100>!#	-0.45 (0.02) *<100>!#	-0.45 (0.02) *<100>!#	-0.45 (0.01) *<100>!#	-0.46 (0.01) *<100>!#	-0.45 (0.01) *<100>!#	-0.73 (0.02) *<100>!#	-0.73 (0.01) *<100>!#	-0.73 (0.01) *<100>!#	-0.73 (0.01) *<100>!#	-0.73 (0.01) *<100>!#	-0.85 (0.01) *<100>!#	-0.86 (0.01) *<100>!#	-0.87 (0) *<100>!#	-0.87 (0) *<100>!#	-0.87 (0) *<100>!#
mde_mi7	0 (0.01) *<6>	0 (0.01) *<2>	0 (0.01) *<3>	0 (0.01) *<5>	0 (0.01) *<4>	0.15 (0.02) *<100>!#	0.15 (0.01) *<100>!#	0.15 (0.01) *<100>!#	0.13 (0.01) *<100>!#	0.1 (0.01) *<100>!#	0.47 (0.02) *<100>!#	0.47 (0.01) *<100>!#	0.47 (0.01) *<100>!#	0.45 (0.01) *<100>!#	0.43 (0.01) *<100>!#	0.73 (0.02) *<100>!#	0.73 (0.01) *<100>!#	0.73 (0.01) *<100>!#	0.72 (0.01) *<100>!#	0.72 (0.01) *<100>!#	0.85 (0.01) *<100>!#	0.86 (0.01) *<100>!#	0.87 (0.01) *<100>!#	0.87 (0) *<100>!#	0.86 (0.01) *<100>!#
mde_mi7_1	0 (0.01) *<6>	0 (0.01) *<4>	0 (0.01) *<7>	0 (0.01) *<8>	0 (0.01) *<7>	0.12 (0.02) *<100>!#	0.12 (0.01) *<100>!#	0.12 (0.01) *<100>!#	0.12 (0.01) *<100>!#	0.1 (0.01) *<100>!#	0.44 (0.02) *<100>!#	0.44 (0.01) *<100>!#	0.44 (0.01) *<100>!#	0.43 (0.01) *<100>!#	0.42 (0.01) *<100>!#	0.72 (0.02) *<100>!#	0.72 (0.01) *<100>!#	0.72 (0.01) *<100>!#	0.72 (0.01) *<100>!#	0.72 (0.01) *<100>!#	0.85 (0.01) *<100>!#	0.85 (0.01) *<100>!#	0.87 (0.01) *<100>!#	0.87 (0) *<100>!#	0.87 (0.01) *<100>!#
mde_mi7_2	0 (0.01) *<4>	0 (0.01) *<4>	0 (0.01) *<3>	0 (0.01) *<4>	0 (0.01) *<4>	0.12 (0.02) *<100>!#	0.12 (0.01) *<100>!#	0.12 (0.01) *<100>!#	0.12 (0.01) *<100>!#	0.1 (0.01) *<100>!#	0.44 (0.02) *<100>!#	0.44 (0.02) *<100>!#	0.44 (0.01) *<100>!#	0.43 (0.01) *<100>!#	0.42 (0.01) *<100>!#	0.72 (0.02) *<100>!#	0.72 (0.01) *<100>!#	0.72 (0.01) *<100>!#	0.72 (0.01) *<100>!#	0.72 (0.01) *<100>!#	0.85 (0.01) *<100>!#	0.85 (0.01) *<100>!#	0.87 (0.01) *<100>!#	0.87 (0.01) *<100>!#	0.87 (0.01) *<100>!#
mde_mi7_bias	0 (0.01) *<11>	0 (0.01) *<3>	0 (0.01) *<5>	0 (0.01) *<4>	0 (0.01) *<4>	-0.11 (0.03) *<100>!#	-0.11 (0.02) *<100>!#	-0.12 (0.01) *<100>!#	-0.13 (0.01) *<100>!#	-0.13 (0.01) *<100>!#	-0.45 (0.03) *<100>!#	-0.44 (0.02) *<100>!#	-0.45 (0.01) *<100>!#	-0.45 (0.01) *<100>!#	-0.45 (0.01) *<100>!#	-0.73 (0.02) *<100>!#	-0.73 (0.01) *<100>!#	-0.73 (0.01) *<100>!#	-0.73 (0.01) *<100>!#	-0.73 (0.01) *<100>!#	-0.85 (0.01) *<100>!#	-0.86 (0.01) *<100>!#	-0.87 (0.01) *<100>!#	-0.87 (0) *<100>!#	-0.87 (0.01) *<100>!#
mde_mi8	0.07 (0.02) *<99>	0.1 (0.01) *<100>!#	0.08 (0.01) *<100>!#	0.02 (0.01) *<47>	0 (0.01) *<4>	0.21 (0.02) *<100>!#	0.22 (0.01) *<100>!#	0.2 (0.01) *<100>!#	0.16 (0.01) *<100>!#	0.11 (0.01) *<100>!#	0.5 (0.02) *<100>!#	0.5 (0.01) *<100>!#	0.49 (0.01) *<100>!#	0.47 (0.01) *<100>!#	0.44 (0.01) *<100>!#	0.74 (0.01) *<100>!#	0.74 (0.01) *<100>!#	0.74 (0.01) *<100>!#	0.73 (0.01) *<100>!#	0.73 (0.01) *<100>!#	0.86 (0.01) *<100>!#	0.86 (0.01) *<100>!#	0.87 (0.01) *<100>!#	0.87 (0) *<100>!#	0.87 (0) *<100>!#
mde_mi8_1	0.04 (0.02) *<83>	0.07 (0.01) *<100>!#	0.05 (0.01) *<100>	0.01 (0.01) *<22>	0 (0.01) *<4>	0.16 (0.02) *<100>!#	0.17 (0.02) *<100>!#	0.16 (0.01) *<100>!#	0.13 (0.01) *<100>!#	0.11 (0.01) *<100>!#	0.46 (0.02) *<100>!#	0.46 (0.01) *<100>!#	0.45 (0.01) *<100>!#	0.44 (0.01) *<100>!#	0.43 (0.01) *<100>!#	0.73 (0.01) *<100>!#	0.72 (0.01) *<100>!#	0.73 (0.01) *<100>!#	0.73 (0.01) *<100>!#	0.72 (0.01) *<100>!#	0.85 (0.01) *<100>!#	0.86 (0.01) *<100>!#	0.87 (0.01) *<100>!#	0.87 (0) *<100>!#	0.87 (0.01) *<100>!#
mde_mi8_2	0.05 (0.02) *<93>	0.07 (0.01) *<100>!#	0.05 (0.01) *<100>	0.01 (0.01) *<19>	0 (0.01) *<5>	0.17 (0.02) *<100>!#	0.17 (0.01) *<100>!#	0.16 (0.01) *<100>!#	0.13 (0.01) *<100>!#	0.11 (0.01) *<100>!#	0.46 (0.02) *<100>!#	0.46 (0.01) *<100>!#	0.45 (0.01) *<100>!#	0.44 (0.01) *<100>!#	0.43 (0.01) *<100>!#	0.73 (0.01) *<100>!#	0.72 (0.01) *<100>!#	0.73 (0.01) *<100>!#	0.73 (0.01) *<100>!#	0.72 (0.01) *<100>!#	0.85 (0.01) *<100>!#	0.86 (0.01) *<100>!#	0.87 (0.01) *<100>!#	0.87 (0.01) *<100>!#	0.87 (0.01) *<100>!#
mde_mi8_bias	-0.01 (0.02) *<29>	-0.02 (0.01) *<51>	-0.03 (0.01) *<78>	-0.01 (0.01) *<1>	0 (0.01) *<3>	-0.13 (0.03) *<100>!#	-0.13 (0.02) *<100>!#	-0.14 (0.01) *<100>!#	-0.14 (0.01) *<100>!#	-0.13 (0.01) *<100>!#	-0.45 (0.03) *<100>!#	-0.45 (0.02) *<100>!#	-0.45 (0.01) *<100>!#	-0.46 (0.01) *<100>!#	-0.45 (0.01) *<100>!#	-0.73 (0.02) *<100>!#	-0.73 (0.01) *<100>!#	-0.73 (0.01) *<100>!#	-0.73 (0.01) *<100>!#	-0.73 (0.01) *<100>!#	-0.85 (0.01) *<100>!#	-0.86 (0.01) *<100>!#	-0.87 (0) *<100>!#	-0.87 (0) *<100>!#	-0.87 (0.01) *<100>!#
mde_mi9	0 (0.01) *<2>	0 (0.01) *<4>	0 (0.01) *<8>	0 (0.01) *<7>	0 (0.01) *<5>	0.12 (0.02) *<100>!#	0.12 (0.01) *<100>!#	0.12 (0.01) *<100>!#	0.12 (0.01) *<100>!#	0.11 (0.01) *<100>!#	0.44 (0.02) *<100>!#	0.44 (0.02) *<100>!#	0.44 (0.01) *<100>!#	0.43 (0.01) *<100>!#	0.42 (0.01) *<100>!#	0.72 (0.02) *<100>!#	0.72 (0.01) *<100>!#	0.72 (0.01) *<100>!#	0.72 (0.01) *<100>!#	0.72 (0.01) *<100>!#	0.85 (0.01) *<100>!#	0.85 (0.01) *<100>!#	0.87 (0.01) *<100>!#	0.87 (0) *<100>!#	0.87 (0.01) *<100>!#
mde_bias1	-0.07 (0.01) *<100>!#	-0.11 (0.01) *<100>!#	-0.08 (0.01) *<100>!#	-0.02 (0.01) *<53>	0 (0.01) *<5>	-0.31 (0.02) *<100>!#	-0.33 (0.01) *<100>!#	-0.32 (0.01) *<100>!#	-0.28 (0.01) *<100>!#	-0.22 (0.01) *<100>!#	-0.63 (0.01) *<100>!#	-0.64 (0.01) *<100>!#	-0.64 (0.01) *<100>!#	-0.63 (0.01) *<100>!#	-0.61 (0.01) *<100>!#	-0.83 (0.01) *<100>!#	-0.83 (0.01) *<100>!#	-0.83 (0) *<100>!#	-0.83 (0) *<100>!#	-0.83 (0) *<100>!#	-0.91 (0.01) *<100>!#	-0.91 (0.01) *<100>!#	-0.92 (0) *<100>!#	-0.92 (0) *<100>!#	-0.92 (0) *<100>!#
mde_bias2	-0.08 (0.01) *<100>!#	-0.12 (0.01) *<100>!#	-0.09 (0.01) *<100>!#	-0.02 (0.01) *<46>	0 (0.01) *<5>	-0.31 (0.02) *<100>!#	-0.33 (0.01) *<100>!#	-0.32 (0.01) *<100>!#	-0.27 (0.01) *<100>!#	-0.22 (0.01) *<100>!#	-0.63 (0.01) *<100>!#	-0.63 (0.01) *<100>!#	-0.63 (0.01) *<100>!#	-0.63 (0.01) *<100>!#	-0.6 (0.01) *<100>!#	-0.83 (0.01) *<100>!#	-0.82 (0.01) *<100>!#	-0.83 (0) *<100>!#	-0.83 (0) *<100>!#	-0.82 (0) *<100>!#	-0.91 (0.01) *<100>!#	-0.91 (0.01) *<100>!#	-0.92 (0) *<100>!#	-0.92 (0) *<100>!#	-0.92 (0) *<100>!#
mde_bias	0.07 (0.01) *<100>!#	0.09 (0.01) *<100>!#	0.06 (0.01) *<100>!#	0.02 (0.01) *<32>	0 (0.01) *<5>	0.23 (0.01) *<100>!#	0.24 (0.01) *<100>!#	0.23 (0.01) *<100>!#	0.22 (0.01) *<100>!#	0.19 (0.01) *<100>!#	0.56 (0.01) *<100>!#	0.57 (0.01) *<100>!#	0.58 (0.01) *<100>!#	0.58 (0.01) *<100>!#	0.56 (0.01) *<100>!#	0.81 (0.01) *<100>!#	0.8 (0.01) *<100>!#	0.81 (0) *<100>!#	0.81 (0) *<100>!#	0.81 (0.01) *<100>!#	0.9 (0.01) *<100>!#	0.9 (0.01) *<100>!#	0.91 (0) *<100>!#	0.91 (0) *<100>!#	0.91 (0) *<100>!#

**Table 10:** Correlations between dysthymic disorder and its input symptoms by assumed symptom prevalence and correlations Mean (standard deviation) *<number> = the numbers of significant statistics (p < 0.05) in 100 simulations !# = p < 0.001 in all of the 100 simulations !@ = p < 0.001 in some of the 100 simulations Note: See Table 1 for variable definitions and Table 2 for the prevalence of major depressive episodes, dysthymic disorder, and manic episodes. dys = Dysthymic Disorder; dys_ma = Depressed mood most of the day for more days than not, for at least 2 years; dys_mi = Minor criteria (at least 2 items); dys_mi1 = Poor appetite or overeating; dys_mi1_1 = Poor appetite; dys_mi1_2 = Overeating; dys_mi1_bias = Information of the domain not explained by the input variables; dys_mi4 = Low self-esteem; dys_mi6 = Feelings of hopelessness; dys_mi_bias = Information of minor criteria not explained by input variables; dys_bias = Information of diagnosis not explained by major or minor criteria

Assumed symptom prevalence	0.05	0.1	0.3	0.5	0.7	0.05	0.1	0.3	0.5	0.7	0.05	0.1	0.3	0.5	0.7	0.05	0.1	0.3	0.5	0.7	0.05	0.1	0.3	0.5	0.7
Assumed symptom correlations	0	0	0	0	0	0.1	0.1	0.1	0.1	0.1	0.4	0.4	0.4	0.4	0.4	0.7	0.7	0.7	0.7	0.7	0.9	0.9	0.9	0.9	0.9
dys	1 (0) *<100>!#	1 (0) *<100>!#	1 (0) *<100>!#	1 (0) *<100>!#	1 (0) *<100>!#	1 (0) *<100>!#	1 (0) *<100>!#	1 (0) *<100>!#	1 (0) *<100>!#	1 (0) *<100>!#	1 (0) *<100>!#	1 (0) *<100>!#	1 (0) *<100>!#	1 (0) *<100>!#	1 (0) *<100>!#	1 (0) *<100>!#	1 (0) *<100>!#	1 (0) *<100>!#	1 (0) *<100>!#	1 (0) *<100>!#	1 (0) *<100>!#	1 (0) *<100>!#	1 (0) *<100>!#	1 (0) *<100>!#	1 (0) *<100>!#
dys_ma	0.27 (0.02) *<100>!#	0.48 (0.01) *<100>!#	0.88 (0.01) *<100>!#	0.99 (0) *<100>!#	1 (0) *<100>!#	0.59 (0.02) *<100>!#	0.68 (0.01) *<100>!#	0.88 (0.01) *<100>!#	0.96 (0) *<100>!#	0.99 (0) *<100>!#	0.85 (0.01) *<100>!#	0.87 (0.01) *<100>!#	0.92 (0) *<100>!#	0.95 (0) *<100>!#	0.97 (0) *<100>!#	0.95 (0.01) *<100>!#	0.95 (0) *<100>!#	0.96 (0) *<100>!#	0.97 (0) *<100>!#	0.98 (0) *<100>!#	0.98 (0.01) *<100>!#	0.98 (0) *<100>!#	0.98 (0) *<100>!#	0.99 (0) *<100>!#	0.99 (0) *<100>!#
dys_mi	0.21 (0.02) *<100>!#	0.28 (0.01) *<100>!#	0.26 (0) *<100>!#	0.12 (0) *<100>!#	0.03 (0.01) *<82>	0.39 (0.02) *<100>!#	0.4 (0.01) *<100>!#	0.35 (0) *<100>!#	0.25 (0.01) *<100>!#	0.13 (0.01) *<100>!#	0.59 (0.01) *<100>!#	0.59 (0.01) *<100>!#	0.56 (0.01) *<100>!#	0.52 (0.01) *<100>!#	0.46 (0.01) *<100>!#	0.77 (0.01) *<100>!#	0.76 (0.01) *<100>!#	0.76 (0.01) *<100>!#	0.74 (0) *<100>!#	0.73 (0.01) *<100>!#	0.86 (0.01) *<100>!#	0.87 (0.01) *<100>!#	0.87 (0) *<100>!#	0.87 (0) *<100>!#	0.87 (0.01) *<100>!#
dys_mi1	0.07 (0.02) *<99>	0.1 (0.01) *<100>!#	0.08 (0.01) *<100>!#	0.02 (0.01) *<42>	0 (0.01) *<3>	0.21 (0.02) *<100>!#	0.22 (0.01) *<100>!#	0.2 (0.01) *<100>!#	0.16 (0.01) *<100>!#	0.12 (0.01) *<100>!#	0.5 (0.02) *<100>!#	0.5 (0.01) *<100>!#	0.49 (0.01) *<100>!#	0.47 (0.01) *<100>!#	0.44 (0.01) *<100>!#	0.74 (0.01) *<100>!#	0.74 (0.01) *<100>!#	0.74 (0.01) *<100>!#	0.73 (0.01) *<100>!#	0.73 (0.01) *<100>!#	0.85 (0.01) *<100>!#	0.86 (0.01) *<100>!#	0.87 (0) *<100>!#	0.87 (0) *<100>!#	0.87 (0.01) *<100>!#
dys_mi1_1	0.05 (0.02) *<92>	0.07 (0.01) *<100>!#	0.05 (0.01) *<100>	0.01 (0.01) *<12>	0 (0.01) *<7>	0.17 (0.02) *<100>!#	0.17 (0.02) *<100>!#	0.16 (0.01) *<100>!#	0.13 (0.01) *<100>!#	0.11 (0.01) *<100>!#	0.46 (0.02) *<100>!#	0.46 (0.02) *<100>!#	0.45 (0.01) *<100>!#	0.44 (0.01) *<100>!#	0.43 (0.01) *<100>!#	0.73 (0.02) *<100>!#	0.72 (0.01) *<100>!#	0.73 (0.01) *<100>!#	0.73 (0.01) *<100>!#	0.72 (0.01) *<100>!#	0.85 (0.01) *<100>!#	0.86 (0.01) *<100>!#	0.87 (0.01) *<100>!#	0.87 (0) *<100>!#	0.87 (0.01) *<100>!#
dys_mi1_2	0.05 (0.02) *<91>	0.07 (0.01) *<100>!@	0.05 (0.01) *<100>!@	0.01 (0.01) *<15>	0 (0.01) *<5>	0.17 (0.02) *<100>!#	0.17 (0.02) *<100>!#	0.16 (0.01) *<100>!#	0.13 (0.01) *<100>!#	0.11 (0.01) *<100>!#	0.46 (0.02) *<100>!#	0.46 (0.01) *<100>!#	0.45 (0.01) *<100>!#	0.44 (0.01) *<100>!#	0.43 (0.01) *<100>!#	0.73 (0.01) *<100>!#	0.72 (0.01) *<100>!#	0.73 (0.01) *<100>!#	0.73 (0.01) *<100>!#	0.72 (0.01) *<100>!#	0.85 (0.01) *<100>!#	0.86 (0.01) *<100>!#	0.87 (0.01) *<100>!#	0.87 (0.01) *<100>!#	0.87 (0.01) *<100>!#
dys_mi1_bias	-0.01 (0.02) *<30>	-0.02 (0.02) *<48>	-0.02 (0.01) *<64>	-0.01 (0.01) *<7>	0 (0.01) *<7>	-0.13 (0.03) *<100>!#	-0.13 (0.02) *<100>!#	-0.13 (0.01) *<100>!#	-0.13 (0.01) *<100>!#	-0.13 (0.01) *<100>!#	-0.45 (0.03) *<100>!#	-0.45 (0.02) *<100>!#	-0.45 (0.01) *<100>!#	-0.46 (0.01) *<100>!#	-0.45 (0.01) *<100>!#	-0.73 (0.02) *<100>!#	-0.73 (0.01) *<100>!#	-0.73 (0.01) *<100>!#	-0.73 (0.01) *<100>!#	-0.73 (0.01) *<100>!#	-0.85 (0.01) *<100>!#	-0.86 (0.01) *<100>!#	-0.87 (0.01) *<100>!#	-0.87 (0) *<100>!#	-0.87 (0) *<100>!#
dys_mi4	0.05 (0.02) *<93>	0.08 (0.01) *<100>!#	0.06 (0.01) *<100>!@	0.01 (0.01) *<23>	0 (0.01) *<7>	0.17 (0.02) *<100>!#	0.18 (0.02) *<100>!#	0.16 (0.01) *<100>!#	0.13 (0.01) *<100>!#	0.11 (0.01) *<100>!#	0.46 (0.02) *<100>!#	0.46 (0.02) *<100>!#	0.45 (0.01) *<100>!#	0.45 (0.01) *<100>!#	0.43 (0.01) *<100>!#	0.73 (0.02) *<100>!#	0.73 (0.01) *<100>!#	0.73 (0.01) *<100>!#	0.73 (0.01) *<100>!#	0.72 (0.01) *<100>!#	0.85 (0.01) *<100>!#	0.86 (0.01) *<100>!#	0.87 (0.01) *<100>!#	0.87 (0) *<100>!#	0.87 (0.01) *<100>!#
dys_mi6	0.05 (0.02) *<98>	0.08 (0.01) *<100>!#	0.06 (0.01) *<100>!@	0.01 (0.01) *<24>	0 (0.01) *<2>	0.17 (0.02) *<100>!#	0.18 (0.02) *<100>!#	0.16 (0.01) *<100>!#	0.14 (0.01) *<100>!#	0.11 (0.01) *<100>!#	0.46 (0.02) *<100>!#	0.46 (0.02) *<100>!#	0.45 (0.01) *<100>!#	0.45 (0.01) *<100>!#	0.43 (0.01) *<100>!#	0.73 (0.02) *<100>!#	0.73 (0.01) *<100>!#	0.73 (0.01) *<100>!#	0.73 (0.01) *<100>!#	0.72 (0.01) *<100>!#	0.85 (0.01) *<100>!#	0.86 (0.01) *<100>!#	0.87 (0.01) *<100>!#	0.87 (0) *<100>!#	0.87 (0.01) *<100>!#
dys_mi_bias	-0.09 (0.01) *<100>!#	-0.15 (0.01) *<100>!#	-0.12 (0.01) *<100>!#	-0.03 (0.01) *<78>	0 (0.01) *<10>	-0.32 (0.02) *<100>!#	-0.34 (0.01) *<100>!#	-0.33 (0.01) *<100>!#	-0.27 (0.01) *<100>!#	-0.22 (0.01) *<100>!#	-0.64 (0.01) *<100>!#	-0.65 (0.01) *<100>!#	-0.64 (0.01) *<100>!#	-0.63 (0.01) *<100>!#	-0.6 (0.01) *<100>!#	-0.84 (0.01) *<100>!#	-0.84 (0.01) *<100>!#	-0.84 (0) *<100>!#	-0.83 (0) *<100>!#	-0.83 (0) *<100>!#	-0.91 (0.01) *<100>!#	-0.92 (0.01) *<100>!#	-0.92 (0) *<100>!#	-0.92 (0) *<100>!#	-0.92 (0) *<100>!#
dys_bias	-0.17 (0.01) *<100>!#	-0.23 (0.01) *<100>!#	-0.21 (0) *<100>!#	-0.08 (0.01) *<100>!#	-0.02 (0) *<33>	-0.34 (0.01) *<100>!#	-0.35 (0.01) *<100>!#	-0.31 (0) *<100>!#	-0.21 (0.01) *<100>!#	-0.1 (0.01) *<100>!#	-0.55 (0.01) *<100>!#	-0.55 (0.01) *<100>!#	-0.52 (0.01) *<100>!#	-0.48 (0.01) *<100>!#	-0.42 (0.01) *<100>!#	-0.74 (0.01) *<100>!#	-0.74 (0.01) *<100>!#	-0.73 (0.01) *<100>!#	-0.72 (0.01) *<100>!#	-0.71 (0.01) *<100>!#	-0.85 (0.01) *<100>!#	-0.85 (0.01) *<100>!#	-0.86 (0) *<100>!#	-0.86 (0) *<100>!#	-0.85 (0.01) *<100>!#

In Table 10, the major criteria for the diagnosis of dysthymic disorder (dys_ma) were the only variable significantly correlated with dysthymic disorder in all simulations (p < 0.001 for all). However, the strengths of correlations depended on the assumed symptom prevalence and correlations, from 0.27 to 1. Other symptoms for the diagnosis of dysthymic disorder were significantly correlated in all simulations, assuming 0.1, 0.4, 0.7, and 0.9 symptom correlations.

In Appendix Tables 11-14, the associations between dysthymic disorder and psychiatric symptoms are presented with correlations or odds ratios. In Appendix Table 12, the symptoms for the diagnosis of major depressive disorder were not significantly associated with dysthymic disorder, assuming no symptom correlations. Three bias variables (mde_bias1, mde_bias2, and mde_bias) were continuous and odds ratios were not available.

In Appendix Table 13, dysthymic disorder and the major and minor criteria usually occurred together and odds ratios were not available. Other symptoms for the diagnosis of dysthymic disorder were not significantly associated with dysthymic disorder based on odds ratios in all simulations, assuming no symptom correlations. One bias variable (dys_mi_bias) was continuous and odds ratios were not available.

In Appendix Table 14, three bias variables (man_bias1, man_bias2, and man_bias) were continuous and odds ratios were not available. The symptoms for the diagnosis of manic episodes were not significantly associated with dysthymic disorder in all simulations under all combinations of symptom prevalence and correlations.

Manic episodes and psychiatric symptoms

Appendix Tables 15-17 present the correlations between manic episodes and the symptoms for the diagnosis of major depressive episodes, dysthymic disorder, and manic episodes, respectively. In Appendix Tables 15, 16, manic episodes are not significantly correlated with the symptoms for the diagnosis of major depressive disorder or dysthymic disorder in all simulations under all combinations of assumed symptom prevalence and correlations. In Appendix Table 17, manic episodes are not significantly correlated with the symptoms for the diagnosis of manic episodes in all simulation, assuming no symptom correlations. The correlations between manic episodes and the symptoms could be very strong or strong (coefficients > 0.85) in the simulations, assuming 0.7 symptom prevalence and 0.9 correlations.

Appendix Table 18 presents the associations between manic episodes and psychiatric symptoms using odds ratios. In Appendix Table 18, manic episodes were not significantly associated with the symptoms for the diagnosis of major depressive episodes or dysthymic disorder in all simulations. Manic episodes were not significantly associated with the symptoms for the diagnosis of manic episodes assuming no symptom correlations. One symptom in the major criteria (man_ma3) often occurred with manic episodes and the odds ratios were not available.

## Discussion

Mental illness diagnoses often are composite diagnostic criteria consisting of psychiatric symptoms [5]. There are several challenges to establishing the causal relationships between psychiatric symptoms and the diagnoses of mental illnesses. First, psychiatric symptoms need to exist or can be observed in patients. However, we did not identify sufficient evidence to show that all of the psychiatric symptoms defined in the DSM (American Psychiatric Association, Philadelphia, PA) could be identified in patients in the real world [5]. There is a lack of epidemiological evidence and studies to systematically determine the prevalence and incidence of all psychiatric symptoms [5]. Second, the diseases that the diagnoses of mental illnesses aim to represent need to exist, too. However, some researchers criticized that not all mental disorders defined by the DSM criteria exist [19]. Again, we could not identify systematic and comprehensive efforts to identify the prevalence and incidence of all mental disorders defined by the DSM criteria. Third, if the disorder does exist, it is important to accurately diagnose the disorder. However, the diagnostic accuracy of symptoms is far from perfect [3]. Although the diagnostic accuracy of symptoms, sensitivity and specificity, can increase gradually when using more symptoms to detect a disease cause, it is unlikely to reach perfect diagnostic accuracy even with more than 30 symptoms [3]. We do not identify evidence to show incremental increases in diagnostic accuracy for a mental illness using more psychiatric symptoms [3].

The fourth one is that the connection between the diagnoses and the psychiatric symptoms used to diagnose them should not be disturbed. Recently, data scientists have focused on how diagnostic criteria modified and distorted the relationships between symptoms and the conditions that the symptoms aimed to diagnose. Using real-world data, the diagnosis of frailty fails to have significant associations with all of the frailty symptoms [20]. Instead, frailty diagnosis is significantly associated with several bias variables, information that could not be explained by input symptoms [20]. The linkage between symptoms and diagnoses can sometimes be disturbed by complicated diagnostic criteria. In this study, our objective is to understand statistical significance of the associations between diagnoses and symptoms. The statistical significance of the associations between diagnoses and symptoms can help to answers questions, including whether the diagnostic criteria have disrupted the connection between psychiatric symptoms and the mental illness diagnoses and whether some of the symptoms became insignificantly associated with the diagnoses, even though they are linked to the diagnoses by the diagnostic criteria.

Causal inference

When both the symptoms and the disease represented by the diagnosis exist, there are several approaches to assess the causal relationships between them. The long-standing Bradford-Hill Criteria [8] for causation has been introduced decades ago, but remains an important concept in major epidemiology textbook [10,21,22]. According to the Bradford-Hill criteria for causation [8], strengths of associations are one of the major elements to establish causation. In the most recent causal inference concepts, significant associations remain an important part of making causal inference [23]. However, we did not identify studies that clearly demonstrated how mental illnesses caused the psychiatric symptoms that are used to diagnose them. In this simulation study to understand the strengths and significance of associations between psychiatric symptoms and diagnoses, three mental conditions have been simulated based on various combinations of symptom prevalence and correlations, assuming similar prevalence and correlations across psychiatric symptoms.

Previous findings suggest diagnostic criteria of mental illnesses have been designed so complicated and weights have been implicitly assigned to psychiatric symptoms [5]. For example, there are three symptoms listed in the major criteria for the diagnosis of manic episodes: elevated, expansive, or irritable mood [5]. Public members that we surveyed might consider these three symptoms similarly important [7]. However, one of the symptoms, “irritable mood,” alone could sometimes explain almost 50% of the variances of the diagnosis of manic episodes [5]. This shows that this symptom has been assigned much more weights for explaining the diagnosis of manic episodes implicitly [5]. Recently data scientists began to recognize that the complicated criteria to diagnose mental illnesses in fact inflated or deflated the importance of psychiatric symptoms for mental illness diagnosis [5]. The importance of some psychiatric symptoms has been reduced to a degree that significant associations with the diagnosis may be lacking.

More specifically, there are several patterns in the associations between psychiatric symptoms and diagnoses. First, three mental illness diagnoses do not significantly correlate with their own symptoms in all simulations assuming similar symptom prevalence and correlation, particularly when symptoms are not correlated. Three exceptions exist. The major criteria for the diagnosis of dysthymic disorder (dys_ma) are significantly correlated with dysthymic disorder in all simulations. Two of the three symptoms in the major criteria for the diagnosis of major depressive episodes (mde_ma1 and mde_ma2) are significantly correlated with major depressive episodes in all simulations. Second, the symptoms for the diagnosis of major depressive episodes and dysthymic disorder are significantly correlated with these two diagnoses in all simulations, assuming 0.1, 0.4, 0.7, or 0.9 symptom correlations, except for one input symptom of dysthymic disorder (dys_mi6). Third, the overlap in the six input symptoms for the diagnosis of major depressive episodes and dysthymic disorder also leads to significant correlations between these two diagnoses, assuming 0.1, 0.4, 0.7, and 0.9 correlations between input symptoms.

Fourth, manic episodes are not significantly associated with the input symptoms of major depressive episodes and dysthymic disorder, assuming these symptoms can be accurately reported and diagnosed. However, previous research shows that symptoms for the diagnosis of major depressive episodes and manic episodes can be equivocal for patients [7]. The patients’ symptoms may be interpreted as symptoms for both major depressive episodes and manic episodes [7]. For example, “distractibility” and “diminished ability to concentrate” are symptoms that are similar to some patients, but are used to diagnose these two opposite conditions: manic episodes and major depressive episodes, respectively [7]. It has been hypothesized that the diagnosis of bipolar disorder, which is established upon the diagnosis of manic episodes and major depressive episodes in the same individuals, could be due to the design of the diagnostic criteria that failed to provide clearly-defined symptoms for opposite conditions [7]. Lastly, it is rare to observe an absolute lack of significant correlations between the three diagnoses among 100 simulations. In populations with symptoms of similar correlations occurring at random, the design of the diagnostic criteria leads to cases confirmed with these three diagnoses in some of the simulations. In addition, there are confirmed cases with both major depressive episodes and manic episodes or both dysthymic disorder and manic episodes among 100 simulations, even when manic symptoms are highly correlated with each other and unrelated to those for the diagnosis of major depressive episodes and dysthymic disorder. The diagnostic criteria of manic episodes may not have been designed to sufficiently distinguish patients with manic episodes from those with major depressive episodes or dysthymic disorder. A recent study also identified that there is an overlap in bipolar symptoms, borderline features during depression [24], while another considered the overlap in symptoms can be found across mental illness diagnoses [25].

Why mental illness diagnoses fail to cause their symptoms?

Moreover, these findings suggest that there are situations, in which the causal relationships between mental illness diagnoses and psychiatric symptoms cannot be confirmed due to the lack of significant associations between psychiatric symptoms and the diagnoses that these symptoms aim to confirm. There are several reasons to this issue, including bias variables created by data processing steps in composite diagnostic criteria [5], a lack of biological evidence base for most diagnoses [26], and the arbitrary nature of the diagnostic criteria [25]. Researchers screened the symptoms used for various diagnoses of mental illnesses and found many symptoms were interpreted differently by health professionals and the members of the public [7]. Data scientists have begun to recognize that composite diagnostic criteria are algorithms to aggregate information on input symptoms [5,27].

In detail, several inappropriate data processing methods to aggregate information directly induce biases to the derived variables or diagnoses [5,27]. For example, when input variables are summed into a continuous variable, this derived variable can be censored to derive new binomial variables depending on the thresholds. When data censoring occurs, the derived variables could not fully be explained by input variables because some information is discarded [27,28]. Instead, the derived binomial variables is, in fact, the sum of original input variables and a bias variable [5,27]. In the simulations, nine, six, and seven bias variables are required to create the diagnoses of major depressive episodes, dysthymic disorder, and manic episodes, respectively [5]. These bias variables introduce information that cannot be explained by the input symptoms and thus influence the relationships between mental illness diagnoses and their symptoms [5].

The diagnostic criteria for mental illnesses lack evidence support [7] to establish the causation between mental illnesses and symptoms used to diagnose them. Some researchers consider that the validity of the DSM diagnostic criteria has not been well established [26]. Other researchers reviewed the diagnostic criteria in the DSM-5 and found several issues in the DSM criteria: heterogenous decision-making rules for diagnoses, overlaps in the symptoms used to diagnose various conditions, neglecting the role of trauma or adverse events in the diagnoses, and the disconnect between diagnoses and treatment options [29]. This leads to a conclusion that mental illness diagnostic criteria are scientifically meaningless [29]. In addition, the underlying theories of the diagnostic system remains contentious [25].

Without proper theoretical or pathological foundations to the diagnostic criteria, we found the assumption that various degrees of correlations between input symptoms can coexist very difficult to justify. For example, there are pairs of symptoms used to created intermediate variables, including “sleep too much” and “insomnia”, “weight gain” and “weight loss”, and “agitation” and “psychomotor retardation” that are pairs of symptoms unlikely to occur at the same time in the same individuals, i.e., uncorrelated symptoms, for the diagnosis of major depressive episodes. In contrast, two pairs of symptoms, “fatigue” and “loss of energy” and “diminished ability to think or concentrate” and “indecisiveness”, are for the diagnosis of major depressive episodes and can be used to describe the same reaction to life events, i.e., correlated symptoms. The correlations between symptoms are related to the magnitudes of biases introduced to the desired diagnosis [5,27]. Biases are certain to occur from these pairs of symptoms [5], but the magnitudes of these biases are uncertain due to the lack of real-world data that clearly demonstrate the correlations between symptoms. In this study, we confirmed the overlap in the symptoms for the diagnosis of major depressive episodes and dysthymic disorder [5] and the overlap is related to the significant correlations between these two diagnoses. We have not identified evidence to show that DSM intends to have these two diagnoses correlated and how much these two diagnoses should be correlated.

In the simulations assuming similar prevalence and correlations across all symptoms, symptom prevalence and correlations are two factors that determine the strengths of associations between mental illnesses and their symptoms. To our knowledge, we did not identify sufficient epidemiological evidence that shows the role of symptoms prevalence or correlations in their associations with the diagnosis of mental illnesses [5]. In addition, the presence of opportunistically significant correlations under almost all combinations of symptoms prevalence and correlations might suggest the issue of the lack of specificity (ability to identify those not diseased) embedded in composite diagnostic criteria [27].

Implications for diagnosing mental illnesses

The composite criteria used to diagnose mental illnesses can be improved in several directions according to recently published recommendations [30]. First, bias variables can be avoided using systematic methods to mine innovative indices [27] or syndromes [31]. Without bias variables introduced by composite diagnostic criteria, derived variables can be well interpreted with input symptoms and the importance of the input symptoms toward various outcomes can be easily assessed [31].

Second, psychiatric symptoms may be normal responses to life events, but psychiatric symptoms cause concern when they inflict stress or affect quality of life according to the American Psychiatric Association, the publisher of DSM [32]. This means psychiatric symptoms are not problems themselves and what is looked for is a proxy measure for the levels of stress or changes in quality of life. This implies that diagnoses of mental illnesses should be accurate measures for the deterioration in quality of life caused by these symptoms, rather than indicators of pathological progress. Proxy measures of this kind can be identified using syndrome mining techniques [31]. These techniques identify co-occurrence of multiple psychiatric symptoms to form diagnostic criteria [31]. For example, in studies on frailty, frail symptoms have been selected based on theories and any four symptoms have been summed to create candidate alternative frailty syndromes [31]. These candidates have been screened based on their accuracy for mortality prediction [31]. These steps can be replicated to mine mental illness diagnoses. However, there are several issues that need to be defined and clarified a priori, for example, what mental illness diagnoses are meant to measure, how to select symptoms pertinent to a particular diagnosis, and what are the outcomes to assess the diagnostic accuracy of these diagnoses. In the simulations, the diagnostic criteria of manic episodes can be refined to improve their specificity. The various degrees of correlations between major depressive episodes and dysthymic disorder are suggesting both diagnoses may not be able to clearly separate two groups of patients (i.e., insufficient diagnostic accuracy). Before mining mental syndromes, these issues need to be explored and properly addressed.

Third, recent evidence suggests that the use of composite diagnostic criteria of inferior interpretability, i.e., frailty indices, may be associated with early trial termination or failure [33]. For mental health researchers, it is important not only to ensure the conditions in their trials aligned with the Research Domain Criteria well [34], but also to assess the interpretability of the diagnoses. When diagnoses of poor interpretability are used for treatment decisions, populations of distinct survival patterns can be regarded the same and treated similarly [6]. The diagnostic criteria of major depressive episodes, dysthymic disorder, and manic episodes are subject to biases and cannot be well interpreted with their own input symptoms [5]. Using symptoms as outcomes or applying interpretable diagnoses in trials may help trials not to terminate early.

Fourth, there is a potential mismatch between composite diagnostic criteria and interventions. For example, having two or more deficits can be diagnosed with frailty [27]. Nutritive and physical deficits are two of the four deficits, but not all frail patients have these two deficits [27]. However, nutrition and exercise interventions may be assigned to all frail patients regardless of their nutritive or physical statuses. Applying interventions to patients identified by composite diagnostic criteria can introduce unnecessary interventions to participants and have major ethical and clinical implications. It is recommended to understand how the diagnoses align with the interventions in trials.

Lastly, we expect to identify new symptoms from real-world data, such as posts at social media and unstructured texts. The DSM system dictates the methods that we can use to identify psychiatric symptoms for diagnosis, i.e., by “professional” assessment. Public and patient engagement has been an important and essential part of current approaches to assess health technologies [35]. However, the public or patients are not actively engaged in the Working Groups to form DSM diagnoses [36]. This obviously neglects numerous symptoms that are waiting for us to identify, screen, be used for syndrome mining, and adopt for making diagnoses.

Based on the latest guideline on mining innovative composite measures [30] and the recent introduction of the perspectives of patients or the public [7], we think it possible to improve the diagnostic criteria and recommend researchers to collect epidemiological evidence (prevalence and incidence of psychiatric symptoms or preferably biomarker data), assess the relationships between symptoms by consulting patients or members of the public [7], create interpretable diagnostic criteria based on the literature [30], avoid data processing steps that introduce biases to the diagnoses [27], verify the diagnostic accuracy of symptoms for well-defined diagnoses [3], and examine the likelihood of falsely defining cases in populations with near-zero prevalence [27]. When researchers are confident the identified psychiatric symptoms can be sensitive to cases and be specific enough to exclude those who are not likely to have the condition, the newly developed diagnostic criteria will become less vulnerable to the critics that the currently used DSM system is subject to.

Limitations

A limitation to causal inference using observational data in the real world is confounding [23]. In patients, there are other factors that modify the relationships between psychiatric symptoms and diagnoses, such as genetic predisposition, biological, environmental, and social factors [37]. This simulation study eliminated all confounders and the only link between psychiatric symptoms and diagnoses is the diagnostic criteria. The insignificant associations between symptoms and diagnoses identified in this study could not be attributed to confounders. Although making causal inference is difficult, this simulation study helps to eliminate most of the limitation related to confounding.

Even though this simulation study is built on the reproducible R codes and verified assumptions in a publication freely accessible online [5]. There are other limitations to this study. The assumptions, including similar prevalence and correlations across symptoms, might not be realistic in the real world [5]. There are other measures of associations, such as risk ratios and odds ratios, to assess the strengths of associations [10]. These measures may be pertinent to other study designs that this simulation study would not fit properly. We could not simulate symptoms with negative correlations [38]. However, negative correlations may occur between several pairs of symptoms, such as the relationships between “insomnia” and “sleep too much” or “unintentional weight gain” and “unintentional weight loss” for the diagnosis of major depressive episodes or “poor appetite” and “overeating” for the diagnosis of dysthymic disorder. Negative correlations between symptoms may cause certain symptoms to occur less often among those diagnosed and provide a better illustration for the lack of causal relationship between symptoms and diagnoses.

Reporting on the level of statistical significance, p-values, is also a limitation. Because there are 100 simulations for each of the 25 combinations of symptom prevalence and correlation, it was not possible to list and summarize all confidence intervals. We chose to summarize the p values from 100 simulations. For the same reason, it was not possible to report all of the effect sizes of all symptoms for predicting the diagnoses in all simulations. Effect sizes could be useful for clinicians to understand the potential roles of symptoms on diagnoses. However, reporting effect sizes would increase more burden for readers to understand the results. Readers are encouraged to adopt the R codes and observe the confidence intervals and effect sizes.

Lastly, odds ratios are not applicable when 0 values appear in the denominators for odds ratio calculation. Several psychiatric symptoms are highly correlated with the diagnoses these symptoms aim to diagnose, including the major and minor criteria for major depressive episodes and dysthymic disorder, one symptom for manic episodes (man_ma3). In cases where there are insufficient numbers of patients, odds ratios also are not available, including major depressive episodes assuming 0.05 symptom prevalence and no correlations.

## Conclusions

There are several challenges to establishing the causation between mental illness diagnoses and psychiatric symptoms used to diagnose them. To our knowledge, there are no systematic and comprehensive efforts to assess the prevalence and incidence of psychiatric symptoms or mental illness diagnoses identified by the DSM and demonstrate whether both can be observed in patients. The diagnostic accuracy, including sensitivity and specificity, of symptoms is far from perfect for detecting the disease causes. Assuming the psychiatric symptoms of three mood disorders exist and can be observed in patients, the causal relationship between them has never been assessed. According to several theories of causation commonly used in health research, significant associations are one important factor in establishing causation.

In this simulation study, we found that three mental illness diagnoses were not significantly correlated with their own symptoms in all simulations, particularly when symptoms were not correlated, except for the symptom in the major criteria of major depressive episodes or dysthymic disorder. The symptoms for the diagnosis of major depressive episodes and dysthymic disorder were significantly correlated with these two diagnoses in some simulations, assuming 0.1, 0.4, 0.7, or 0.9 symptom correlations, except for one symptom. The overlap in the input symptoms for the diagnosis of major depressive episodes and dysthymic disorder also leads to significant correlations between these two diagnoses, assuming 0.1, 0.4, 0.7, and 0.9 correlations between input symptoms. Manic episodes are not significantly associated with the input symptoms of major depressive episodes and dysthymic disorder.

 The diagnostic criteria of major depressive episodes, dysthymic disorder, and manic episodes have not been designed to guarantee significant associations between symptoms and diagnoses. The lack of causation between diagnoses and symptoms is related to several issues. Bias variables have been induced by the composite diagnostic criteria of the three diagnoses and influence the relationships between diagnoses and symptoms. The evidence to support this diagnostic approach does not seem sufficient. The theoretical bases for mental illness diagnoses remain contentious and the principles for selecting psychiatric symptoms are heterogeneous across diagnoses. The epidemiological evidence to support the causal relationships between diagnoses and their symptoms seems to be lacking. The diagnostic criteria for mental illnesses can be refined to avoid biases, improve interpretability, and better measure patients’ well-being.

## Appendices

**Table 11:** Correlations between dysthymic disorder and the input symptoms of manic episodes by assumed symptom prevalence and correlations Mean (standard deviation) *<number> = the numbers of significant statistics (p < 0.05) in 100 simulations !# = p < 0.001 in all of the 100 simulations !@ = p < 0.001 in some of the 100 simulations manic = Manic episodes; man_ma1 = Elevated mood, lasting at least 1 week; man_ma2 = Expansive mood, lasting at least 1 week; man_ma3 = Irritable mood, lasting at least 1 week; man_mi1 = Increased self-esteem or grandiosity; man_mi1_1 = Increased self-esteem; man_mi1_2 = Grandiosity; man_mi1_bias = Information of the domain not explained by the input variables; man_mi2 = Decreased need for sleep (e.g., feels rested after only 3 hours of sleep); man_mi3 = More talkative than usual or pressure to keep talking; man_mi3_1 = More talkative than usual; man_mi3_2 = Pressure to keep talking; man_mi3_bias = Information of the domain not explained by the input variables; man_mi4 = Flight of ideas or subjective experience that thoughts are racing; man_mi4_1 = Flight of ideas; man_mi4_2 = Subjective experience that thoughts are racing; man_mi4_bias = Information of the domain not explained by the input variables; man_mi5 = Distractibility (i.e., attention too easily drawn to unimportant or irrelevant external stimuli); man_mi6 = Increase in goal-directed activity (either socially, at work or school, or sexually) or psychomotor agitation; man_mi6_1 = Increase in goal-directed activity ; man_mi6_2 = Psychomotor agitation; man_mi6_bias = Information of the domain not explained by the input variables; man_mi7 = Excessive involvement in pleasurable activities that have a high potential for painful consequences (e.g., engaging in unrestrained buying sprees, sexual indiscretions, or foolish business investments); man_bias1 = Information of diagnosis due to top-censoring for choosing at least three symptoms; man_bias2 = Information of diagnosis due to top-censoring for choosing at least four symptoms; man_bias = Information of diagnosis not explained by symptoms

Assumed symptom prevalence	0.05	0.1	0.3	0.5	0.7	0.05	0.1	0.3	0.5	0.7	0.05	0.1	0.3	0.5	0.7	0.05	0.1	0.3	0.5	0.7	0.05	0.1	0.3	0.5	0.7
Assumed symptom correlations	0	0	0	0	0	0.1	0.1	0.1	0.1	0.1	0.4	0.4	0.4	0.4	0.4	0.7	0.7	0.7	0.7	0.7	0.9	0.9	0.9	0.9	0.9
manic	0 (0)	0 (0.01) *<4>	0 (0.01) *<2>	0 (0.01) *<2>	0 (0.01) *<6>	0 (0.01) *<1>	0 (0.01) *<2>	0 (0.01) *<4>	0 (0.01) *<7>	0 (0.01) *<5>	0 (0.01) *<6>	0 (0.01) *<4>	0 (0.01) *<12>	0 (0.01) *<5>	0 (0.01) *<6>	0 (0.01) *<2>	0 (0.01) *<6>	0 (0.01) *<6>	0 (0.01) *<9>	0 (0.01) *<6>	0 (0.01) *<4>	0 (0.01) *<4>	0 (0.01) *<3>	0 (0.01) *<3>	0 (0.01) *<3>
man_ma1	0 (0.01) *<1>	0 (0.01) *<6>	0 (0.01) *<5>	0 (0.01) *<6>	0 (0.01) *<4>	0 (0.01) *<5>	0 (0.01) *<7>	0 (0.01) *<10>	0 (0.01) *<5>	0 (0.01) *<4>	0 (0.01) *<8>	0 (0.01) *<3>	0 (0.01) *<8>	0 (0.01) *<2>	0 (0.01) *<3>	0 (0.01) *<2>	0 (0.01) *<5>	0 (0.01) *<6>	0 (0.01) *<7>	0 (0.01) *<5>	0 (0.01) *<3>	0 (0.01) *<5>	0 (0.01) *<1>	0 (0.01) *<2>	0 (0.01) *<3>
man_ma2	0 (0.01) *<7>	0 (0.01) *<8>	0 (0.01) *<3>	0 (0.01) *<6>	0 (0.01) *<8>	0 (0.01) *<5>	0 (0.01) *<5>	0 (0.01) *<4>	0 (0.01) *<10>	0 (0.01) *<4>	0 (0.01) *<8>	0 (0.01) *<3>	0 (0.01) *<9>	0 (0.01) *<6>	0 (0.01) *<5>	0 (0.01) *<3>	0 (0.01) *<6>	0 (0.01) *<6>	0 (0.01) *<6>	0 (0.01) *<3>	0 (0.01) *<6>	0 (0.01) *<7>	0 (0.01) *<4>	0 (0.01) *<3>	0 (0.01) *<2>
man_ma3	0 (0.01) *<4>	0 (0.01) *<2>	0 (0.01) *<4>	0 (0.01) *<4>	0 (0.01) *<8>	0 (0.01) *<2>	0 (0.01) *<3>	0 (0.01) *<3>	0 (0.01) *<5>	0 (0.01) *<3>	0 (0.01) *<3>	0 (0.01) *<9>	0 (0.01) *<10>	0 (0.01) *<2>	0 (0.01) *<6>	0 (0.01) *<4>	0 (0.01) *<8>	0 (0.01) *<5>	0 (0.01) *<9>	0 (0.01) *<2>	0 (0.01) *<5>	0 (0.01) *<4>	0 (0.01) *<5>	0 (0.01) *<4>	0 (0.01) *<4>
man_mi1	0 (0.01) *<5>	0 (0.01) *<4>	0 (0.01) *<4>	0 (0.01) *<3>	0 (0.01) *<4>	0 (0.01) *<5>	0 (0.01) *<4>	0 (0.01) *<7>	0 (0.01) *<2>	0 (0.01) *<6>	0 (0.01) *<5>	0 (0.01) *<6>	0 (0.01) *<9>	0 (0.01) *<1>	0 (0.01) *<1>	0 (0.01) *<1>	0 (0.01) *<7>	0 (0.01) *<5>	0 (0.01) *<5>	0 (0.01) *<8>	0 (0.01) *<5>	0 (0.01) *<5>	0 (0.01) *<2>	0 (0.01) *<1>	0 (0.01) *<3>
man_mi1_1	0 (0.01) *<3>	0 (0.01) *<3>	0 (0.01) *<3>	0 (0.01) *<4>	0 (0.01) *<7>	0 (0.01) *<5>	0 (0.01) *<5>	0 (0.01) *<6>	0 (0.01) *<6>	0 (0.01) *<5>	0 (0.01) *<3>	0 (0.01) *<3>	0 (0.01) *<10>	0 (0.01) *<6>	0 (0.01) *<5>	0 (0.01)	0 (0.01) *<9>	0 (0.01) *<3>	0 (0.01) *<9>	0 (0.01) *<12>	0 (0.01) *<3>	0 (0.01) *<7>	0 (0.01) *<2>	0 (0.01) *<4>	0 (0.01) *<2>
man_mi1_2	0 (0.01) *<2>	0 (0.01) *<4>	0 (0.01) *<5>	0 (0.01) *<2>	0 (0.01) *<3>	0 (0.01) *<6>	0 (0.01) *<5>	0 (0.01) *<5>	0 (0.01) *<5>	0 (0.01) *<6>	0 (0.01) *<7>	0 (0.01) *<6>	0 (0.01) *<4>	0 (0.01) *<2>	0 (0.01) *<2>	0 (0.01) *<6>	0 (0.01) *<8>	0 (0.01) *<3>	0 (0.01) *<7>	0 (0.01) *<9>	0 (0.01) *<4>	0 (0.01) *<5>	0 (0.01) *<2>	0 (0.01) *<2>	0 (0.01) *<3>
man_mi1_bias	0 (0.01) *<12>	0 (0.01) *<4>	0 (0.01) *<5>	0 (0.01) *<5>	0 (0.01) *<3>	0 (0.01) *<2>	0 (0.01) *<5>	0 (0.01) *<4>	0 (0.01) *<8>	0 (0.01) *<4>	0 (0.01) *<3>	0 (0.01) *<7>	0 (0.01) *<6>	0 (0.01) *<4>	0 (0.01) *<3>	0 (0.01) *<6>	0 (0.01) *<7>	0 (0.01) *<4>	0 (0.01) *<9>	0 (0.01) *<7>	0 (0.01) *<4>	0 (0.01) *<3>	0 (0.01) *<3>	0 (0.01) *<1>	0 (0.01) *<3>
man_mi2	0 (0.01) *<2>	0 (0.01) *<9>	0 (0.01) *<4>	0 (0.01) *<6>	0 (0.01) *<3>	0 (0.01) *<5>	0 (0.01) *<7>	0 (0.01) *<6>	0 (0.01) *<9>	0 (0.01) *<1>	0 (0.01) *<5>	0 (0.01) *<4>	0 (0.01) *<7>	0 (0.01) *<1>	0 (0.01) *<7>	0 (0.01) *<3>	0 (0.01) *<6>	0 (0.01) *<5>	0 (0.01) *<4>	0 (0.01) *<6>	0 (0.01) *<6>	0 (0.01) *<7>	0 (0.01) *<3>	0 (0.01) *<3>	0 (0.01) *<2>
man_mi3	0 (0.01) *<3>	0 (0.01) *<3>	0 (0.01) *<5>	0 (0.01) *<6>	0 (0.01) *<4>	0 (0.01) *<2>	0 (0.01) *<4>	0 (0.01) *<5>	0 (0.01) *<5>	0 (0.01) *<3>	0 (0.01) *<5>	0 (0.01) *<6>	0 (0.01) *<13>	0 (0.01) *<9>	0 (0.01) *<6>	0 (0.01) *<3>	0 (0.01) *<7>	0 (0.01) *<7>	0 (0.01) *<2>	0 (0.01) *<9>	0 (0.01) *<7>	0 (0.01) *<3>	0 (0.01) *<2>	0 (0.01) *<2>	0 (0.01) *<2>
man_mi3_1	0 (0.01) *<3>	0 (0.01) *<5>	0 (0.01) *<8>	0 (0.01) *<6>	0 (0.01) *<5>	0 (0.01) *<1>	0 (0.01) *<6>	0 (0.01) *<8>	0 (0.01) *<4>	0 (0.01) *<5>	0 (0.01) *<5>	0 (0.01) *<4>	0 (0.01) *<4>	0 (0.01) *<8>	0 (0.01) *<5>	0 (0.01) *<1>	0 (0.01) *<5>	0 (0.01) *<5>	0 (0.01) *<8>	0 (0.01) *<7>	0 (0.01) *<8>	0 (0.01) *<4>	0 (0.01) *<3>	0 (0.01) *<5>	0 (0.01) *<1>
man_mi3_2	0 (0.01) *<3>	0 (0.01) *<5>	0 (0.01) *<2>	0 (0.01) *<8>	0 (0.01) *<4>	0 (0.01) *<3>	0 (0.01) *<5>	0 (0.01) *<11>	0 (0.01) *<5>	0 (0.01) *<5>	0 (0.01) *<3>	0 (0.01) *<3>	0 (0.01) *<11>	0 (0.01) *<5>	0 (0.01) *<8>	0 (0.01) *<5>	0 (0.01) *<7>	0 (0.01) *<5>	0 (0.01) *<5>	0 (0.01) *<3>	0 (0.01) *<6>	0 (0.01) *<4>	0 (0.01) *<1>	0 (0.01) *<4>	0 (0.01) *<4>
man_mi3_bias	0 (0.01) *<12>	0 (0.01) *<5>	0 (0.01) *<6>	0 (0.01) *<10>	0 (0.01) *<7>	0 (0.01) *<3>	0 (0.01) *<6>	0 (0.01) *<8>	0 (0.01) *<4>	0 (0.01) *<7>	0 (0.01) *<8>	0 (0.01) *<4>	0 (0.01) *<6>	0 (0.01) *<4>	0 (0.01) *<5>	0 (0.01) *<5>	0 (0.01) *<8>	0 (0.01) *<5>	0 (0.01) *<6>	0 (0.01) *<7>	0 (0.01) *<5>	0 (0.01) *<4>	0 (0.01) *<4>	0 (0.01) *<5>	0 (0.01) *<4>
man_mi4	0 (0.01) *<3>	0 (0.01) *<6>	0 (0.01) *<5>	0 (0.01) *<6>	0 (0.01) *<3>	0 (0.01) *<5>	0 (0.01) *<6>	0 (0.01) *<6>	0 (0.01) *<7>	0 (0.01) *<7>	0 (0.01) *<2>	0 (0.01) *<5>	0 (0.01) *<3>	0 (0.01) *<8>	0 (0.01) *<4>	0 (0.01) *<7>	0 (0.01) *<4>	0 (0.01) *<4>	0 (0.01) *<6>	0 (0.01) *<7>	0 (0.01) *<4>	0 (0.01) *<4>	0 (0.01) *<2>	0 (0.01) *<3>	0 (0.01) *<6>
man_mi4_1	0 (0.01) *<3>	0 (0.01) *<3>	0 (0.01) *<3>	0 (0.01) *<8>	0 (0.01) *<9>	0 (0.01) *<3>	0 (0.01) *<7>	0 (0.01) *<2>	0 (0.01) *<2>	0 (0.01) *<1>	0 (0.01) *<4>	0 (0.01) *<6>	0 (0.01) *<7>	0 (0.01) *<4>	0 (0.01) *<2>	0 (0.01) *<4>	0 (0.01) *<5>	0 (0.01) *<5>	0 (0.01) *<6>	0 (0.01) *<2>	0 (0.01) *<4>	0 (0.01) *<6>	0 (0.01) *<3>	0 (0.01) *<2>	0 (0.01) *<4>
man_mi4_2	0 (0.01) *<2>	0 (0.01) *<4>	0 (0.01) *<5>	0 (0.01) *<5>	0 (0.01) *<4>	0 (0.01) *<1>	0 (0.01) *<6>	0 (0.01) *<2>	0 (0.01) *<6>	0 (0.01) *<7>	0 (0.01) *<8>	0 (0.01) *<6>	0 (0.01) *<4>	0 (0.01) *<6>	0 (0.01) *<6>	0 (0.01) *<5>	0 (0.01) *<3>	0 (0.01) *<3>	0 (0.01) *<4>	0 (0.01) *<7>	0 (0.01) *<7>	0 (0.01) *<5>	0 (0.01) *<2>	0 (0.01) *<4>	0 (0.01) *<2>
man_mi4_bias	0 (0.01) *<13>	0 (0.01) *<4>	0 (0.01) *<3>	0 (0.01) *<5>	0 (0.01) *<5>	0 (0.01) *<5>	0 (0.01) *<7>	0 (0.01) *<4>	0 (0.01) *<5>	0 (0.01) *<6>	0 (0.01) *<7>	0 (0.01) *<8>	0 (0.01) *<5>	0 (0.01) *<3>	0 (0.01) *<6>	0 (0.01) *<6>	0 (0.01) *<3>	0 (0.01) *<2>	0 (0.01) *<7>	0 (0.01) *<5>	0 (0.01) *<9>	0 (0.01) *<7>	0 (0.01) *<2>	0 (0.01) *<3>	0 (0.01) *<3>
man_mi5	0 (0.01) *<4>	0 (0.01) *<3>	0 (0.01) *<6>	0 (0.01) *<2>	0 (0.01) *<7>	0 (0.01) *<6>	0 (0.01) *<3>	0 (0.01) *<2>	0 (0.01) *<7>	0 (0.01) *<3>	0 (0.01) *<4>	0 (0.01) *<2>	0 (0.01) *<6>	0 (0.01) *<5>	0 (0.01) *<3>	0 (0.01) *<4>	0 (0.01) *<4>	0 (0.01) *<5>	0 (0.01) *<9>	0 (0.01) *<8>	0 (0.01) *<8>	0 (0.01) *<5>	0 (0.01) *<4>	0 (0.01) *<3>	0 (0.01) *<5>
man_mi6	0 (0.01) *<3>	0 (0.01) *<6>	0 (0.01) *<8>	0 (0.01) *<6>	0 (0.01) *<7>	0 (0.01) *<5>	0 (0.01) *<3>	0 (0.01) *<8>	0 (0.01) *<4>	0 (0.01) *<6>	0 (0.01) *<5>	0 (0.01) *<7>	0 (0.01) *<10>	0 (0.01) *<8>	0 (0.01) *<4>	0 (0.01) *<3>	0 (0.01) *<8>	0 (0.01) *<4>	0 (0.01) *<2>	0 (0.01) *<4>	0 (0.01) *<6>	0 (0.01) *<5>	0 (0.01) *<4>	0 (0.01) *<2>	0 (0.01) *<3>
man_mi6_1	0 (0.01) *<3>	0 (0.01) *<5>	0 (0.01) *<7>	0 (0.01) *<6>	0 (0.01) *<2>	0 (0.01) *<6>	0 (0.01) *<5>	0 (0.01) *<9>	0 (0.01) *<8>	0 (0.01) *<5>	0 (0.01) *<2>	0 (0.01) *<7>	0 (0.01) *<7>	0 (0.01) *<7>	0 (0.01) *<7>	0 (0.01) *<6>	0 (0.01) *<5>	0 (0.01) *<4>	0 (0.01) *<6>	0 (0.01) *<7>	0 (0.01) *<7>	0 (0.01) *<6>	0 (0.01) *<2>	0 (0.01) *<4>	0 (0.01) *<4>
man_mi6_2	0 (0.01) *<6>	0 (0.01) *<7>	0 (0.01) *<8>	0 (0.01) *<4>	0 (0.01) *<3>	0 (0.01) *<4>	0 (0.01) *<1>	0 (0.01) *<7>	0 (0.01) *<5>	0 (0.01) *<7>	0 (0.01) *<6>	0 (0.01) *<7>	0 (0.01) *<12>	0 (0.01) *<2>	0 (0.01) *<6>	0 (0.01) *<5>	0 (0.01) *<6>	0 (0.01) *<4>	0 (0.01) *<6>	0 (0.01) *<4>	0 (0.01) *<8>	0 (0.01) *<4>	0 (0.01) *<4>	0 (0.01) *<2>	0 (0.01) *<3>
man_mi6_bias	0 (0.01) *<9>	0 (0.01) *<3>	0 (0.01) *<5>	0 (0.01) *<5>	0 (0.01) *<7>	0 (0.01) *<1>	0 (0.01) *<6>	0 (0.01) *<7>	0 (0.01) *<1>	0 (0.01) *<5>	0 (0.01) *<9>	0 (0.01) *<8>	0 (0.01) *<8>	0 (0.01) *<4>	0 (0.01) *<6>	0 (0.01) *<8>	0 (0.01) *<3>	0 (0.01) *<5>	0 (0.01) *<7>	0 (0.01) *<6>	0 (0.01) *<7>	0 (0.01) *<5>	0 (0.01) *<4>	0 (0.01) *<5>	0 (0.01) *<5>
man_mi7	0 (0.01) *<5>	0 (0.01) *<3>	0 (0.01) *<3>	0 (0.01) *<4>	0 (0.01) *<6>	0 (0.01) *<2>	0 (0.01) *<3>	0 (0.01) *<2>	0 (0.01) *<2>	0 (0.01) *<3>	0 (0.01) *<5>	0 (0.01) *<6>	0 (0.01) *<5>	0 (0.01) *<7>	0 (0.01) *<5>	0 (0.01) *<4>	0 (0.01) *<4>	0 (0.01) *<3>	0 (0.01) *<7>	0 (0.01) *<9>	0 (0.01) *<9>	0 (0.01) *<4>	0 (0.01) *<4>	0 (0.01) *<4>	0 (0.01) *<3>
man_bias1	0 (0.01) *<4>	0 (0.01) *<4>	0 (0.01) *<4>	0 (0.01) *<6>	0 (0.01) *<8>	0 (0.01) *<3>	0 (0.01) *<5>	0 (0.01) *<4>	0 (0.01) *<7>	0 (0.01) *<7>	0 (0.01) *<3>	0 (0.01) *<4>	0 (0.01) *<11>	0 (0.01) *<4>	0 (0.01) *<4>	0 (0.01) *<2>	0 (0.01) *<6>	0 (0.01) *<4>	0 (0.01) *<6>	0 (0.01) *<9>	0 (0.01) *<8>	0 (0.01) *<4>	0 (0.01) *<2>	0 (0.01) *<3>	0 (0.01) *<2>
man_bias2	0 (0.01) *<5>	0 (0.01) *<4>	0 (0.01) *<2>	0 (0.01) *<7>	0 (0.01) *<7>	0 (0.01) *<5>	0 (0.01) *<4>	0 (0.01) *<5>	0 (0.01) *<6>	0 (0.01) *<8>	0 (0.01) *<3>	0 (0.01) *<5>	0 (0.01) *<11>	0 (0.01) *<4>	0 (0.01) *<5>	0 (0.01) *<2>	0 (0.01) *<6>	0 (0.01) *<4>	0 (0.01) *<6>	0 (0.01) *<9>	0 (0.01) *<7>	0 (0.01) *<4>	0 (0.01) *<2>	0 (0.01) *<3>	0 (0.01) *<4>
man_bias	0 (0.01) *<6>	0 (0.01) *<2>	0 (0.01) *<3>	0 (0.01) *<6>	0 (0.01) *<5>	0 (0.01) *<3>	0 (0.01) *<7>	0 (0.01) *<7>	0 (0.01) *<2>	0 (0.01) *<8>	0 (0.01) *<1>	0 (0.01) *<7>	0 (0.01) *<10>	0 (0.01)	0 (0.01) *<5>	0 (0.01) *<5>	0 (0.01) *<4>	0 (0.01) *<5>	0 (0.01) *<6>	0 (0.01) *<7>	0 (0.01) *<7>	0 (0.01) *<4>	0 (0.01) *<4>	0 (0.01) *<4>	0 (0.01) *<3>

**Table 12:** Odds ratios between dysthymic disorder and the input symptoms of major depressive episodes by assumed symptom prevalence and correlations. NA = not applicable due to 0 case in the denominators for the calculation of odds ratios Mean (standard deviation) *<number> = the numbers of significant statistics (p < 0.05) in 100 simulations !# = p < 0.001 in all of the 100 simulations !@ = p < 0.001 in some of the 100 simulations mde = Major Depressive Episodes; mde_ma1 = Depressed mood for more than two weeks.; mde_ma2 = Loss of interest or pleasure in daily activities for more than two weeks.; mde_mi3 = Significant unintentional weight loss or gain; mde_mi3_1 = Significant unintentional weight gain; mde_mi3_2 = Significant unintentional weight loss; mde_mi3_bias = Information of the domain not explained by the input variables; mde_mi4 = Insomnia or sleeping too much; mde_mi4_1 = Insomnia; mde_mi4_2 = Sleeping too much; mde_mi4_bias = Information of the domain not explained by the input variables; mde_mi5 = Agitation or psychomotor retardation noticed by others; mde_mi5_1 = Agitation; mde_mi5_2 = Psychomotor retardation noticed by others; mde_mi5_bias = Information of the domain not explained by the input variables; mde_mi6 = Fatigue or loss of energy; mde_mi6_1 = Fatigue; mde_mi6_2 = Loss of energy; mde_mi6_bias = Information of the domain not explained by the input variables; mde_mi7 = Feelings of worthlessness or excessive guilt; mde_mi7_1 = Feelings of worthlessness; mde_mi7_2 = Feelings of excessive guilt; mde_mi7_bias = Information of the domain not explained by the input variables; mde_mi8 = Diminished ability to think or concentrate, or indecisiveness+; mde_mi8_1 = Diminished ability to think or concentrate; mde_mi8_2 = Indecisiveness; mde_mi8_bias = Information of the domain not explained by the input variables; mde_mi9 = Recurrent thoughts of death; mde_bias1 = Information due to top censoring by choosing three domains in minor criteria; mde_bias2 = Information due to top censoring by choosing four domains in minor criteria; mde_bias = Information of diagnosis not explained by the domain

Assumed symptom prevalence	0.05	0.1	0.3	0.5	0.7	0.05	0.1	0.3	0.5	0.7	0.05	0.1	0.3	0.5	0.7	0.05	0.1	0.3	0.5	0.7	0.05	0.1	0.3	0.5	0.7
Assumed symptom correlations	0	0	0	0	0	0.1	0.1	0.1	0.1	0.1	0.4	0.4	0.4	0.4	0.4	0.7	0.7	0.7	0.7	0.7	0.9	0.9	0.9	0.9	0.9
mde	NA	2.48 (3.75) *<8>	1.11 (0.12) *<27>	1 (0.05) *<7>	1 (0.04) *<3>	27 (10.7) *<100>!@	10.07 (1.97) *<100>!#	2.65 (0.18) *<100>!#	1.88 (0.08) *<100>!#	1.76 (0.07) *<100>!#	78.67 (13.07) *<100>!#	36.46 (4.02) *<100>!#	12.25 (0.82) *<100>!#	8.72 (0.39) *<100>!#	8.55 (0.46) *<100>!#	356.91 (53.77) *<100>!#	177.77 (21.1) *<100>!#	68.38 (4.77) *<100>!#	53.02 (3.61) *<100>!#	58.16 (3.96) *<100>!#	1227.02 (279.43) *<100>!#	714.1 (104.4) *<100>!#	340.29 (34.83) *<100>!#	285.01 (25.67) *<100>!#	320.39 (31.92) *<100>!#
mde_ma1	1.22 (0.9) *<6>	1.02 (0.2) *<4>	0.99 (0.05) *<8>	1 (0.04) *<3>	1 (0.05) *<6>	6.49 (1.3) *<100>!#	3.6 (0.42) *<100>!#	1.82 (0.08) *<100>!#	1.59 (0.07) *<100>!#	1.63 (0.07) *<100>!#	36.66 (4.56) *<100>!#	18.72 (1.67) *<100>!#	7.84 (0.39) *<100>!#	6.41 (0.27) *<100>!#	6.87 (0.34) *<100>!#	214.38 (28.37) *<100>!#	109.29 (11.06) *<100>!#	46.05 (2.4) *<100>!#	37.74 (2.24) *<100>!#	44 (2.53) *<100>!#	803.59 (161.56) *<100>!#	471.9 (60.27) *<100>!#	237.22 (18.8) *<100>!#	205.18 (16.14) *<100>!#	233.11 (19.89) *<100>!#
mde_ma2	0.99 (0.73) *<2>	1.03 (0.19) *<2>	0.99 (0.05) *<7>	1 (0.04) *<5>	1 (0.04) *<3>	6.72 (1.08) *<100>!#	3.51 (0.38) *<100>!#	1.79 (0.09) *<100>!#	1.6 (0.06) *<100>!#	1.63 (0.07) *<100>!#	36.37 (4.11) *<100>!#	18.85 (1.76) *<100>!#	7.83 (0.43) *<100>!#	6.37 (0.28) *<100>!#	6.86 (0.34) *<100>!#	215.38 (30.02) *<100>!#	108.4 (10.89) *<100>!#	46.14 (3.07) *<100>!#	38.03 (2.41) *<100>!#	44.14 (2.98) *<100>!#	821.91 (138.41) *<100>!#	470.51 (60.57) *<100>!#	237.29 (20.85) *<100>!#	203.84 (15.39) *<100>!#	236.91 (19.39) *<100>!#
mde_mi3	1.08 (0.66) *<7>	1.04 (0.19) *<6>	1 (0.04) *<3>	1 (0.04) *<6>	1 (0.09) *<8>	7.32 (1.23) *<100>!#	3.91 (0.37) *<100>!#	1.98 (0.09) *<100>!#	1.83 (0.08) *<100>!#	1.95 (0.12) *<100>!#	46.21 (5.55) *<100>!#	24.5 (2.42) *<100>!#	10.46 (0.55) *<100>!#	8.92 (0.45) *<100>!#	10.34 (0.62) *<100>!#	310.23 (53.38) *<100>!#	152.85 (16.95) *<100>!#	68.28 (5.08) *<100>!#	57.03 (3.95) *<100>!#	69.79 (5.29) *<100>!#	1208.67 (238.94) *<100>!#	718.33 (117.79) *<100>!#	361.16 (41.88) *<100>!#	319.14 (31.8) *<100>!#	369.32 (42.47) *<100>!#
mde_mi3_1	1.12 (0.87) *<4>	1.05 (0.22) *<8>	0.99 (0.04) *<1>	1 (0.04) *<5>	1 (0.05) *<4>	6.53 (1.19) *<100>!#	3.54 (0.37) *<100>!#	1.79 (0.09) *<100>!#	1.62 (0.06) *<100>!#	1.63 (0.08) *<100>!#	35.81 (3.51) *<100>!#	18.9 (1.7) *<100>!#	7.81 (0.31) *<100>!#	6.39 (0.28) *<100>!#	6.92 (0.35) *<100>!#	216.74 (29.23) *<100>!#	108.49 (11.48) *<100>!#	46.38 (2.77) *<100>!#	37.78 (2.18) *<100>!#	44.25 (2.79) *<100>!#	817.48 (143.73) *<100>!#	471.79 (59.02) *<100>!#	235.21 (17.61) *<100>!#	204.69 (16.23) *<100>!#	235.07 (21.38) *<100>!#
mde_mi3_2	1.03 (0.88) *<4>	1.04 (0.23) *<9>	1 (0.05) *<4>	1 (0.04) *<3>	0.99 (0.04) *<2>	6.7 (1.18) *<100>!#	3.54 (0.4) *<100>!#	1.79 (0.08) *<100>!#	1.6 (0.07) *<100>!#	1.63 (0.07) *<100>!#	36 (4) *<100>!#	18.99 (1.75) *<100>!#	7.82 (0.4) *<100>!#	6.39 (0.29) *<100>!#	6.93 (0.31) *<100>!#	213.94 (30) *<100>!#	106.97 (9.72) *<100>!#	46.18 (3.19) *<100>!#	37.72 (2.19) *<100>!#	44.19 (3.12) *<100>!#	812.78 (157.6) *<100>!#	475.76 (65.14) *<100>!#	240.56 (24.77) *<100>!#	204.83 (15.24) *<100>!#	235.1 (23.39) *<100>!#
mde_mi3_bias	NA	NA	1.01 (0.08) *<3>	1 (0.05) *<5>	1.01 (0.04) *<2>	0.08 (0.03) *<100>!@	0.17 (0.03) *<100>!#	0.46 (0.03) *<100>!#	0.56 (0.03) *<100>!#	0.57 (0.03) *<100>!#	0.02 (0) *<100>!#	0.03 (0) *<100>!#	0.09 (0.01) *<100>!#	0.12 (0.01) *<100>!#	0.12 (0.01) *<100>!#	0 (0) *<100>!#	0.01 (0) *<100>!#	0.02 (0) *<100>!#	0.02 (0) *<100>!#	0.02 (0) *<100>!#	0 (0) *<100>!#	0 (0) *<100>!#	0 (0) *<100>!#	0 (0) *<100>!#	0 (0) *<100>!#
mde_mi4	7.13 (2.34) *<99>	3.46 (0.5) *<100>!#	1.45 (0.07) *<100>!#	1.09 (0.05) *<48>	1.03 (0.09) *<7>	12.96 (2.23) *<100>!#	6.55 (0.65) *<100>!#	2.63 (0.12) *<100>!#	2.08 (0.09) *<100>!#	2.09 (0.13) *<100>!#	59.24 (7.2) *<100>!#	30.49 (2.93) *<100>!#	12.49 (0.66) *<100>!#	10.21 (0.52) *<100>!#	11.58 (0.77) *<100>!#	353.55 (59.95) *<100>!#	181.29 (21.15) *<100>!#	78.78 (5.82) *<100>!#	64.96 (4.73) *<100>!#	77.64 (6.49) *<100>!#	1407.25 (347.7) *<100>!#	795.04 (128.48) *<100>!#	410.63 (47.95) *<100>!#	355.19 (34.29) *<100>!#	406.48 (42.88) *<100>!#
mde_mi4_1	5.22 (1.95) *<87>	2.8 (0.47) *<100>!@	1.28 (0.07) *<100>	1.04 (0.04) *<16>	1 (0.05) *<8>	9.8 (1.88) *<100>!#	5.05 (0.53) *<100>!#	2.12 (0.1) *<100>!#	1.71 (0.07) *<100>!#	1.67 (0.08) *<100>!#	41.33 (4.74) *<100>!#	21.15 (1.76) *<100>!#	8.56 (0.43) *<100>!#	6.74 (0.28) *<100>!#	7.22 (0.31) *<100>!#	232.35 (30.78) *<100>!#	115.94 (12.16) *<100>!#	48.95 (2.7) *<100>!#	39.9 (2.49) *<100>!#	45.83 (3.11) *<100>!#	877.34 (177.33) *<100>!#	487.32 (67.76) *<100>!#	253.48 (23.52) *<100>!#	212.85 (15.18) *<100>!#	242.26 (21.87) *<100>!#
mde_mi4_2	5.73 (2.21) *<91>	2.79 (0.48) *<100>	1.3 (0.07) *<100>	1.05 (0.04) *<24>	1.01 (0.05) *<8>	9.77 (1.64) *<100>!#	5.02 (0.54) *<100>!#	2.1 (0.1) *<100>!#	1.7 (0.07) *<100>!#	1.65 (0.08) *<100>!#	40.67 (4.75) *<100>!#	20.85 (1.78) *<100>!#	8.51 (0.47) *<100>!#	6.79 (0.28) *<100>!#	7.2 (0.33) *<100>!#	227.76 (34.45) *<100>!#	113.74 (10.86) *<100>!#	48.91 (3.24) *<100>!#	39.87 (2.55) *<100>!#	45.92 (3.03) *<100>!#	867.44 (149.21) *<100>!#	488.62 (62.08) *<100>!#	248.45 (23.73) *<100>!#	214.85 (16.3) *<100>!#	239.9 (20.83) *<100>!#
mde_mi4_bias	NA	0.53 (0.33) *<42>	0.83 (0.07) *<62>	0.97 (0.04) *<9>	1 (0.04) *<3>	0.07 (0.02) *<100>!#	0.14 (0.02) *<100>!#	0.42 (0.03) *<100>!#	0.54 (0.03) *<100>!#	0.57 (0.03) *<100>!#	0.02 (0) *<100>!#	0.03 (0) *<100>!#	0.09 (0.01) *<100>!#	0.12 (0.01) *<100>!#	0.12 (0.01) *<100>!#	0 (0) *<100>!#	0.01 (0) *<100>!#	0.02 (0) *<100>!#	0.02 (0) *<100>!#	0.02 (0) *<100>!#	0 (0) *<100>!#	0 (0) *<100>!#	0 (0) *<100>!#	0 (0) *<100>!#	0 (0) *<100>!#
mde_mi5	1.05 (0.55) *<3>	0.99 (0.17) *<5>	1 (0.04) *<3>	1.01 (0.04) *<1>	1 (0.08) *<5>	7.31 (1.15) *<100>!#	3.93 (0.37) *<100>!#	1.99 (0.1) *<100>!#	1.82 (0.08) *<100>!#	1.97 (0.13) *<100>!#	45.59 (6.45) *<100>!#	24.11 (2.25) *<100>!#	10.37 (0.66) *<100>!#	9 (0.51) *<100>!#	10.3 (0.62) *<100>!#	308.53 (55.1) *<100>!#	155.14 (20.47) *<100>!#	68.82 (5.27) *<100>!#	57.53 (4.06) *<100>!#	69.67 (5.51) *<100>!#	1228.55 (280.31) *<100>!#	700.2 (109.22) *<100>!#	365.22 (39.84) *<100>!#	314.29 (26.2) *<100>!#	369.73 (34.44) *<100>!#
mde_mi5_1	1.12 (0.71)	1.01 (0.22) *<4>	1 (0.05) *<2>	1 (0.03) *<1>	0.99 (0.05) *<7>	6.46 (1) *<100>!#	3.57 (0.4) *<100>!#	1.8 (0.09) *<100>!#	1.6 (0.06) *<100>!#	1.62 (0.08) *<100>!#	36.18 (4.75) *<100>!#	18.83 (1.62) *<100>!#	7.79 (0.42) *<100>!#	6.38 (0.31) *<100>!#	6.88 (0.31) *<100>!#	217.44 (30.31) *<100>!#	108.71 (10.09) *<100>!#	46.36 (3.2) *<100>!#	37.93 (2.13) *<100>!#	44.06 (2.86) *<100>!#	821.25 (151.94) *<100>!#	467.78 (61.69) *<100>!#	237.77 (22.25) *<100>!#	201.94 (13.62) *<100>!#	234 (21.12) *<100>!#
mde_mi5_2	0.99 (0.66)	0.97 (0.2) *<3>	1 (0.05) *<2>	1.01 (0.04) *<6>	1 (0.04) *<2>	6.78 (1.24) *<100>!#	3.52 (0.39) *<100>!#	1.79 (0.09) *<100>!#	1.6 (0.06) *<100>!#	1.63 (0.08) *<100>!#	35.67 (3.96) *<100>!#	18.56 (1.56) *<100>!#	7.78 (0.41) *<100>!#	6.44 (0.29) *<100>!#	6.9 (0.31) *<100>!#	215.38 (29.19) *<100>!#	108.16 (11.36) *<100>!#	46.2 (2.91) *<100>!#	37.7 (2.07) *<100>!#	44.02 (3.05) *<100>!#	853.14 (160.89) *<100>!#	474.85 (63.92) *<100>!#	240.99 (21.64) *<100>!#	202.95 (14.72) *<100>!#	234.15 (20.04) *<100>!#
mde_mi5_bias	NA	NA	1.01 (0.08) *<4>	1 (0.05) *<5>	1 (0.05) *<7>	0.08 (0.03) *<100>!#	0.18 (0.03) *<100>!#	0.46 (0.03) *<100>!#	0.56 (0.02) *<100>!#	0.57 (0.03) *<100>!#	0.02 (0) *<100>!#	0.03 (0) *<100>!#	0.09 (0.01) *<100>!#	0.12 (0.01) *<100>!#	0.12 (0.01) *<100>!#	0 (0) *<100>!#	0.01 (0) *<100>!#	0.02 (0) *<100>!#	0.02 (0) *<100>!#	0.02 (0) *<100>!#	0 (0) *<100>!#	0 (0) *<100>!#	0 (0) *<100>!#	0 (0) *<100>!#	0 (0) *<100>!#
mde_mi6	6.8 (2.23) *<100>	3.52 (0.5) *<100>!#	1.45 (0.07) *<100>!#	1.09 (0.05) *<44>	1.02 (0.08) *<8>	12.97 (2.11) *<100>!#	6.65 (0.63) *<100>!#	2.62 (0.13) *<100>!#	2.1 (0.1) *<100>!#	2.11 (0.14) *<100>!#	59 (7.57) *<100>!#	30.28 (2.92) *<100>!#	12.56 (0.63) *<100>!#	10.25 (0.52) *<100>!#	11.59 (0.74) *<100>!#	366.94 (68.82) *<100>!#	177.72 (19.69) *<100>!#	78.75 (6.38) *<100>!#	65.01 (3.58) *<100>!#	77.77 (5.89) *<100>!#	1452.46 (340.67) *<100>!#	814.37 (144.73) *<100>!#	409.94 (47.49) *<100>!#	358.84 (36.93) *<100>!#	409.86 (50.09) *<100>!#
mde_mi6_1	5.37 (1.73) *<93>	2.86 (0.49) *<100>!#	1.28 (0.07) *<100>	1.04 (0.04) *<24>	1.01 (0.04) *<5>	9.88 (1.58) *<100>!#	5.04 (0.52) *<100>!#	2.1 (0.1) *<100>!#	1.71 (0.07) *<100>!#	1.67 (0.07) *<100>!#	41.44 (4.44) *<100>!#	21.11 (1.89) *<100>!#	8.52 (0.43) *<100>!#	6.79 (0.27) *<100>!#	7.16 (0.31) *<100>!#	231.54 (38.32) *<100>!#	114.98 (10.83) *<100>!#	48.94 (3.42) *<100>!#	39.73 (1.87) *<100>!#	45.81 (3) *<100>!#	874.79 (165.67) *<100>!#	495.61 (61.47) *<100>!#	248.29 (20.38) *<100>!#	214.25 (16.62) *<100>!#	242.63 (22.87) *<100>!#
mde_mi6_2	5.11 (1.93) *<87>	2.81 (0.43) *<100>!#	1.29 (0.07) *<100>	1.04 (0.04) *<19>	1 (0.05) *<8>	9.93 (1.74) *<100>!#	5.12 (0.49) *<100>!#	2.13 (0.1) *<100>!#	1.7 (0.08) *<100>!#	1.68 (0.07) *<100>!#	40.2 (4.96) *<100>!#	20.67 (1.66) *<100>!#	8.49 (0.47) *<100>!#	6.78 (0.28) *<100>!#	7.19 (0.37) *<100>!#	233.68 (35.16) *<100>!#	112.88 (11.62) *<100>!#	49.06 (2.99) *<100>!#	39.62 (2.1) *<100>!#	45.51 (2.64) *<100>!#	857.94 (153.86) *<100>!#	496.1 (62.79) *<100>!#	249.54 (23.22) *<100>!#	214.48 (18.55) *<100>!#	241.93 (19.69) *<100>!#
mde_mi6_bias	NA	0.52 (0.38) *<44>	0.83 (0.06) *<70>	0.97 (0.04) *<13>	0.99 (0.04) *<3>	0.06 (0.02) *<100>!#	0.14 (0.02) *<100>!#	0.42 (0.03) *<100>!#	0.54 (0.03) *<100>!#	0.56 (0.02) *<100>!#	0.02 (0) *<100>!#	0.03 (0) *<100>!#	0.09 (0) *<100>!#	0.12 (0.01) *<100>!#	0.12 (0.01) *<100>!#	0 (0) *<100>!#	0.01 (0) *<100>!#	0.02 (0) *<100>!#	0.02 (0) *<100>!#	0.02 (0) *<100>!#	0 (0) *<100>!#	0 (0) *<100>!#	0 (0) *<100>!#	0 (0) *<100>!#	0 (0) *<100>!#
mde_mi7	1.09 (0.64) *<5>	0.97 (0.16) *<3>	1 (0.05) *<3>	1 (0.05) *<5>	0.99 (0.07) *<4>	7.36 (1.23) *<100>!#	3.92 (0.36) *<100>!#	1.99 (0.08) *<100>!#	1.83 (0.08) *<100>!#	1.95 (0.12) *<100>!#	46.24 (5.42) *<100>!#	24.3 (1.98) *<100>!#	10.45 (0.6) *<100>!#	8.89 (0.49) *<100>!#	10.34 (0.66) *<100>!#	303.81 (56.51) *<100>!#	156.56 (21.8) *<100>!#	67.89 (5.2) *<100>!#	57.19 (4.24) *<100>!#	70.65 (6.52) *<100>!#	1177.3 (234.6) *<100>!#	706.91 (118.23) *<100>!#	366.64 (39.23) *<100>!#	313.37 (31.75) *<100>!#	367.07 (35.47) *<100>!#
mde_mi7_1	1.07 (0.8) *<4>	0.96 (0.2) *<4>	1.01 (0.06) *<7>	1 (0.04) *<8>	0.99 (0.05) *<7>	6.66 (1.21) *<100>!#	3.61 (0.35) *<100>!#	1.8 (0.08) *<100>!#	1.6 (0.06) *<100>!#	1.62 (0.08) *<100>!#	36.71 (4.62) *<100>!#	18.74 (1.52) *<100>!#	7.83 (0.43) *<100>!#	6.36 (0.26) *<100>!#	6.9 (0.4) *<100>!#	215.71 (31.63) *<100>!#	108.24 (10.69) *<100>!#	46.13 (2.9) *<100>!#	37.97 (2.17) *<100>!#	44.03 (2.9) *<100>!#	814.9 (141.11) *<100>!#	476.79 (65.93) *<100>!#	239.92 (21.94) *<100>!#	204.04 (15.87) *<100>!#	232.54 (20.8) *<100>!#
mde_mi7_2	1.11 (0.86) *<2>	0.98 (0.21) *<6>	1 (0.05) *<3>	1 (0.04) *<4>	1 (0.05) *<4>	6.72 (1.39) *<100>!#	3.5 (0.34) *<100>!#	1.79 (0.08) *<100>!#	1.6 (0.06) *<100>!#	1.61 (0.07) *<100>!#	35.91 (4.73) *<100>!#	18.68 (1.57) *<100>!#	7.81 (0.41) *<100>!#	6.38 (0.3) *<100>!#	6.93 (0.3) *<100>!#	217.56 (34) *<100>!#	109.34 (11.1) *<100>!#	45.32 (2.49) *<100>!#	37.64 (1.87) *<100>!#	44.33 (2.93) *<100>!#	802.33 (136.95) *<100>!#	466.21 (61.16) *<100>!#	239.31 (21.59) *<100>!#	203.09 (17.71) *<100>!#	232.05 (18.88) *<100>!#
mde_mi7_bias	NA	NA	1 (0.08) *<5>	1 (0.05) *<4>	1 (0.05) *<4>	0.08 (0.02) *<100>!#	0.17 (0.03) *<100>!#	0.45 (0.03) *<100>!#	0.56 (0.02) *<100>!#	0.58 (0.03) *<100>!#	0.02 (0) *<100>!#	0.03 (0) *<100>!#	0.09 (0.01) *<100>!#	0.12 (0.01) *<100>!#	0.12 (0.01) *<100>!#	0 (0) *<100>!#	0.01 (0) *<100>!#	0.02 (0) *<100>!#	0.02 (0) *<100>!#	0.02 (0) *<100>!#	0 (0) *<100>!#	0 (0) *<100>!#	0 (0) *<100>!#	0 (0) *<100>!#	0 (0) *<100>!#
mde_mi8	6.93 (2.53) *<99>	3.44 (0.45) *<100>!#	1.45 (0.07) *<100>!#	1.1 (0.05) *<47>	1.02 (0.08) *<4>	13.06 (1.85) *<100>!#	6.6 (0.69) *<100>!#	2.62 (0.12) *<100>!#	2.07 (0.11) *<100>!#	2.08 (0.14) *<100>!#	58.39 (7.42) *<100>!#	30.74 (2.75) *<100>!#	12.44 (0.7) *<100>!#	10.3 (0.51) *<100>!#	11.37 (0.65) *<100>!#	357.1 (60.75) *<100>!#	180.76 (21.96) *<100>!#	78.1 (5.81) *<100>!#	65.56 (5.1) *<100>!#	78.35 (6.79) *<100>!#	1419.36 (375.48) *<100>!#	828.79 (148.71) *<100>!#	414.93 (44.94) *<100>!#	354.15 (37.48) *<100>!#	413.17 (46.23) *<100>!#
mde_mi8_1	5.16 (2.37) *<80>	2.78 (0.43) *<100>!@	1.3 (0.06) *<100>	1.05 (0.04) *<22>	1 (0.05) *<4>	9.72 (1.6) *<100>!#	5.07 (0.56) *<100>!#	2.12 (0.11) *<100>!#	1.7 (0.08) *<100>!#	1.66 (0.08) *<100>!#	40.32 (4.79) *<100>!#	21.05 (1.64) *<100>!#	8.55 (0.44) *<100>!#	6.79 (0.28) *<100>!#	7.11 (0.33) *<100>!#	228.92 (30.19) *<100>!#	113.75 (11.38) *<100>!#	48.42 (3.07) *<100>!#	39.9 (2.35) *<100>!#	46.11 (3.14) *<100>!#	858.96 (154.97) *<100>!#	493.19 (70.45) *<100>!#	249.44 (22.94) *<100>!#	212.92 (16.81) *<100>!#	240.65 (20.8) *<100>!#
mde_mi8_2	5.6 (2.32) *<88>	2.79 (0.39) *<100>!@	1.29 (0.07) *<100>	1.05 (0.04) *<19>	1 (0.05) *<5>	10.02 (1.42) *<100>!#	5.08 (0.5) *<100>!#	2.12 (0.1) *<100>!#	1.69 (0.07) *<100>!#	1.66 (0.08) *<100>!#	40.94 (4.57) *<100>!#	21.12 (1.72) *<100>!#	8.43 (0.43) *<100>!#	6.77 (0.28) *<100>!#	7.14 (0.34) *<100>!#	228.77 (28.49) *<100>!#	114.76 (10.67) *<100>!#	48.83 (3.14) *<100>!#	40.03 (2.39) *<100>!#	46.19 (3.22) *<100>!#	871.19 (154.01) *<100>!#	506.87 (74.04) *<100>!#	251.15 (20.73) *<100>!#	212.12 (18.95) *<100>!#	242.08 (19.94) *<100>!#
mde_mi8_bias	NA	0.52 (0.32) *<41>	0.81 (0.06) *<77>	0.97 (0.04) *<1>	1 (0.04) *<3>	0.06 (0.02) *<100>!#	0.14 (0.02) *<100>!#	0.42 (0.03) *<100>!#	0.54 (0.03) *<100>!#	0.57 (0.03) *<100>!#	0.02 (0) *<100>!#	0.03 (0) *<100>!#	0.09 (0.01) *<100>!#	0.12 (0.01) *<100>!#	0.12 (0.01) *<100>!#	0 (0) *<100>!#	0.01 (0) *<100>!#	0.02 (0) *<100>!#	0.02 (0) *<100>!#	0.02 (0) *<100>!#	0 (0) *<100>!#	0 (0) *<100>!#	0 (0) *<100>!#	0 (0) *<100>!#	0 (0) *<100>!#
mde_mi9	1.05 (0.74) *<2>	1.03 (0.22) *<3>	1 (0.06) *<8>	1.01 (0.04) *<7>	1 (0.05) *<5>	6.68 (1.21) *<100>!#	3.55 (0.38) *<100>!#	1.79 (0.09) *<100>!#	1.6 (0.06) *<100>!#	1.63 (0.08) *<100>!#	36.05 (4) *<100>!#	18.78 (1.66) *<100>!#	7.8 (0.44) *<100>!#	6.39 (0.26) *<100>!#	6.85 (0.34) *<100>!#	214.7 (31.07) *<100>!#	109.04 (10.27) *<100>!#	46.1 (2.42) *<100>!#	37.81 (2.14) *<100>!#	43.85 (2.75) *<100>!#	823.05 (147.81) *<100>!#	475.75 (63.16) *<100>!#	236.22 (20.26) *<100>!#	203.48 (15.85) *<100>!#	230.13 (19.47) *<100>!#
mde_bias1	NA	NA	NA	NA	NA	NA	NA	NA	NA	NA	NA	NA	NA	NA	NA	NA	NA	NA	NA	NA	NA	NA	NA	NA	NA
mde_bias2	NA	NA	NA	NA	NA	NA	NA	NA	NA	NA	NA	NA	NA	NA	NA	NA	NA	NA	NA	NA	NA	NA	NA	NA	NA
mde_bias	NA	NA	NA	NA	NA	NA	NA	NA	NA	NA	NA	NA	NA	NA	NA	NA	NA	NA	NA	NA	NA	NA	NA	NA	NA

**Table 13:** Odds ratios between dysthymic disorder and its input symptoms by assumed symptom prevalence and correlations NA = not applicable due to 0 case in the denominators for the calculation of odds ratios, except for dys_mi_bias that is a continuous variable Mean (standard deviation) *<number> = the numbers of significant statistics (p < 0.05) in 100 simulations !# = p < 0.001 in all of the 100 simulations !@ = p < 0.001 in some of the 100 simulations Note: See Table 1 for variable definitions and Table 2 for the prevalence of major depressive episodes, dysthymic disorder, and manic episodes. dys = Dysthymic Disorder; dys_ma = Depressed mood most of the day for more days than not, for at least 2 years; dys_mi = Minor criteria (at least 2 items); dys_mi1 = Poor appetite or overeating; dys_mi1_1 = Poor appetite; dys_mi1_2 = Overeating; dys_mi1_bias = Information of the domain not explained by the input variables; dys_mi4 = Low self-esteem; dys_mi6 = Feelings of hopelessness; dys_mi_bias = Information of minor criteria not explained by input variables; dys_bias = Information of diagnosis not explained by major or minor criteria

Assumed symptom prevalence	0.05	0.1	0.3	0.5	0.7	0.05	0.1	0.3	0.5	0.7	0.05	0.1	0.3	0.5	0.7	0.05	0.1	0.3	0.5	0.7	0.05	0.1	0.3	0.5	0.7
Assumed symptom correlations	0	0	0	0	0	0.1	0.1	0.1	0.1	0.1	0.4	0.4	0.4	0.4	0.4	0.7	0.7	0.7	0.7	0.7	0.9	0.9	0.9	0.9	0.9
dys	NA	NA	NA	NA	NA	NA	NA	NA	NA	NA	NA	NA	NA	NA	NA	NA	NA	NA	NA	NA	NA	NA	NA	NA	NA
dys_ma	NA	NA	NA	NA	NA	NA	NA	NA	NA	NA	NA	NA	NA	NA	NA	NA	NA	NA	NA	NA	NA	NA	NA	NA	NA
dys_mi	NA	NA	NA	NA	NA	NA	NA	NA	NA	NA	NA	NA	NA	NA	NA	NA	NA	NA	NA	NA	NA	NA	NA	NA	NA
dys_mi1	7.05 (2.65) *<99>	3.51 (0.45) *<100>!#	1.46 (0.07) *<100>!#	1.08 (0.04) *<42>	1.02 (0.07) *<3>	13.06 (2.14) *<100>!#	6.51 (0.63) *<100>!#	2.63 (0.13) *<100>!#	2.09 (0.1) *<100>!#	2.11 (0.14) *<100>!#	59.61 (7.72) *<100>!#	30.61 (2.61) *<100>!#	12.4 (0.57) *<100>!#	10.21 (0.54) *<100>!#	11.49 (0.77) *<100>!#	366.08 (64.5) *<100>!#	181.58 (25.12) *<100>!#	78.32 (6.97) *<100>!#	65.67 (4.32) *<100>!#	77.99 (6.32) *<100>!#	1379.95 (296.97) *<100>!#	817.89 (141.51) *<100>!#	422.53 (47.69) *<100>!#	353.87 (35.93) *<100>!#	415.1 (47.89) *<100>!#
dys_mi1_1	5.43 (2.13) *<90>	2.84 (0.35) *<100>!#	1.28 (0.07) *<100>	1.04 (0.04) *<12>	1.01 (0.05) *<7>	9.92 (1.51) *<100>!#	5.04 (0.54) *<100>!#	2.12 (0.1) *<100>!#	1.7 (0.06) *<100>!#	1.67 (0.08) *<100>!#	40.47 (5.01) *<100>!#	21.53 (1.93) *<100>!#	8.5 (0.41) *<100>!#	6.75 (0.32) *<100>!#	7.21 (0.38) *<100>!#	232.55 (34.68) *<100>!#	114.38 (13.1) *<100>!#	48.57 (3.31) *<100>!#	40.19 (2.33) *<100>!#	45.73 (3.19) *<100>!#	866.1 (193.89) *<100>!#	493.76 (61.01) *<100>!#	250.36 (22.06) *<100>!#	209.25 (16.45) *<100>!#	244.46 (20.04) *<100>!#
dys_mi1_2	5.37 (2.36) *<89>	2.8 (0.39) *<100>!@	1.3 (0.07) *<100>!@	1.05 (0.04) *<15>	1 (0.05) *<5>	9.88 (1.67) *<100>!#	5 (0.5) *<100>!#	2.12 (0.12) *<100>!#	1.7 (0.06) *<100>!#	1.67 (0.08) *<100>!#	40.89 (5.14) *<100>!#	20.63 (1.65) *<100>!#	8.45 (0.33) *<100>!#	6.81 (0.28) *<100>!#	7.12 (0.3) *<100>!#	228.96 (27.72) *<100>!#	114.41 (11.26) *<100>!#	48.84 (3.01) *<100>!#	39.8 (2.12) *<100>!#	45.87 (3.13) *<100>!#	855.27 (151.35) *<100>!#	506.77 (74.42) *<100>!#	252.25 (23.53) *<100>!#	213.41 (17.98) *<100>!#	241.66 (21.33) *<100>!#
dys_mi1_bias	NA	0.59 (0.51) *<40>	0.83 (0.07) *<63>	0.97 (0.04) *<7>	1 (0.05) *<7>	0.06 (0.02) *<100>!#	0.14 (0.03) *<100>!#	0.42 (0.03) *<100>!#	0.55 (0.02) *<100>!#	0.57 (0.03) *<100>!#	0.02 (0) *<100>!#	0.03 (0) *<100>!#	0.09 (0.01) *<100>!#	0.12 (0.01) *<100>!#	0.12 (0.01) *<100>!#	0 (0) *<100>!#	0.01 (0) *<100>!#	0.02 (0) *<100>!#	0.02 (0) *<100>!#	0.02 (0) *<100>!#	0 (0) *<100>!#	0 (0) *<100>!#	0 (0) *<100>!#	0 (0) *<100>!#	0 (0) *<100>!#
dys_mi4	5.93 (2.42) *<91>	3.24 (0.44) *<100>!#	1.34 (0.07) *<100>!@	1.05 (0.04) *<23>	1 (0.05) *<7>	9.99 (1.74) *<100>!#	5.23 (0.56) *<100>!#	2.19 (0.11) *<100>!#	1.71 (0.07) *<100>!#	1.67 (0.08) *<100>!#	41.43 (4.99) *<100>!#	21.35 (2.08) *<100>!#	8.6 (0.43) *<100>!#	6.88 (0.29) *<100>!#	7.2 (0.33) *<100>!#	235.83 (33.75) *<100>!#	118.42 (13.03) *<100>!#	49.28 (3.23) *<100>!#	40.28 (2.23) *<100>!#	46.56 (3.12) *<100>!#	870.15 (158.66) *<100>!#	506.74 (69.64) *<100>!#	250.83 (21.59) *<100>!#	215.1 (17.55) *<100>!#	245.77 (23) *<100>!#
dys_mi6	6.17 (2.11) *<95>	3.12 (0.4) *<100>!#	1.35 (0.07) *<100>!@	1.05 (0.04) *<24>	1 (0.05) *<2>	10.41 (2.09) *<100>!#	5.29 (0.57) *<100>!#	2.18 (0.11) *<100>!#	1.73 (0.08) *<100>!#	1.66 (0.08) *<100>!#	40.8 (4.49) *<100>!#	21.41 (1.85) *<100>!#	8.6 (0.39) *<100>!#	6.84 (0.33) *<100>!#	7.23 (0.31) *<100>!#	230.87 (32.06) *<100>!#	116.69 (11.9) *<100>!#	49.37 (3.03) *<100>!#	40.1 (2.37) *<100>!#	46.2 (3.08) *<100>!#	884.32 (158.68) *<100>!#	499.7 (70.28) *<100>!#	253.34 (23.19) *<100>!#	214.08 (16.7) *<100>!#	243.27 (21.72) *<100>!#
dys_mi_bias	NA	NA	NA	NA	NA	NA	NA	NA	NA	NA	NA	NA	NA	NA	NA	NA	NA	NA	NA	NA	NA	NA	NA	NA	NA
dys_bias	0 (0) *<100>!#	0 (0) *<100>!#	0 (0) *<100>!#	0 (0) *<100>!#	0 (0)	0 (0) *<100>!#	0 (0) *<100>!#	0 (0) *<100>!#	0 (0) *<100>!#	0 (0) *<100>!#	0 (0) *<100>!#	0 (0) *<100>!#	0 (0) *<100>!#	0 (0) *<100>!#	0 (0) *<100>!#	0 (0) *<100>!#	0 (0) *<100>!#	0 (0) *<100>!#	0 (0) *<100>!#	0 (0) *<100>!#	0 (0) *<100>!#	0 (0) *<100>!#	0 (0) *<100>!#	0 (0) *<100>!#	0 (0) *<100>!#

**Table 14:** Odds ratios between dysthymic disorder and the input symptoms of manic episodes by assumed symptom prevalence and correlations NA = not applicable due to 0 case in the denominators for the calculation of odds ratios Mean (standard deviation) *<number> = the numbers of significant statistics (p < 0.05) in 100 simulations !# = p < 0.001 in all of the 100 simulations !@ = p < 0.001 in some of the 100 simulations manic = Manic episodes; man_ma1 = Elevated mood; man_ma2 = Expansive mood; man_ma3 = Irritable mood; man_mi1 = Increased self-esteem or grandiosity; man_mi1_1 = Increased self-esteem; man_mi1_2 = Grandiosity; man_mi1_bias = Information of the domain not explained by the input variables; man_mi2 = Decreased need for sleep (e.g., feels rested after only 3 hours of sleep); man_mi3 = More talkative than usual or pressure to keep talking; man_mi3_1 = More talkative than usual; man_mi3_2 = Pressure to keep talking; man_mi3_bias = Information of the domain not explained by the input variables; man_mi4 = Flight of ideas or subjective experience that thoughts are racing; man_mi4_1 = Flight of ideas; man_mi4_2 = Subjective experience that thoughts are racing; man_mi4_bias = Information of the domain not explained by the input variables; man_mi5 = Distractibility (i.e., attention too easily drawn to unimportant or irrelevant external stimuli); man_mi6 = Increase in goal-directed activity (either socially, at work or school, or sexually) or psychomotor agitation; man_mi6_1 = Increase in goal-directed activity ; man_mi6_2 = Psychomotor agitation; man_mi6_bias = Information of the domain not explained by the input variables; man_mi7 = Excessive involvement in pleasurable activities that have a high potential for painful consequences (e.g., engaging in unrestrained buying sprees, sexual indiscretions, or foolish business investments); man_bias1 = Information of diagnosis due to top-censoring for choosing at least three symptoms; man_bias2 = Information of diagnosis due to top-censoring for choosing at least four symptoms; man_bias = Information of diagnosis not explained by symptoms

Assumed symptom prevalence	0.05	0.1	0.3	0.5	0.7	0.05	0.1	0.3	0.5	0.7	0.05	0.1	0.3	0.5	0.7	0.05	0.1	0.3	0.5	0.7	0.05	0.1	0.3	0.5	0.7
Assumed symptom correlations	0	0	0	0	0	0.1	0.1	0.1	0.1	0.1	0.4	0.4	0.4	0.4	0.4	0.7	0.7	0.7	0.7	0.7	0.9	0.9	0.9	0.9	0.9
manic	0 (0)	1.08 (1.35) *<3>	1 (0.06) *<2>	1 (0.04) *<2>	0.99 (0.05) *<6>	0.94 (0.72)	0.99 (0.3) *<3>	1.01 (0.06) *<4>	1.01 (0.04) *<7>	1 (0.05) *<5>	1 (0.33) *<4>	1.03 (0.16) *<4>	1.01 (0.06) *<12>	1 (0.04) *<5>	1.01 (0.05) *<6>	1.01 (0.24) *<2>	0.99 (0.13) *<6>	1 (0.05) *<6>	1 (0.04) *<9>	0.99 (0.05) *<6>	0.98 (0.22) *<6>	1 (0.11) *<4>	1 (0.05) *<3>	1 (0.04) *<3>	1.01 (0.04) *<3>
man_ma1	0.97 (0.67) *<1>	1 (0.24) *<6>	1 (0.05) *<5>	1 (0.04) *<6>	1 (0.05) *<4>	1.06 (0.36) *<4>	1 (0.17) *<6>	1 (0.06) *<10>	1 (0.04) *<5>	1 (0.05) *<4>	1.01 (0.28) *<8>	1.02 (0.13) *<2>	1.01 (0.06) *<8>	1 (0.04) *<2>	1 (0.04) *<3>	1 (0.2) *<3>	1 (0.12) *<4>	1 (0.05) *<6>	1 (0.04) *<7>	1 (0.05) *<5>	0.99 (0.22) *<4>	1 (0.11) *<5>	1 (0.05) *<1>	0.99 (0.03) *<2>	1.01 (0.05) *<3>
man_ma2	1.12 (0.74) *<3>	1.03 (0.24) *<9>	1 (0.05) *<3>	1 (0.04) *<6>	1 (0.05) *<8>	0.95 (0.33) *<5>	1.01 (0.16) *<5>	1 (0.05) *<4>	1.01 (0.04) *<10>	1 (0.05) *<4>	1 (0.25) *<7>	1 (0.11) *<3>	1.01 (0.05) *<9>	1 (0.04) *<6>	0.99 (0.05) *<5>	1 (0.2) *<3>	1 (0.12) *<5>	1 (0.05) *<6>	1 (0.04) *<6>	1 (0.05) *<4>	0.99 (0.22) *<4>	1 (0.11) *<7>	1 (0.05) *<4>	0.99 (0.04) *<3>	1 (0.05) *<2>
man_ma3	0.93 (0.77) *<2>	1.02 (0.19) *<1>	1.01 (0.05) *<4>	0.99 (0.04) *<4>	1 (0.05) *<8>	1 (0.32) *<3>	0.97 (0.15) *<3>	1.01 (0.05) *<5>	1.01 (0.04) *<5>	1 (0.05) *<3>	0.99 (0.23) *<4>	1 (0.13) *<8>	1.01 (0.05) *<10>	1 (0.04) *<2>	1.01 (0.05) *<6>	1 (0.22) *<4>	1 (0.12) *<8>	1 (0.05) *<5>	1 (0.04) *<9>	0.99 (0.04) *<2>	0.99 (0.21) *<6>	1 (0.11) *<4>	1 (0.05) *<4>	1 (0.04) *<4>	1.01 (0.05) *<4>
man_mi1	1.09 (0.56) *<3>	1.01 (0.17) *<5>	0.99 (0.05) *<4>	1 (0.05) *<3>	1.01 (0.08) *<4>	1.01 (0.26) *<7>	1 (0.12) *<4>	1 (0.05) *<7>	1 (0.04) *<2>	1 (0.06) *<6>	1.04 (0.2) *<4>	1.02 (0.1) *<5>	1.01 (0.05) *<9>	1 (0.04) *<1>	1 (0.05) *<1>	1 (0.18) *<1>	0.99 (0.11) *<7>	1 (0.05) *<5>	1 (0.04) *<5>	1 (0.06) *<8>	0.99 (0.2) *<5>	1 (0.1) *<5>	1 (0.05) *<2>	0.99 (0.04) *<1>	1.01 (0.05) *<3>
man_mi1_1	1.07 (0.74) *<1>	1.03 (0.21) *<4>	1 (0.05) *<3>	1 (0.04) *<4>	1 (0.05) *<7>	0.97 (0.32) *<4>	1.01 (0.16) *<4>	1 (0.05) *<6>	1.01 (0.04) *<6>	1 (0.05) *<5>	1.06 (0.24) *<3>	1.01 (0.13) *<4>	1.01 (0.05) *<10>	1 (0.04) *<6>	1 (0.05) *<5>	0.99 (0.19) *<1>	0.98 (0.12) *<9>	1 (0.05) *<3>	1 (0.04) *<9>	1 (0.05) *<12>	0.99 (0.21) *<4>	1.01 (0.11) *<7>	1 (0.05) *<2>	0.99 (0.03) *<4>	1 (0.05) *<2>
man_mi1_2	1.09 (0.69) *<2>	0.99 (0.21) *<4>	0.99 (0.05) *<5>	1 (0.04) *<2>	1 (0.04) *<3>	1.02 (0.35) *<7>	0.99 (0.16) *<3>	1 (0.05) *<6>	1 (0.04) *<5>	0.99 (0.05) *<6>	1.01 (0.26) *<7>	1.02 (0.13) *<5>	1.01 (0.05) *<4>	1 (0.04) *<2>	1 (0.04) *<2>	1.01 (0.22) *<7>	1 (0.12) *<8>	1 (0.05) *<3>	1 (0.04) *<7>	1 (0.05) *<9>	0.97 (0.23) *<4>	1 (0.11) *<4>	1 (0.05) *<2>	0.99 (0.03) *<2>	1.01 (0.04) *<3>
man_mi1_bias	NA	NA	1.01 (0.08) *<5>	1 (0.04) *<5>	1 (0.04) *<3>	NA	1.17 (0.59) *<5>	1 (0.07) *<4>	1 (0.05) *<8>	1.01 (0.05) *<4>	1.14 (0.53) *<4>	1.04 (0.19) *<7>	1 (0.06) *<6>	1 (0.04) *<4>	1 (0.04) *<3>	1.07 (0.32) *<7>	1.02 (0.15) *<7>	1 (0.05) *<4>	1 (0.04) *<9>	1 (0.05) *<7>	1.09 (0.29) *<3>	1 (0.12) *<3>	1 (0.05) *<3>	1.01 (0.03) *<1>	0.99 (0.04) *<3>
man_mi2	1.03 (0.73) *<2>	1.01 (0.24) *<7>	1 (0.04) *<4>	1 (0.04) *<6>	1 (0.05) *<3>	1 (0.39) *<6>	1.02 (0.17) *<7>	1 (0.05) *<6>	1.01 (0.04) *<9>	1 (0.04) *<1>	1.01 (0.26) *<4>	1.02 (0.13) *<4>	1.01 (0.05) *<7>	1 (0.03) *<1>	1.01 (0.05) *<7>	1.02 (0.21) *<5>	0.99 (0.12) *<6>	1 (0.05) *<5>	1 (0.04) *<4>	1 (0.05) *<6>	0.98 (0.23) *<5>	1 (0.11) *<7>	1 (0.05) *<3>	1 (0.04) *<3>	1.01 (0.05) *<2>
man_mi3	0.85 (0.47) *<3>	1 (0.17) *<3>	1 (0.04) *<5>	1 (0.05) *<6>	1.01 (0.07) *<4>	1.02 (0.21) *<2>	0.99 (0.12) *<5>	0.98 (0.05) *<5>	1 (0.04) *<5>	1 (0.07) *<3>	1.02 (0.2) *<3>	1 (0.1) *<6>	1 (0.05) *<13>	1.01 (0.05) *<9>	1 (0.06) *<6>	0.99 (0.19) *<4>	0.99 (0.11) *<7>	1 (0.04) *<7>	1 (0.04) *<2>	1 (0.05) *<9>	0.98 (0.2) *<7>	1 (0.1) *<3>	1 (0.04) *<2>	0.99 (0.03) *<2>	1 (0.05) *<2>
man_mi3_1	0.91 (0.75) *<1>	1.04 (0.22) *<6>	1 (0.06) *<8>	1 (0.04) *<6>	1.01 (0.05) *<5>	1.03 (0.27) *<1>	0.99 (0.16) *<5>	0.99 (0.05) *<8>	1 (0.04) *<4>	0.99 (0.05) *<5>	1.03 (0.26) *<5>	1.01 (0.12) *<4>	1.01 (0.05) *<4>	1.01 (0.04) *<8>	1 (0.05) *<5>	0.99 (0.2) *<1>	0.99 (0.12) *<7>	1 (0.05) *<5>	1 (0.04) *<8>	0.99 (0.05) *<7>	0.98 (0.22) *<8>	1 (0.11) *<4>	1 (0.05) *<3>	0.99 (0.04) *<5>	1.01 (0.05) *<1>
man_mi3_2	0.82 (0.72) *<1>	0.98 (0.22) *<5>	1 (0.05) *<2>	1 (0.04) *<8>	1 (0.05) *<4>	1 (0.31) *<1>	0.99 (0.15) *<5>	0.99 (0.05) *<12>	1 (0.04) *<5>	1 (0.05) *<5>	1.01 (0.25) *<2>	1 (0.13) *<4>	1.01 (0.06) *<11>	1 (0.04) *<5>	1 (0.05) *<8>	1 (0.22) *<5>	1 (0.13) *<8>	1 (0.05) *<5>	1 (0.04) *<5>	1 (0.04) *<3>	0.97 (0.22) *<5>	1.01 (0.11) *<4>	1 (0.05) *<1>	0.99 (0.03) *<4>	1 (0.05) *<4>
man_mi3_bias	NA	NA	1.01 (0.08) *<6>	1 (0.05) *<10>	1 (0.04) *<7>	NA	1.17 (0.46) *<7>	1.02 (0.08) *<8>	1 (0.05) *<4>	1.01 (0.05) *<7>	1.27 (0.84) *<8>	1.02 (0.19) *<3>	0.99 (0.06) *<6>	1 (0.04) *<4>	1 (0.04) *<5>	1.06 (0.29) *<4>	1.01 (0.15) *<8>	1 (0.05) *<5>	1 (0.04) *<6>	1.01 (0.04) *<7>	1.09 (0.28) *<5>	1 (0.13) *<4>	1 (0.05) *<4>	1.01 (0.04) *<5>	1 (0.05) *<4>
man_mi4	0.97 (0.5) *<1>	0.99 (0.16) *<6>	1 (0.04) *<5>	1 (0.05) *<6>	1 (0.08) *<3>	0.96 (0.23) *<4>	0.99 (0.12) *<6>	0.99 (0.04) *<6>	1.01 (0.05) *<7>	0.99 (0.07) *<7>	1 (0.18) *<3>	1.01 (0.11) *<5>	1.01 (0.05) *<3>	1 (0.04) *<8>	1.01 (0.05) *<4>	1 (0.2) *<7>	1 (0.1) *<4>	1 (0.05) *<4>	1 (0.04) *<6>	1 (0.05) *<7>	0.99 (0.2) *<3>	1 (0.1) *<4>	1 (0.04) *<2>	0.99 (0.04) *<3>	1 (0.05) *<6>
man_mi4_1	0.98 (0.64) *<1>	1 (0.19) *<1>	0.99 (0.04) *<3>	1 (0.04) *<8>	1 (0.05) *<9>	0.96 (0.3) *<4>	0.98 (0.16) *<7>	1 (0.05) *<2>	1 (0.04) *<2>	1 (0.05) *<1>	0.99 (0.25) *<5>	1.01 (0.13) *<6>	1 (0.05) *<7>	1 (0.04) *<4>	1 (0.04) *<2>	1.01 (0.22) *<5>	1 (0.11) *<4>	1 (0.05) *<5>	1 (0.04) *<6>	1 (0.05) *<2>	0.98 (0.21) *<5>	1 (0.11) *<6>	1 (0.05) *<3>	0.99 (0.03) *<2>	1 (0.05) *<4>
man_mi4_2	0.99 (0.74) *<1>	0.97 (0.19) *<4>	1 (0.05) *<5>	1 (0.04) *<5>	1 (0.05) *<4>	0.97 (0.34) *<3>	1.01 (0.17) *<8>	0.99 (0.05) *<2>	1.01 (0.04) *<6>	1 (0.05) *<6>	1.01 (0.23) *<5>	1 (0.13) *<6>	1.01 (0.05) *<4>	1 (0.04) *<6>	1.01 (0.05) *<6>	0.99 (0.23) *<7>	1 (0.11) *<3>	1 (0.05) *<3>	1 (0.04) *<4>	1 (0.05) *<7>	0.99 (0.23) *<7>	1.01 (0.11) *<5>	1 (0.05) *<2>	0.99 (0.04) *<4>	1.01 (0.05) *<2>
man_mi4_bias	NA	NA	1.01 (0.08) *<3>	1 (0.05) *<5>	1 (0.04) *<5>	NA	1.2 (0.55) *<7>	1.01 (0.08) *<4>	1 (0.04) *<5>	1 (0.05) *<6>	1.19 (0.59) *<7>	1.02 (0.22) *<8>	1 (0.06) *<5>	1 (0.04) *<3>	1 (0.04) *<6>	1.09 (0.33) *<5>	1.01 (0.15) *<2>	1.01 (0.05) *<2>	1 (0.04) *<7>	1 (0.05) *<5>	1.09 (0.3) *<9>	1.01 (0.13) *<7>	1 (0.05) *<2>	1.01 (0.03) *<3>	1 (0.05) *<3>
man_mi5	1.03 (0.76) *<3>	0.95 (0.2) *<3>	1.01 (0.06) *<6>	1 (0.04) *<2>	1.01 (0.05) *<7>	1 (0.37) *<6>	0.99 (0.14) *<3>	0.99 (0.04) *<2>	1.01 (0.04) *<7>	1 (0.04) *<3>	1 (0.24) *<4>	1 (0.12) *<2>	1.01 (0.05) *<6>	1 (0.04) *<5>	1 (0.05) *<3>	0.98 (0.21) *<5>	1 (0.12) *<4>	1 (0.05) *<5>	1 (0.04) *<9>	0.99 (0.05) *<8>	0.97 (0.24) *<7>	1 (0.11) *<5>	1 (0.05) *<4>	0.99 (0.04) *<3>	1 (0.05) *<5>
man_mi6	1.06 (0.57) *<3>	0.99 (0.16) *<7>	1 (0.05) *<8>	1 (0.05) *<6>	1 (0.08) *<7>	0.99 (0.27) *<6>	1.01 (0.11) *<4>	1 (0.05) *<8>	1.01 (0.05) *<4>	1 (0.07) *<6>	1 (0.18) *<4>	1.01 (0.11) *<6>	1.01 (0.05) *<10>	1 (0.04) *<8>	1 (0.06) *<4>	1.02 (0.19) *<2>	1 (0.11) *<8>	1 (0.04) *<4>	1 (0.04) *<2>	1 (0.05) *<4>	0.99 (0.21) *<6>	1 (0.11) *<5>	1 (0.05) *<4>	0.99 (0.03) *<2>	1.01 (0.05) *<3>
man_mi6_1	1.1 (0.76) *<2>	1.01 (0.23) *<6>	1 (0.05) *<7>	0.99 (0.04) *<6>	1 (0.04) *<2>	0.98 (0.36) *<6>	0.99 (0.15) *<6>	1 (0.05) *<9>	1.01 (0.04) *<8>	1 (0.05) *<5>	0.96 (0.23) *<2>	1.01 (0.13) *<6>	1.01 (0.05) *<7>	1 (0.04) *<7>	1 (0.05) *<7>	1.02 (0.23) *<6>	1 (0.12) *<4>	1 (0.05) *<4>	1 (0.04) *<6>	1 (0.05) *<7>	0.99 (0.23) *<7>	1 (0.11) *<7>	1 (0.05) *<2>	0.99 (0.03) *<4>	1.01 (0.05) *<4>
man_mi6_2	1.02 (0.79) *<4>	0.98 (0.22) *<6>	1 (0.06) *<8>	1 (0.04) *<4>	1 (0.05) *<3>	1 (0.36) *<3>	1.03 (0.14) *<1>	1 (0.05) *<7>	1 (0.04) *<5>	1 (0.05) *<7>	1.02 (0.26) *<6>	1.01 (0.14) *<6>	1.01 (0.06) *<12>	0.99 (0.04) *<2>	1 (0.05) *<6>	1.03 (0.22) *<4>	0.98 (0.11) *<5>	1 (0.05) *<4>	1 (0.04) *<6>	1 (0.05) *<4>	0.98 (0.23) *<8>	1 (0.11) *<4>	1 (0.05) *<4>	0.99 (0.03) *<2>	1.01 (0.05) *<3>
man_mi6_bias	NA	NA	1 (0.08) *<5>	1 (0.05) *<5>	1 (0.05) *<7>	NA	1.13 (0.47) *<6>	1.01 (0.07) *<7>	1 (0.04) *<1>	1 (0.04) *<5>	1.3 (0.76) *<9>	1.04 (0.2) *<8>	1 (0.06) *<8>	1.01 (0.04) *<4>	1 (0.04) *<6>	1.06 (0.34) *<6>	1.03 (0.14) *<3>	1 (0.05) *<5>	1 (0.04) *<7>	1 (0.05) *<6>	1.12 (0.32) *<7>	1.02 (0.13) *<6>	1 (0.05) *<4>	1.01 (0.04) *<5>	1 (0.04) *<5>
man_mi7	1 (0.81) *<5>	0.97 (0.18) *<3>	1.01 (0.04) *<3>	1 (0.04) *<4>	1 (0.05) *<6>	1.03 (0.3) *<1>	1.01 (0.16) *<3>	1 (0.05) *<2>	1 (0.04) *<2>	1 (0.05) *<3>	1.01 (0.24) *<4>	1.02 (0.12) *<5>	1.01 (0.05) *<5>	1 (0.04) *<7>	1 (0.05) *<5>	1.01 (0.22) *<4>	0.99 (0.11) *<3>	1 (0.05) *<3>	1 (0.04) *<7>	1 (0.05) *<9>	0.98 (0.23) *<9>	1 (0.11) *<4>	1 (0.05) *<4>	0.99 (0.04) *<4>	1 (0.05) *<3>
man_bias1	NA	NA	NA	NA	NA	NA	NA	NA	NA	NA	NA	NA	NA	NA	NA	NA	NA	NA	NA	NA	NA	NA	NA	NA	NA
man_bias2	NA	NA	NA	NA	NA	NA	NA	NA	NA	NA	NA	NA	NA	NA	NA	NA	NA	NA	NA	NA	NA	NA	NA	NA	NA
man_bias	NA	NA	NA	NA	NA	NA	NA	NA	NA	NA	NA	NA	NA	NA	NA	NA	NA	NA	NA	NA	NA	NA	NA	NA	NA

**Table 15:** Correlations between manic episodes and the input symptoms of major depressive episodes by assumed symptom prevalence and correlations Mean (standard deviation) *<number> = the numbers of significant statistics (p < 0.05) in 100 simulations !# = p < 0.001 in all of the 100 simulations !@ = p < 0.001 in some of the 100 simulations mde = Major Depressive Episodes for the diagnosis of Major Depressive Disorder; mde_ma1 = Depressed mood for more than two weeks.; mde_ma2 = Loss of interest or pleasure in daily activities for more than two weeks.; mde_mi3 = Significant unintentional weight loss or gain; mde_mi3_1 = Significant unintentional weight gain; mde_mi3_2 = Significant unintentional weight loss; mde_mi3_bias = Information of the domain not explained by the input variables; mde_mi4 = Insomnia or sleeping too much; mde_mi4_1 = Insomnia; mde_mi4_2 = Sleeping too much; mde_mi4_bias = Information of the domain not explained by the input variables; mde_mi5 = Agitation or psychomotor retardation noticed by others; mde_mi5_1 = Agitation; mde_mi5_2 = Psychomotor retardation noticed by others; mde_mi5_bias = Information of the domain not explained by the input variables; mde_mi6 = Fatigue or loss of energy; mde_mi6_1 = Fatigue; mde_mi6_2 = Loss of energy; mde_mi6_bias = Information of the domain not explained by the input variables; mde_mi7 = Feelings of worthlessness or excessive guilt; mde_mi7_1 = Feelings of worthlessness; mde_mi7_2 = Feelings of excessive guilt; mde_mi7_bias = Information of the domain not explained by the input variables; mde_mi8 = Diminished ability to think or concentrate, or indecisiveness+; mde_mi8_1 = Diminished ability to think or concentrate; mde_mi8_2 = Indecisiveness; mde_mi8_bias = Information of the domain not explained by the input variables; mde_mi9 = Recurrent thoughts of death; mde_bias1 = Information due to top censoring by choosing three domains in minor criteria; mde_bias2 = Information due to top censoring by choosing four domains in minor criteria; mde_bias = Information of diagnosis not explained by the domain

Assumed symptom prevalence	0.05	0.1	0.3	0.5	0.7	0.05	0.1	0.3	0.5	0.7	0.05	0.1	0.3	0.5	0.7	0.05	0.1	0.3	0.5	0.7	0.05	0.1	0.3	0.5	0.7
Assumed symptom correlations	0	0	0	0	0	0.1	0.1	0.1	0.1	0.1	0.4	0.4	0.4	0.4	0.4	0.7	0.7	0.7	0.7	0.7	0.9	0.9	0.9	0.9	0.9
mde	0 (0)	0 (0.01) *<1>	0 (0.01) *<8>	0 (0.01) *<4>	0 (0.01) *<8>	0 (0.01) *<7>	0 (0.01) *<4>	0 (0.01) *<7>	0 (0.01) *<7>	0 (0.01) *<5>	0 (0.01) *<4>	0 (0.01) *<9>	0 (0.01) *<4>	0 (0.01) *<5>	0 (0.01) *<6>	0 (0.01) *<3>	0 (0.01) *<3>	0 (0.01) *<7>	0 (0.01) *<5>	0 (0.01) *<4>	0 (0.01) *<5>	0 (0.01) *<7>	0 (0.01) *<3>	0 (0.01)	0 (0.01) *<4>
mde_ma1	0 (0.01) *<8>	0 (0.01) *<4>	0 (0.01) *<7>	0 (0.01) *<3>	0 (0.01) *<4>	0 (0.01) *<2>	0 (0.01) *<5>	0 (0.01) *<4>	0 (0.01) *<5>	0 (0.01) *<4>	0 (0.01) *<4>	0 (0.01) *<7>	0 (0.01) *<3>	0 (0.01) *<5>	0 (0.01) *<7>	0 (0.01) *<3>	0 (0.01) *<6>	0 (0.01) *<4>	0 (0.01) *<7>	0 (0.01) *<3>	0 (0.01) *<3>	0 (0.01) *<8>	0 (0.01) *<2>	0 (0.01) *<1>	0 (0.01) *<3>
mde_ma2	0 (0.01) *<12>	0 (0.01) *<5>	0 (0.01) *<3>	0 (0.01) *<5>	0 (0.01) *<8>	0 (0.01) *<3>	0 (0.01) *<3>	0 (0.01) *<3>	0 (0.01) *<6>	0 (0.01) *<7>	0 (0.01) *<3>	0 (0.01) *<5>	0 (0.01) *<4>	0 (0.01) *<7>	0 (0.01) *<6>	0 (0.01) *<5>	0 (0.01) *<4>	0 (0.01) *<5>	0 (0.01) *<4>	0 (0.01) *<4>	0 (0.01) *<2>	0 (0.01) *<5>	0 (0.01) *<1>	0 (0.01) *<2>	0 (0.01) *<3>
mde_mi3	0 (0.01) *<3>	0 (0.01) *<8>	0 (0.01) *<6>	0 (0.01) *<9>	0 (0.01) *<5>	0 (0.01) *<4>	0 (0.01) *<4>	0 (0.01) *<2>	0 (0.01) *<6>	0 (0.01) *<6>	0 (0.01) *<5>	0 (0.01) *<3>	0 (0.01) *<4>	0 (0.01) *<8>	0 (0.01) *<8>	0 (0.01) *<2>	0 (0.01) *<5>	0 (0.01) *<3>	0 (0.01) *<7>	0 (0.01) *<2>	0 (0.01) *<3>	0 (0.01) *<7>	0 (0.01) *<3>	0 (0.01) *<2>	0 (0.01) *<3>
mde_mi3_1	0 (0.01) *<2>	0 (0.01) *<3>	0 (0.01) *<6>	0 (0.01) *<5>	0 (0.01) *<4>	0 (0.01) *<7>	0 (0.01) *<5>	0 (0.01) *<6>	0 (0.01) *<3>	0 (0.01) *<6>	0 (0.01) *<7>	0 (0.01) *<7>	0 (0.01) *<3>	0 (0.01) *<7>	0 (0.01) *<5>	0 (0.01) *<4>	0 (0.01) *<6>	0 (0.01) *<3>	0 (0.01) *<6>	0 (0.01) *<6>	0 (0.01) *<4>	0 (0.01) *<6>	0 (0.01) *<2>	0 (0.01) *<3>	0 (0.01) *<2>
mde_mi3_2	0 (0.01) *<8>	0 (0.01) *<6>	0 (0.01) *<6>	0 (0.01) *<7>	0 (0.01) *<4>	0 (0.01) *<4>	0 (0.01) *<6>	0 (0.01) *<3>	0 (0.01) *<7>	0 (0.01) *<7>	0 (0.01) *<4>	0 (0.01) *<3>	0 (0.01) *<6>	0 (0.01) *<5>	0 (0.01) *<7>	0 (0.01) *<5>	0 (0.01) *<4>	0 (0.01) *<4>	0 (0.01) *<4>	0 (0.01) *<3>	0 (0.01) *<2>	0 (0.01) *<5>	0 (0.01) *<1>	0 (0.01) *<2>	0 (0.01) *<6>
mde_mi3_bias	0 (0.02) *<2>	0 (0.01) *<1>	0 (0.01) *<5>	0 (0.01) *<8>	0 (0.01) *<6>	0 (0.01) *<7>	0 (0.01) *<4>	0 (0.01) *<7>	0 (0.01) *<5>	0 (0.01) *<5>	0 (0.01) *<6>	0 (0.01) *<4>	0 (0.01) *<13>	0 (0.01) *<4>	0 (0.01) *<5>	0 (0.01) *<3>	0 (0.01) *<5>	0 (0.01) *<4>	0 (0.01) *<5>	0 (0.01) *<5>	0 (0.01)	0 (0.01) *<5>	0 (0.01) *<3>	0 (0.01) *<1>	0 (0.01) *<4>
mde_mi4	0 (0.01) *<2>	0 (0.01) *<5>	0 (0.01) *<4>	0 (0.01) *<3>	0 (0.01) *<3>	0 (0.01) *<6>	0 (0.01) *<1>	0 (0.01) *<3>	0 (0.01) *<8>	0 (0.01) *<7>	0 (0.01) *<3>	0 (0.01) *<3>	0 (0.01) *<6>	0 (0.01) *<3>	0 (0.01) *<5>	0 (0.01) *<6>	0 (0.01) *<5>	0 (0.01) *<3>	0 (0.01) *<4>	0 (0.01) *<1>	0 (0.01) *<4>	0 (0.01) *<7>	0 (0.01) *<2>	0 (0.01) *<1>	0 (0.01) *<7>
mde_mi4_1	0 (0.01) *<5>	0 (0.01) *<4>	0 (0.01) *<3>	0 (0.01) *<4>	0 (0.01) *<6>	0 (0.01) *<3>	0 (0.01) *<10>	0 (0.01) *<4>	0 (0.01) *<4>	0 (0.01) *<6>	0 (0.01) *<6>	0 (0.01) *<5>	0 (0.01) *<9>	0 (0.01) *<9>	0 (0.01) *<4>	0 (0.01) *<8>	0 (0.01) *<5>	0 (0.01) *<3>	0 (0.01) *<5>	0 (0.01) *<2>	0 (0.01) *<5>	0 (0.01) *<6>	0 (0.01) *<1>	0 (0.01) *<1>	0 (0.01) *<5>
mde_mi4_2	0 (0)	0 (0.01) *<2>	0 (0.01) *<2>	0 (0.01) *<7>	0 (0.01) *<6>	0 (0.01) *<2>	0 (0.01) *<4>	0 (0.01) *<7>	0 (0.01) *<6>	0 (0.01) *<2>	0 (0.01) *<5>	0 (0.01) *<4>	0 (0.01) *<7>	0 (0.01) *<9>	0 (0.01) *<5>	0 (0.01) *<5>	0 (0.01) *<3>	0 (0.01) *<4>	0 (0.01) *<2>	0 (0.01) *<6>	0 (0.01) *<1>	0 (0.01) *<5>	0 (0.01) *<1>	0 (0.01) *<2>	0 (0.01) *<7>
mde_mi4_bias	0 (0)	0 (0.01) *<3>	0 (0.01) *<7>	0 (0.01) *<2>	0 (0.01) *<4>	0 (0.01) *<5>	0 (0.01) *<4>	0 (0.01) *<7>	0 (0.01) *<4>	0 (0.01) *<6>	0 (0.01) *<7>	0 (0.01) *<8>	0 (0.01) *<11>	0 (0.01) *<9>	0 (0.01) *<6>	0 (0.01) *<8>	0 (0.01) *<4>	0 (0.01) *<5>	0 (0.01) *<3>	0 (0.01) *<4>	0 (0.01) *<3>	0 (0.01) *<7>	0 (0.01) *<2>	0 (0.01) *<3>	0 (0.01) *<6>
mde_mi5	0 (0.01) *<3>	0 (0.01) *<10>	0 (0.01) *<8>	0 (0.01) *<5>	0 (0.01) *<6>	0 (0.01) *<9>	0 (0.01) *<9>	0 (0.01) *<5>	0 (0.01) *<3>	0 (0.01) *<4>	0 (0.01) *<7>	0 (0.01) *<3>	0 (0.01) *<4>	0 (0.01) *<5>	0 (0.01) *<10>	0 (0.01) *<2>	0 (0.01) *<4>	0 (0.01) *<4>	0 (0.01) *<9>	0 (0.01) *<7>	0 (0.01) *<3>	0 (0.01) *<2>	0 (0.01) *<3>	0 (0.01) *<4>	0 (0.01) *<4>
mde_mi5_1	0 (0.01) *<8>	0 (0.01) *<6>	0 (0.01) *<8>	0 (0.01) *<3>	0 (0.01) *<7>	0 (0.01) *<5>	0 (0.01) *<6>	0 (0.01) *<6>	0 (0.01) *<4>	0 (0.01) *<1>	0 (0.01) *<4>	0 (0.01) *<3>	0 (0.01) *<8>	0 (0.01) *<9>	0 (0.01) *<6>	0 (0.01) *<2>	0 (0.01) *<5>	0 (0.01) *<4>	0 (0.01) *<8>	0 (0.01) *<3>	0 (0.01) *<3>	0 (0.01) *<4>	0 (0.01) *<4>	0 (0.01) *<2>	0 (0.01) *<4>
mde_mi5_2	0 (0)	0 (0.01) *<5>	0 (0.01) *<3>	0 (0.01) *<4>	0 (0.01) *<10>	0 (0.01) *<5>	0 (0.01) *<7>	0 (0.01) *<5>	0 (0.01) *<3>	0 (0.01) *<6>	0 (0.01) *<7>	0 (0.01) *<8>	0 (0.01) *<4>	0 (0.01) *<9>	0 (0.01) *<7>	0 (0.01) *<2>	0 (0.01) *<3>	0 (0.01) *<8>	0 (0.01) *<6>	0 (0.01) *<2>	0 (0.01) *<2>	0 (0.01) *<5>	0 (0.01) *<5>	0 (0.01) *<3>	0 (0.01) *<6>
mde_mi5_bias	0 (0)	0 (0.01) *<8>	0 (0.01) *<4>	0 (0.01) *<6>	0 (0.01) *<2>	0 (0.01) *<7>	0 (0.01) *<3>	0 (0.01) *<4>	0 (0.01) *<5>	0 (0.01) *<2>	0 (0.01) *<6>	0 (0.01) *<5>	0 (0.01) *<6>	0 (0.01) *<8>	0 (0.01) *<9>	0 (0.01) *<5>	0 (0.01) *<4>	0 (0.01) *<5>	0 (0.01) *<7>	0 (0.01) *<5>	0 (0.01) *<5>	0 (0.01) *<8>	0 (0.01) *<1>	0 (0.01) *<1>	0 (0.01) *<4>
mde_mi6	0 (0.01) *<5>	0 (0.01) *<1>	0 (0.01) *<9>	0 (0.01) *<2>	0 (0.01) *<5>	0 (0.01) *<5>	0 (0.01) *<2>	0 (0.01) *<2>	0 (0.01) *<1>	0 (0.01) *<5>	0 (0.01) *<2>	0 (0.01) *<8>	0 (0.01) *<5>	0 (0.01) *<7>	0 (0.01) *<6>	0 (0.01) *<1>	0 (0.01) *<1>	0 (0.01) *<3>	0 (0.01) *<6>	0 (0.01) *<4>	0 (0.01) *<3>	0 (0.01) *<5>	0 (0.01) *<4>	0 (0.01) *<4>	0 (0.01) *<2>
mde_mi6_1	0 (0.01) *<7>	0 (0.01) *<5>	0 (0.01) *<6>	0 (0.01) *<6>	0 (0.01) *<8>	0 (0.01) *<1>	0 (0.01) *<5>	0 (0.01) *<4>	0 (0.01) *<6>	0 (0.01) *<1>	0 (0.01) *<3>	0 (0.01) *<6>	0 (0.01) *<3>	0 (0.01) *<7>	0 (0.01) *<2>	0 (0.01) *<7>	0 (0.01) *<5>	0 (0.01) *<3>	0 (0.01) *<6>	0 (0.01) *<6>	0 (0.01) *<2>	0 (0.01) *<6>	0 (0.01) *<2>	0 (0.01) *<5>	0 (0.01) *<4>
mde_mi6_2	0 (0.01) *<5>	0 (0.01) *<2>	0 (0.01) *<6>	0 (0.01) *<4>	0 (0.01) *<5>	0 (0.01) *<3>	0 (0.01) *<2>	0 (0.01) *<3>	0 (0.01) *<3>	0 (0.01) *<4>	0 (0.01) *<1>	0 (0.01) *<8>	0 (0.01) *<3>	0 (0.01) *<10>	0 (0.01) *<4>	0 (0.01) *<3>	0 (0.01) *<2>	0 (0.01) *<3>	0 (0.01) *<7>	0 (0.01) *<6>	0 (0.01) *<2>	0 (0.01) *<7>	0 (0.01) *<3>	0 (0.01) *<1>	0 (0.01) *<2>
mde_mi6_bias	0 (0)	0 (0.01) *<4>	0 (0.01) *<11>	0 (0.01) *<11>	0 (0.01) *<4>	0 (0.01) *<6>	0 (0.01) *<3>	0 (0.01) *<6>	0 (0.01) *<8>	0 (0.01) *<3>	0 (0.01) *<3>	0 (0.01) *<7>	0 (0.01) *<2>	0 (0.01) *<9>	0 (0.01) *<3>	0 (0.01) *<8>	0 (0.01) *<6>	0 (0.01) *<4>	0 (0.01) *<5>	0 (0.01) *<7>	0 (0.01) *<2>	0 (0.01) *<9>	0 (0.01) *<3>	0 (0.01) *<1>	0 (0.01) *<4>
mde_mi7	0 (0.01) *<10>	0 (0.01) *<6>	0 (0.01) *<3>	0 (0.01) *<1>	0 (0.01) *<5>	0 (0.01) *<4>	0 (0.01) *<7>	0 (0.01) *<6>	0 (0.01) *<4>	0 (0.01) *<5>	0 (0.01) *<5>	0 (0.01) *<9>	0 (0.01) *<1>	0 (0.01) *<4>	0 (0.01) *<4>	0 (0.01) *<4>	0 (0.01) *<6>	0 (0.01) *<4>	0 (0.01) *<6>	0 (0.01) *<3>	0 (0.01) *<3>	0 (0.01) *<9>	0 (0.01) *<2>	0 (0.01) *<2>	0 (0.01) *<4>
mde_mi7_1	0 (0.01) *<10>	0 (0.01) *<5>	0 (0.01) *<5>	0 (0.01) *<3>	0 (0.01) *<7>	0 (0.01) *<6>	0 (0.01) *<4>	0 (0.01) *<8>	0 (0.01) *<4>	0 (0.01) *<6>	0 (0.01) *<2>	0 (0.01) *<5>	0 (0.01) *<2>	0 (0.01) *<7>	0 (0.01) *<6>	0 (0.01) *<3>	0 (0.01) *<8>	0 (0.01) *<2>	0 (0.01) *<4>	0 (0.01) *<4>	0 (0.01) *<3>	0 (0.01) *<6>	0 (0.01) *<5>	0 (0.01) *<2>	0 (0.01) *<2>
mde_mi7_2	0 (0.01) *<3>	0 (0.01) *<5>	0 (0.01) *<7>	0 (0.01) *<7>	0 (0.01) *<4>	0 (0.01) *<2>	0 (0.01) *<5>	0 (0.01) *<4>	0 (0.01) *<3>	0 (0.01) *<4>	0 (0.01) *<3>	0 (0.01) *<7>	0 (0.01) *<8>	0 (0.01) *<5>	0 (0.01) *<5>	0 (0.01) *<6>	0 (0.01) *<8>	0 (0.01) *<4>	0 (0.01) *<6>	0 (0.01) *<6>	0 (0.01) *<6>	0 (0.01) *<6>	0 (0.01) *<2>	0 (0.01) *<1>	0 (0.01) *<3>
mde_mi7_bias	0 (0)	0 (0.01) *<6>	0 (0.01) *<7>	0 (0.01) *<4>	0 (0.01) *<5>	0 (0.01) *<5>	0 (0.01) *<2>	0 (0.01) *<6>	0 (0.01) *<4>	0 (0.01) *<5>	0 (0.01) *<6>	0 (0.01) *<2>	0 (0.01) *<3>	0 (0.01) *<5>	0 (0.01) *<8>	0 (0.01) *<6>	0 (0.01) *<6>	0 (0.01) *<2>	0 (0.01) *<4>	0 (0.01) *<5>	0 (0.01) *<6>	0 (0.01) *<6>	0 (0.01) *<5>	0 (0.01) *<2>	0 (0.01) *<5>
mde_mi8	0 (0.01) *<12>	0 (0.01) *<4>	0 (0.01) *<5>	0 (0.01) *<3>	0 (0.01) *<5>	0 (0.01) *<5>	0 (0.01) *<4>	0 (0.01) *<3>	0 (0.01) *<4>	0 (0.01) *<5>	0 (0.01) *<2>	0 (0.01) *<7>	0 (0.01) *<6>	0 (0.01) *<7>	0 (0.01) *<3>	0 (0.01) *<6>	0 (0.01) *<6>	0 (0.01) *<3>	0 (0.01) *<8>	0 (0.01) *<5>	0 (0.01) *<4>	0 (0.01) *<4>	0 (0.01) *<4>	0 (0.01) *<2>	0 (0.01) *<5>
mde_mi8_1	0 (0.01) *<8>	0 (0.01) *<6>	0 (0.01) *<4>	0 (0.01) *<8>	0 (0.01) *<6>	0 (0.01) *<7>	0 (0.01) *<4>	0 (0.01) *<4>	0 (0.01) *<5>	0 (0.01) *<4>	0 (0.01) *<1>	0 (0.01) *<9>	0 (0.01) *<7>	0 (0.01) *<7>	0 (0.01) *<2>	0 (0.01) *<10>	0 (0.01) *<6>	0 (0.01) *<4>	0 (0.01) *<6>	0 (0.01) *<5>	0 (0.01) *<4>	0 (0.01) *<5>	0 (0.01) *<5>	0 (0.01) *<2>	0 (0.01) *<5>
mde_mi8_2	0 (0.01) *<8>	0 (0.01) *<2>	0 (0.01) *<5>	0 (0.01) *<5>	0 (0.01) *<2>	0 (0.01) *<5>	0 (0.01) *<5>	0 (0.01) *<4>	0 (0.01) *<5>	0 (0.01) *<7>	0 (0.01) *<5>	0 (0.01) *<6>	0 (0.01) *<9>	0 (0.01) *<2>	0 (0.01) *<3>	0 (0.01) *<5>	0 (0.01) *<9>	0 (0.01) *<4>	0 (0.01) *<8>	0 (0.01) *<5>	0 (0.01) *<2>	0 (0.01) *<3>	0 (0.01) *<2>	0 (0.01) *<2>	0 (0.01) *<8>
mde_mi8_bias	0 (0)	0 (0.01) *<2>	0 (0.01) *<2>	0 (0.01) *<3>	0 (0.01) *<6>	0 (0.01) *<8>	0 (0.01) *<2>	0 (0.01) *<3>	0 (0.01) *<1>	0 (0.01) *<7>	0 (0.01) *<1>	0 (0.01) *<3>	0 (0.01) *<5>	0 (0.01) *<6>	0 (0.01) *<6>	0 (0.01) *<5>	0 (0.01) *<4>	0 (0.01) *<4>	0 (0.01) *<5>	0 (0.01) *<8>	0 (0.01) *<9>	0 (0.01) *<4>	0 (0.01) *<2>	0 (0.01) *<2>	0 (0.01) *<6>
mde_mi9	0 (0.01) *<12>	0 (0.01) *<4>	0 (0.01) *<5>	0 (0.01) *<2>	0 (0.01) *<6>	0 (0.01) *<3>	0 (0.01) *<6>	0 (0.01) *<3>	0 (0.01) *<4>	0 (0.01) *<5>	0 (0.01) *<5>	0 (0.01) *<6>	0 (0.01) *<5>	0 (0.01) *<9>	0 (0.01) *<3>	0 (0.01) *<6>	0 (0.01) *<9>	0 (0.01) *<6>	0 (0.01) *<5>	0 (0.01) *<4>	0 (0.01) *<3>	0 (0.01) *<5>	0 (0.01) *<1>	0 (0.01) *<3>	0 (0.01) *<5>
mde_bias1	0 (0.01) *<8>	0 (0.01) *<4>	0 (0.01) *<9>	0 (0.01) *<7>	0 (0.01) *<7>	0 (0.01) *<6>	0 (0.01) *<4>	0 (0.01) *<1>	0 (0.01) *<2>	0 (0.01) *<6>	0 (0.01) *<2>	0 (0.01) *<5>	0 (0.01) *<6>	0 (0.01) *<8>	0 (0.01) *<7>	0 (0.01) *<4>	0 (0.01) *<5>	0 (0.01) *<4>	0 (0.01) *<5>	0 (0.01) *<3>	0 (0.01) *<4>	0 (0.01) *<6>	0 (0.01) *<2>	0 (0.01) *<3>	0 (0.01) *<2>
mde_bias2	0 (0.01) *<2>	0 (0.01) *<5>	0 (0.01) *<8>	0 (0.01) *<5>	0 (0.01) *<5>	0 (0.01) *<6>	0 (0.01) *<4>	0 (0.01) *<2>	0 (0.01) *<3>	0 (0.01) *<6>	0 (0.01) *<3>	0 (0.01) *<5>	0 (0.01) *<5>	0 (0.01) *<8>	0 (0.01) *<7>	0 (0.01) *<4>	0 (0.01) *<5>	0 (0.01) *<3>	0 (0.01) *<6>	0 (0.01) *<3>	0 (0.01) *<4>	0 (0.01) *<4>	0 (0.01) *<2>	0 (0.01) *<3>	0 (0.01) *<2>
mde_bias	0 (0.01) *<8>	0 (0.01) *<4>	0 (0.01) *<3>	0 (0.01) *<4>	0 (0.01) *<8>	0 (0.01) *<7>	0 (0.01) *<4>	0 (0.01) *<3>	0 (0.01) *<1>	0 (0.01) *<4>	0 (0.01) *<4>	0 (0.01) *<5>	0 (0.01) *<6>	0 (0.01) *<10>	0 (0.01) *<8>	0 (0.01) *<5>	0 (0.01) *<4>	0 (0.01) *<4>	0 (0.01) *<7>	0 (0.01) *<3>	0 (0.01) *<3>	0 (0.01) *<6>	0 (0.01) *<3>	0 (0.01) *<2>	0 (0.01) *<2>

**Table 16:** Correlations between manic episodes and the input symptoms of dysthymic disorder by assumed symptom prevalence and correlations Mean (standard deviation) *<number> = the numbers of significant statistics (p < 0.05) in 100 simulations !# = p < 0.001 in all of the 100 simulations !@ = p < 0.001 in some of the 100 simulations Note: See Table 1 for variable definitions and Table 2 for the prevalence of major depressive episodes, dysthymic disorder, and manic episodes. dys = Dysthymic Disorder; dys_ma = Depressed mood most of the day for more days than not, for at least 2 years; dys_mi = Minor criteria (at least 2 items); dys_mi1 = Poor appetite or overeating; dys_mi1_1 = Poor appetite; dys_mi1_2 = Overeating; dys_mi1_bias = Information of the domain not explained by the input variables; dys_mi4 = Low self-esteem; dys_mi6 = Feelings of hopelessness; dys_mi_bias = Information of minor criteria not explained by input variables; dys_bias = Information of diagnosis not explained by major or minor criteria

Assumed symptom prevalence	0.05	0.1	0.3	0.5	0.7	0.05	0.1	0.3	0.5	0.7	0.05	0.1	0.3	0.5	0.7	0.05	0.1	0.3	0.5	0.7	0.05	0.1	0.3	0.5	0.7
Assumed symptom correlations	0	0	0	0	0	0.1	0.1	0.1	0.1	0.1	0.4	0.4	0.4	0.4	0.4	0.7	0.7	0.7	0.7	0.7	0.9	0.9	0.9	0.9	0.9
dys	0 (0)	0 (0.01) *<4>	0 (0.01) *<2>	0 (0.01) *<2>	0 (0.01) *<6>	0 (0.01) *<1>	0 (0.01) *<2>	0 (0.01) *<4>	0 (0.01) *<7>	0 (0.01) *<5>	0 (0.01) *<6>	0 (0.01) *<4>	0 (0.01) *<12>	0 (0.01) *<5>	0 (0.01) *<6>	0 (0.01) *<2>	0 (0.01) *<6>	0 (0.01) *<6>	0 (0.01) *<9>	0 (0.01) *<6>	0 (0.01) *<4>	0 (0.01) *<4>	0 (0.01) *<3>	0 (0.01) *<3>	0 (0.01) *<3>
dys_ma	0 (0.01) *<8>	0 (0.01) *<4>	0 (0.01) *<3>	0 (0.01) *<2>	0 (0.01) *<6>	0 (0.01) *<2>	0 (0.01) *<4>	0 (0.01) *<5>	0 (0.01) *<7>	0 (0.01) *<5>	0 (0.01) *<8>	0 (0.01) *<3>	0 (0.01) *<9>	0 (0.01) *<4>	0 (0.01) *<6>	0 (0.01) *<3>	0 (0.01) *<5>	0 (0.01) *<6>	0 (0.01) *<6>	0 (0.01) *<6>	0 (0.01) *<5>	0 (0.01) *<4>	0 (0.01) *<2>	0 (0.01) *<1>	0 (0.01) *<1>
dys_mi	0 (0.01) *<8>	0 (0.01) *<5>	0 (0.01) *<4>	0 (0.01) *<6>	0 (0.01) *<2>	0 (0.01) *<9>	0 (0.01) *<5>	0 (0.01) *<6>	0 (0.01) *<6>	0 (0.01) *<6>	0 (0.01) *<4>	0 (0.01) *<8>	0 (0.01) *<5>	0 (0.01) *<5>	0 (0.01) *<3>	0 (0.01) *<4>	0 (0.01) *<5>	0 (0.01) *<5>	0 (0.01) *<6>	0 (0.01) *<3>	0 (0.01) *<5>	0 (0.01) *<4>	0 (0.01) *<4>	0 (0.01) *<2>	0 (0.01) *<2>
dys_mi1	0 (0.01) *<12>	0 (0.01) *<2>	0 (0.01) *<6>	0 (0.01) *<7>	0 (0.01) *<3>	0 (0.01) *<6>	0 (0.01) *<2>	0 (0.01) *<6>	0 (0.01) *<5>	0 (0.01) *<6>	0 (0.01) *<5>	0 (0.01) *<4>	0 (0.01) *<5>	0 (0.01) *<3>	0 (0.01) *<10>	0 (0.01) *<6>	0 (0.01) *<5>	0 (0.01) *<5>	0 (0.01) *<3>	0 (0.01) *<5>	0 (0.01) *<1>	0 (0.01) *<7>	0 (0.01) *<4>	0 (0.01) *<6>	0 (0.01) *<3>
dys_mi1_1	0 (0.01) *<12>	0 (0.01) *<4>	0 (0.01) *<8>	0 (0.01) *<2>	0 (0.01) *<3>	0 (0.01) *<4>	0 (0.01) *<6>	0 (0.01) *<3>	0 (0.01) *<3>	0 (0.01) *<6>	0 (0.01) *<5>	0 (0.01) *<4>	0 (0.01) *<9>	0 (0.01) *<5>	0 (0.01) *<9>	0 (0.01) *<6>	0 (0.01) *<5>	0 (0.01) *<6>	0 (0.01) *<5>	0 (0.01) *<3>	0 (0.01) *<2>	0 (0.01) *<4>	0 (0.01) *<4>	0 (0.01) *<6>	0 (0.01) *<2>
dys_mi1_2	0 (0.01) *<7>	0 (0.01) *<2>	0 (0.01) *<3>	0 (0.01) *<8>	0 (0.01) *<1>	0 (0.01) *<3>	0 (0.01) *<4>	0 (0.01) *<3>	0 (0.01) *<11>	0 (0.01) *<6>	0 (0.01) *<4>	0 (0.01) *<8>	0 (0.01) *<4>	0 (0.01) *<1>	0 (0.01) *<10>	0 (0.01) *<5>	0 (0.01) *<4>	0 (0.01) *<5>	0 (0.01) *<5>	0 (0.01) *<4>	0 (0.01) *<2>	0 (0.01) *<5>	0 (0.01) *<3>	0 (0.01) *<3>	0 (0.01) *<3>
dys_mi1_bias	0 (0)	0 (0.01) *<2>	0 (0.01) *<2>	0 (0.01) *<3>	0 (0.01) *<4>	0 (0.01) *<5>	0 (0.01) *<4>	0 (0.01) *<4>	0 (0.01) *<5>	0 (0.01) *<6>	0 (0.01) *<4>	0 (0.01) *<5>	0 (0.01) *<7>	0 (0.01) *<4>	0 (0.01) *<10>	0 (0.01) *<9>	0 (0.01) *<5>	0 (0.01) *<7>	0 (0.01) *<8>	0 (0.01) *<7>	0 (0.01) *<1>	0 (0.01) *<10>	0 (0.01) *<2>	0 (0.01) *<2>	0 (0.01) *<3>
dys_mi4	0 (0.01) *<8>	0 (0.01) *<8>	0 (0.01) *<4>	0 (0.01) *<3>	0 (0.01) *<7>	0 (0.01) *<5>	0 (0.01) *<3>	0 (0.01) *<5>	0 (0.01) *<6>	0 (0.01) *<5>	0 (0.01) *<4>	0 (0.01) *<8>	0 (0.01) *<9>	0 (0.01) *<5>	0 (0.01) *<6>	0 (0.01) *<5>	0 (0.01) *<3>	0 (0.01) *<6>	0 (0.01) *<4>	0 (0.01) *<4>	0 (0.01) *<2>	0 (0.01) *<2>	0 (0.01) *<4>	0 (0.01) *<4>	0 (0.01) *<2>
dys_mi6	0 (0.01) *<5>	0 (0.01) *<3>	0 (0.01) *<4>	0 (0.01) *<5>	0 (0.01) *<4>	0 (0.01) *<5>	0 (0.01) *<4>	0 (0.01) *<3>	0 (0.01) *<5>	0 (0.01) *<6>	0 (0.01) *<6>	0 (0.01) *<6>	0 (0.01) *<5>	0 (0.01) *<3>	0 (0.01) *<4>	0 (0.01) *<6>	0 (0.01) *<7>	0 (0.01) *<7>	0 (0.01) *<1>	0 (0.01) *<2>	0 (0.01) *<5>	0 (0.01) *<5>	0 (0.01) *<3>	0 (0.01) *<2>	0 (0.01) *<1>
dys_mi_bias	0 (0.01)	0 (0.01) *<9>	0 (0.01) *<4>	0 (0.01) *<4>	0 (0.01) *<3>	0 (0.01) *<6>	0 (0.01) *<6>	0 (0.01) *<2>	0 (0.01) *<5>	0 (0.01) *<4>	0 (0.01) *<2>	0 (0.01) *<6>	0 (0.01) *<8>	0 (0.01) *<6>	0 (0.01) *<6>	0 (0.01) *<5>	0 (0.01) *<4>	0 (0.01) *<2>	0 (0.01) *<6>	0 (0.01) *<3>	0 (0.01) *<1>	0 (0.01) *<7>	0 (0.01) *<2>	0 (0.01) *<3>	0 (0.01) *<3>
dys_bias	0 (0.01) *<10>	0 (0.01) *<3>	0 (0.01) *<6>	0 (0.01) *<3>	0 (0.01) *<3>	0 (0.01) *<6>	0 (0.01) *<2>	0 (0.01) *<6>	0 (0.01) *<6>	0 (0.01) *<5>	0 (0.01) *<3>	0 (0.01) *<7>	0 (0.01) *<4>	0 (0.01) *<4>	0 (0.01) *<4>	0 (0.01) *<5>	0 (0.01) *<6>	0 (0.01) *<5>	0 (0.01) *<4>	0 (0.01) *<4>	0 (0.01) *<4>	0 (0.01) *<4>	0 (0.01) *<4>	0 (0.01) *<3>	0 (0.01) *<3>

**Table 17:** Correlations between manic episodes and their input symptoms by assumed symptom prevalence and correlations Mean (standard deviation) *<number> = the numbers of significant statistics (p < 0.05) in 100 simulations !# = p < 0.001 in all of the 100 simulations !@ = p < 0.001 in some of the 100 simulations manic = Manic episodes; man_ma1 = Elevated mood, lasting at least 1 week; man_ma2 = Expansive mood, lasting at least 1 week; man_ma3 = Irritable mood, lasting at least 1 week; man_mi1 = Increased self-esteem or grandiosity; man_mi1_1 = Increased self-esteem; man_mi1_2 = Grandiosity; man_mi1_bias = Information of the domain not explained by the input variables; man_mi2 = Decreased need for sleep (e.g., feels rested after only 3 hours of sleep); man_mi3 = More talkative than usual or pressure to keep talking; man_mi3_1 = More talkative than usual; man_mi3_2 = Pressure to keep talking; man_mi3_bias = Information of the domain not explained by the input variables; man_mi4 = Flight of ideas or subjective experience that thoughts are racing; man_mi4_1 = Flight of ideas; man_mi4_2 = Subjective experience that thoughts are racing; man_mi4_bias = Information of the domain not explained by the input variables; man_mi5 = Distractibility (i.e., attention too easily drawn to unimportant or irrelevant external stimuli); man_mi6 = Increase in goal-directed activity (either socially, at work or school, or sexually) or psychomotor agitation; man_mi6_1 = Increase in goal-directed activity ; man_mi6_2 = Psychomotor agitation; man_mi6_bias = Information of the domain not explained by the input variables; man_mi7 = Excessive involvement in pleasurable activities that have a high potential for painful consequences (e.g., engaging in unrestrained buying sprees, sexual indiscretions, or foolish business investments); man_bias1 = Information of diagnosis due to top-censoring for choosing at least three symptoms; man_bias2 = Information of diagnosis due to top-censoring for choosing at least four symptoms; man_bias = Information of diagnosis not explained by symptoms

Assumed symptom prevalence	0.05	0.1	0.3	0.5	0.7	0.05	0.1	0.3	0.5	0.7	0.05	0.1	0.3	0.5	0.7	0.05	0.1	0.3	0.5	0.7	0.05	0.1	0.3	0.5	0.7
Assumed symptom correlations	0	0	0	0	0	0.1	0.1	0.1	0.1	0.1	0.4	0.4	0.4	0.4	0.4	0.7	0.7	0.7	0.7	0.7	0.9	0.9	0.9	0.9	0.9
manic	1 (0) *<100>	1 (0) *<100>!#	1 (0) *<100>!#	1 (0) *<100>!#	1 (0) *<100>!#	1 (0) *<100>!#	1 (0) *<100>!#	1 (0) *<100>!#	1 (0) *<100>!#	1 (0) *<100>!#	1 (0) *<100>!#	1 (0) *<100>!#	1 (0) *<100>!#	1 (0) *<100>!#	1 (0) *<100>!#	1 (0) *<100>!#	1 (0) *<100>!#	1 (0) *<100>!#	1 (0) *<100>!#	1 (0) *<100>!#	1 (0) *<100>!#	1 (0) *<100>!#	1 (0) *<100>!#	1 (0) *<100>!#	1 (0) *<100>!#
man_ma1	0.01 (0.02) *<39>	0.03 (0.02) *<73>	0.05 (0.01) *<99>	0 (0.01) *<7>	-0.01 (0.01) *<15>	0.13 (0.02) *<100>!#	0.14 (0.02) *<100>!#	0.16 (0.01) *<100>!#	0.15 (0.01) *<100>!#	0.12 (0.01) *<100>!#	0.45 (0.02) *<100>!#	0.46 (0.02) *<100>!#	0.46 (0.01) *<100>!#	0.46 (0.01) *<100>!#	0.45 (0.01) *<100>!#	0.73 (0.02) *<100>!#	0.73 (0.01) *<100>!#	0.73 (0.01) *<100>!#	0.73 (0.01) *<100>!#	0.73 (0.01) *<100>!#	0.85 (0.01) *<100>!#	0.86 (0.01) *<100>!#	0.87 (0.01) *<100>!#	0.87 (0.01) *<100>!#	0.87 (0.01) *<100>!#
man_ma2	0.02 (0.02) *<46>	0.03 (0.01) *<77>	0.05 (0.01) *<99>	0 (0.01) *<4>	0 (0.01) *<9>	0.13 (0.02) *<100>!#	0.15 (0.02) *<100>!#	0.16 (0.01) *<100>!#	0.15 (0.01) *<100>!#	0.12 (0.01) *<100>!#	0.45 (0.02) *<100>!#	0.46 (0.02) *<100>!#	0.46 (0.01) *<100>!#	0.46 (0.01) *<100>!#	0.45 (0.01) *<100>!#	0.74 (0.02) *<100>!#	0.73 (0.01) *<100>!#	0.73 (0.01) *<100>!#	0.73 (0.01) *<100>!#	0.73 (0.01) *<100>!#	0.85 (0.01) *<100>!#	0.86 (0.01) *<100>!#	0.87 (0.01) *<100>!#	0.88 (0.01) *<100>!#	0.87 (0.01) *<100>!#
man_ma3	0.05 (0.01) *<100>	0.14 (0.01) *<100>!#	0.6 (0.01) *<100>!#	0.88 (0) *<100>!#	0.98 (0) *<100>!#	0.37 (0.02) *<100>!#	0.45 (0.01) *<100>!#	0.7 (0.01) *<100>!#	0.86 (0) *<100>!#	0.95 (0) *<100>!#	0.75 (0.01) *<100>!#	0.77 (0.01) *<100>!#	0.84 (0.01) *<100>!#	0.89 (0) *<100>!#	0.92 (0) *<100>!#	0.91 (0.01) *<100>!#	0.91 (0.01) *<100>!#	0.93 (0) *<100>!#	0.94 (0) *<100>!#	0.95 (0) *<100>!#	0.96 (0.01) *<100>!#	0.96 (0) *<100>!#	0.97 (0) *<100>!#	0.97 (0) *<100>!#	0.97 (0) *<100>!#
man_mi1	0.02 (0.02) *<49>	0.05 (0.01) *<99>	0.13 (0.01) *<100>!#	0.08 (0.01) *<100>!#	0.02 (0.01) *<57>	0.18 (0.02) *<100>!#	0.2 (0.01) *<100>!#	0.24 (0.01) *<100>!#	0.22 (0.01) *<100>!#	0.16 (0.01) *<100>!#	0.49 (0.02) *<100>!#	0.5 (0.01) *<100>!#	0.51 (0.01) *<100>!#	0.5 (0.01) *<100>!#	0.48 (0.01) *<100>!#	0.74 (0.02) *<100>!#	0.74 (0.01) *<100>!#	0.74 (0.01) *<100>!#	0.74 (0.01) *<100>!#	0.74 (0.01) *<100>!#	0.85 (0.01) *<100>!#	0.86 (0.01) *<100>!#	0.87 (0) *<100>!#	0.87 (0) *<100>!#	0.87 (0.01) *<100>!#
man_mi1_1	0.02 (0.02) *<46>	0.04 (0.02) *<81>	0.08 (0.01) *<100>!#	0.05 (0.01) *<100>	0.01 (0.01) *<19>	0.15 (0.02) *<100>!#	0.16 (0.02) *<100>!#	0.19 (0.01) *<100>!#	0.18 (0.01) *<100>!#	0.14 (0.01) *<100>!#	0.46 (0.02) *<100>!#	0.47 (0.02) *<100>!#	0.48 (0.01) *<100>!#	0.48 (0.01) *<100>!#	0.46 (0.01) *<100>!#	0.74 (0.02) *<100>!#	0.74 (0.01) *<100>!#	0.74 (0.01) *<100>!#	0.74 (0.01) *<100>!#	0.74 (0.01) *<100>!#	0.86 (0.01) *<100>!#	0.86 (0.01) *<100>!#	0.88 (0.01) *<100>!#	0.88 (0) *<100>!#	0.88 (0) *<100>!#
man_mi1_2	0.01 (0.02) *<25>	0.04 (0.02) *<83>	0.08 (0.01) *<100>!#	0.05 (0.01) *<100>	0.01 (0.01) *<18>	0.14 (0.03) *<100>!#	0.16 (0.02) *<100>!#	0.19 (0.01) *<100>!#	0.18 (0.01) *<100>!#	0.14 (0.01) *<100>!#	0.47 (0.02) *<100>!#	0.47 (0.02) *<100>!#	0.48 (0.01) *<100>!#	0.48 (0.01) *<100>!#	0.46 (0.01) *<100>!#	0.74 (0.02) *<100>!#	0.74 (0.01) *<100>!#	0.74 (0.01) *<100>!#	0.74 (0.01) *<100>!#	0.74 (0.01) *<100>!#	0.86 (0.01) *<100>!#	0.86 (0.01) *<100>!#	0.88 (0.01) *<100>!#	0.88 (0) *<100>!#	0.88 (0.01) *<100>!#
man_mi1_bias	0 (0.02) *<3>	-0.01 (0.02) *<21>	-0.04 (0.01) *<97>	-0.03 (0.01) *<71>	-0.01 (0.01) *<6>	-0.13 (0.04) *<100>!#	-0.15 (0.03) *<100>!#	-0.17 (0.01) *<100>!#	-0.17 (0.01) *<100>!#	-0.15 (0.01) *<100>!#	-0.49 (0.03) *<100>!#	-0.49 (0.02) *<100>!#	-0.49 (0.01) *<100>!#	-0.49 (0.01) *<100>!#	-0.49 (0.01) *<100>!#	-0.76 (0.02) *<100>!#	-0.75 (0.01) *<100>!#	-0.75 (0.01) *<100>!#	-0.76 (0.01) *<100>!#	-0.76 (0.01) *<100>!#	-0.87 (0.01) *<100>!#	-0.87 (0.01) *<100>!#	-0.88 (0.01) *<100>!#	-0.89 (0) *<100>!#	-0.88 (0) *<100>!#
man_mi2	0.02 (0.02) *<47>	0.04 (0.02) *<92>	0.12 (0.01) *<100>!#	0.08 (0.01) *<100>!#	0.02 (0.01) *<50>	0.16 (0.02) *<100>!#	0.18 (0.02) *<100>!#	0.22 (0.01) *<100>!#	0.2 (0.01) *<100>!#	0.15 (0.01) *<100>!#	0.48 (0.02) *<100>!#	0.48 (0.02) *<100>!#	0.49 (0.01) *<100>!#	0.49 (0.01) *<100>!#	0.47 (0.01) *<100>!#	0.74 (0.02) *<100>!#	0.74 (0.01) *<100>!#	0.74 (0.01) *<100>!#	0.75 (0.01) *<100>!#	0.74 (0.01) *<100>!#	0.86 (0.01) *<100>!#	0.87 (0.01) *<100>!#	0.88 (0.01) *<100>!#	0.88 (0) *<100>!#	0.88 (0.01) *<100>!#
man_mi3	0.02 (0.02) *<61>	0.05 (0.01) *<96>	0.13 (0.01) *<100>!#	0.09 (0.01) *<100>!#	0.02 (0.01) *<50>	0.18 (0.02) *<100>!#	0.2 (0.01) *<100>!#	0.24 (0.01) *<100>!#	0.22 (0.01) *<100>!#	0.16 (0.01) *<100>!#	0.49 (0.02) *<100>!#	0.5 (0.01) *<100>!#	0.51 (0.01) *<100>!#	0.5 (0.01) *<100>!#	0.48 (0.01) *<100>!#	0.74 (0.02) *<100>!#	0.74 (0.01) *<100>!#	0.74 (0.01) *<100>!#	0.74 (0.01) *<100>!#	0.74 (0.01) *<100>!#	0.86 (0.01) *<100>!#	0.86 (0.01) *<100>!#	0.87 (0) *<100>!#	0.87 (0) *<100>!#	0.87 (0.01) *<100>!#
man_mi3_1	0.01 (0.02) *<37>	0.03 (0.02) *<83>	0.08 (0.01) *<100>!#	0.05 (0.01) *<100>	0.01 (0.01) *<14>	0.14 (0.02) *<100>!#	0.16 (0.02) *<100>!#	0.19 (0.01) *<100>!#	0.18 (0.01) *<100>!#	0.14 (0.01) *<100>!#	0.46 (0.02) *<100>!#	0.47 (0.02) *<100>!#	0.48 (0.01) *<100>!#	0.48 (0.01) *<100>!#	0.46 (0.01) *<100>!#	0.74 (0.02) *<100>!#	0.74 (0.01) *<100>!#	0.74 (0.01) *<100>!#	0.74 (0.01) *<100>!#	0.74 (0.01) *<100>!#	0.86 (0.01) *<100>!#	0.86 (0.01) *<100>!#	0.88 (0.01) *<100>!#	0.88 (0.01) *<100>!#	0.88 (0) *<100>!#
man_mi3_2	0.02 (0.02) *<47>	0.03 (0.02) *<77>	0.08 (0.01) *<100>!#	0.05 (0.01) *<99>	0.01 (0.01) *<16>	0.14 (0.03) *<100>!#	0.16 (0.02) *<100>!#	0.19 (0.01) *<100>!#	0.18 (0.01) *<100>!#	0.14 (0.01) *<100>!#	0.46 (0.02) *<100>!#	0.47 (0.02) *<100>!#	0.48 (0.01) *<100>!#	0.48 (0.01) *<100>!#	0.46 (0.01) *<100>!#	0.74 (0.02) *<100>!#	0.74 (0.01) *<100>!#	0.74 (0.01) *<100>!#	0.74 (0.01) *<100>!#	0.74 (0.01) *<100>!#	0.86 (0.01) *<100>!#	0.86 (0.01) *<100>!#	0.88 (0) *<100>!#	0.88 (0.01) *<100>!#	0.88 (0.01) *<100>!#
man_mi3_bias	0 (0.03) *<3>	-0.01 (0.02) *<22>	-0.04 (0.01) *<98>	-0.03 (0.01) *<82>	-0.01 (0.01) *<13>	-0.13 (0.04) *<100>!#	-0.15 (0.02) *<100>!#	-0.17 (0.01) *<100>!#	-0.17 (0.01) *<100>!#	-0.15 (0.01) *<100>!#	-0.49 (0.03) *<100>!#	-0.49 (0.02) *<100>!#	-0.49 (0.01) *<100>!#	-0.49 (0.01) *<100>!#	-0.49 (0.01) *<100>!#	-0.76 (0.02) *<100>!#	-0.75 (0.01) *<100>!#	-0.75 (0.01) *<100>!#	-0.76 (0.01) *<100>!#	-0.76 (0.01) *<100>!#	-0.87 (0.01) *<100>!#	-0.87 (0.01) *<100>!#	-0.88 (0.01) *<100>!#	-0.89 (0) *<100>!#	-0.88 (0) *<100>!#
man_mi4	0.02 (0.02) *<56>	0.05 (0.01) *<99>	0.13 (0.01) *<100>!#	0.08 (0.01) *<100>!#	0.02 (0.01) *<39>	0.17 (0.02) *<100>!#	0.2 (0.01) *<100>!#	0.24 (0.01) *<100>!#	0.22 (0.01) *<100>!#	0.16 (0.01) *<100>!#	0.49 (0.02) *<100>!#	0.5 (0.01) *<100>!#	0.51 (0.01) *<100>!#	0.5 (0.01) *<100>!#	0.48 (0.01) *<100>!#	0.75 (0.01) *<100>!#	0.74 (0.01) *<100>!#	0.74 (0.01) *<100>!#	0.74 (0.01) *<100>!#	0.74 (0.01) *<100>!#	0.86 (0.01) *<100>!#	0.86 (0.01) *<100>!#	0.87 (0) *<100>!#	0.87 (0) *<100>!#	0.87 (0) *<100>!#
man_mi4_1	0.01 (0.02) *<36>	0.04 (0.02) *<87>	0.09 (0.01) *<100>!#	0.05 (0.01) *<99>	0.01 (0.01) *<9>	0.14 (0.02) *<100>!#	0.16 (0.02) *<100>!#	0.19 (0.01) *<100>!#	0.18 (0.01) *<100>!#	0.14 (0.01) *<100>!#	0.47 (0.02) *<100>!#	0.47 (0.02) *<100>!#	0.48 (0.01) *<100>!#	0.48 (0.01) *<100>!#	0.46 (0.01) *<100>!#	0.74 (0.02) *<100>!#	0.74 (0.01) *<100>!#	0.74 (0.01) *<100>!#	0.74 (0.01) *<100>!#	0.74 (0.01) *<100>!#	0.86 (0.01) *<100>!#	0.86 (0.01) *<100>!#	0.88 (0) *<100>!#	0.88 (0) *<100>!#	0.88 (0.01) *<100>!#
man_mi4_2	0.02 (0.02) *<39>	0.03 (0.02) *<75>	0.08 (0.01) *<100>!#	0.05 (0.01) *<100>	0.01 (0.01) *<17>	0.14 (0.02) *<100>!#	0.16 (0.02) *<100>!#	0.19 (0.01) *<100>!#	0.18 (0.01) *<100>!#	0.13 (0.01) *<100>!#	0.46 (0.02) *<100>!#	0.47 (0.02) *<100>!#	0.48 (0.01) *<100>!#	0.48 (0.01) *<100>!#	0.46 (0.01) *<100>!#	0.74 (0.02) *<100>!#	0.73 (0.01) *<100>!#	0.74 (0.01) *<100>!#	0.74 (0.01) *<100>!#	0.74 (0.01) *<100>!#	0.86 (0.01) *<100>!#	0.86 (0.01) *<100>!#	0.88 (0.01) *<100>!#	0.88 (0.01) *<100>!#	0.88 (0.01) *<100>!#
man_mi4_bias	0 (0.02) *<3>	-0.01 (0.02) *<23>	-0.04 (0.01) *<97>	-0.03 (0.01) *<74>	-0.01 (0.01) *<15>	-0.13 (0.04) *<100>!#	-0.15 (0.03) *<100>!#	-0.17 (0.01) *<100>!#	-0.17 (0.01) *<100>!#	-0.15 (0.01) *<100>!#	-0.49 (0.02) *<100>!#	-0.49 (0.02) *<100>!#	-0.49 (0.01) *<100>!#	-0.49 (0.01) *<100>!#	-0.49 (0.01) *<100>!#	-0.76 (0.02) *<100>!#	-0.75 (0.01) *<100>!#	-0.75 (0.01) *<100>!#	-0.76 (0.01) *<100>!#	-0.76 (0.01) *<100>!#	-0.87 (0.01) *<100>!#	-0.87 (0.01) *<100>!#	-0.88 (0) *<100>!#	-0.89 (0) *<100>!#	-0.88 (0.01) *<100>!#
man_mi5	0.02 (0.03) *<46>	0.05 (0.02) *<95>	0.12 (0.01) *<100>!#	0.08 (0.01) *<100>!#	0.02 (0.01) *<49>	0.16 (0.03) *<100>!#	0.18 (0.02) *<100>!#	0.21 (0.01) *<100>!#	0.2 (0.01) *<100>!#	0.15 (0.01) *<100>!#	0.48 (0.02) *<100>!#	0.48 (0.02) *<100>!#	0.49 (0.01) *<100>!#	0.49 (0.01) *<100>!#	0.47 (0.01) *<100>!#	0.75 (0.02) *<100>!#	0.74 (0.01) *<100>!#	0.74 (0.01) *<100>!#	0.74 (0.01) *<100>!#	0.74 (0.01) *<100>!#	0.86 (0.01) *<100>!#	0.87 (0.01) *<100>!#	0.88 (0.01) *<100>!#	0.88 (0.01) *<100>!#	0.88 (0.01) *<100>!#
man_mi6	0.02 (0.02) *<63>	0.05 (0.01) *<100>	0.13 (0.01) *<100>!#	0.08 (0.01) *<100>!#	0.02 (0.01) *<46>	0.17 (0.02) *<100>!#	0.2 (0.01) *<100>!#	0.24 (0.01) *<100>!#	0.22 (0.01) *<100>!#	0.16 (0.01) *<100>!#	0.49 (0.02) *<100>!#	0.5 (0.01) *<100>!#	0.51 (0.01) *<100>!#	0.5 (0.01) *<100>!#	0.48 (0.01) *<100>!#	0.74 (0.02) *<100>!#	0.74 (0.01) *<100>!#	0.74 (0.01) *<100>!#	0.74 (0.01) *<100>!#	0.74 (0.01) *<100>!#	0.85 (0.01) *<100>!#	0.86 (0.01) *<100>!#	0.87 (0.01) *<100>!#	0.87 (0) *<100>!#	0.87 (0.01) *<100>!#
man_mi6_1	0.02 (0.02) *<51>	0.03 (0.01) *<84>	0.08 (0.01) *<100>!#	0.05 (0.01) *<99>	0.01 (0.01) *<18>	0.14 (0.03) *<100>!#	0.16 (0.02) *<100>!#	0.19 (0.01) *<100>!#	0.18 (0.01) *<100>!#	0.14 (0.01) *<100>!#	0.47 (0.03) *<100>!#	0.47 (0.02) *<100>!#	0.48 (0.01) *<100>!#	0.48 (0.01) *<100>!#	0.46 (0.01) *<100>!#	0.74 (0.02) *<100>!#	0.74 (0.01) *<100>!#	0.74 (0.01) *<100>!#	0.74 (0.01) *<100>!#	0.74 (0.01) *<100>!#	0.86 (0.01) *<100>!#	0.86 (0.01) *<100>!#	0.88 (0) *<100>!#	0.88 (0) *<100>!#	0.88 (0.01) *<100>!#
man_mi6_2	0.01 (0.02) *<37>	0.04 (0.01) *<88>	0.08 (0.01) *<100>!#	0.05 (0.01) *<100>	0.01 (0.01) *<14>	0.14 (0.02) *<100>!#	0.16 (0.02) *<100>!#	0.19 (0.01) *<100>!#	0.18 (0.01) *<100>!#	0.14 (0.01) *<100>!#	0.46 (0.02) *<100>!#	0.47 (0.01) *<100>!#	0.48 (0.01) *<100>!#	0.48 (0.01) *<100>!#	0.46 (0.01) *<100>!#	0.74 (0.02) *<100>!#	0.74 (0.01) *<100>!#	0.74 (0.01) *<100>!#	0.74 (0.01) *<100>!#	0.74 (0.01) *<100>!#	0.86 (0.01) *<100>!#	0.86 (0.01) *<100>!#	0.88 (0.01) *<100>!#	0.88 (0) *<100>!#	0.88 (0.01) *<100>!#
man_mi6_bias	-0.01 (0.04) *<7>	-0.01 (0.02) *<21>	-0.04 (0.01) *<98>	-0.03 (0.01) *<80>	-0.01 (0.01) *<12>	-0.14 (0.05) *<100>!#	-0.14 (0.03) *<100>!#	-0.17 (0.01) *<100>!#	-0.18 (0.01) *<100>!#	-0.15 (0.01) *<100>!#	-0.49 (0.03) *<100>!#	-0.49 (0.02) *<100>!#	-0.49 (0.01) *<100>!#	-0.5 (0.01) *<100>!#	-0.49 (0.01) *<100>!#	-0.76 (0.02) *<100>!#	-0.75 (0.01) *<100>!#	-0.75 (0.01) *<100>!#	-0.76 (0.01) *<100>!#	-0.76 (0.01) *<100>!#	-0.87 (0.01) *<100>!#	-0.87 (0.01) *<100>!#	-0.88 (0.01) *<100>!#	-0.89 (0) *<100>!#	-0.88 (0.01) *<100>!#
man_mi7	0.01 (0.02) *<34>	0.05 (0.02) *<97>	0.12 (0.01) *<100>!#	0.07 (0.01) *<100>!#	0.02 (0.01) *<44>	0.16 (0.03) *<100>!#	0.18 (0.02) *<100>!#	0.22 (0.01) *<100>!#	0.2 (0.01) *<100>!#	0.15 (0.01) *<100>!#	0.48 (0.02) *<100>!#	0.48 (0.02) *<100>!#	0.49 (0.01) *<100>!#	0.49 (0.01) *<100>!#	0.47 (0.01) *<100>!#	0.75 (0.02) *<100>!#	0.74 (0.01) *<100>!#	0.75 (0.01) *<100>!#	0.75 (0.01) *<100>!#	0.74 (0.01) *<100>!#	0.86 (0.01) *<100>!#	0.87 (0.01) *<100>!#	0.88 (0) *<100>!#	0.88 (0) *<100>!#	0.88 (0.01) *<100>!#
man_bias1	-0.04 (0.01) *<100>	-0.09 (0.01) *<100>!#	-0.3 (0.01) *<100>!#	-0.19 (0.01) *<100>!#	-0.05 (0.01) *<100>	-0.29 (0.02) *<100>!#	-0.34 (0.02) *<100>!#	-0.44 (0.01) *<100>!#	-0.41 (0.01) *<100>!#	-0.3 (0.01) *<100>!#	-0.65 (0.01) *<100>!#	-0.66 (0.01) *<100>!#	-0.69 (0.01) *<100>!#	-0.69 (0.01) *<100>!#	-0.67 (0.01) *<100>!#	-0.85 (0.01) *<100>!#	-0.85 (0.01) *<100>!#	-0.85 (0) *<100>!#	-0.86 (0) *<100>!#	-0.85 (0) *<100>!#	-0.92 (0.01) *<100>!#	-0.92 (0) *<100>!#	-0.93 (0) *<100>!#	-0.93 (0) *<100>!#	-0.93 (0) *<100>!#
man_bias2	-0.04 (0.01) *<100>	-0.11 (0.01) *<100>!#	-0.29 (0.01) *<100>!#	-0.16 (0.01) *<100>!#	-0.03 (0.01) *<95>	-0.29 (0.02) *<100>!#	-0.33 (0.01) *<100>!#	-0.42 (0.01) *<100>!#	-0.39 (0.01) *<100>!#	-0.29 (0.01) *<100>!#	-0.64 (0.01) *<100>!#	-0.65 (0.01) *<100>!#	-0.67 (0.01) *<100>!#	-0.68 (0.01) *<100>!#	-0.66 (0.01) *<100>!#	-0.84 (0.01) *<100>!#	-0.84 (0.01) *<100>!#	-0.85 (0) *<100>!#	-0.85 (0) *<100>!#	-0.85 (0) *<100>!#	-0.92 (0.01) *<100>!#	-0.92 (0) *<100>!#	-0.93 (0) *<100>!#	-0.93 (0) *<100>!#	-0.93 (0) *<100>!#
man_bias	0.02 (0.01) *<49>	0.04 (0.01) *<99>	0.15 (0.01) *<100>!#	0.14 (0.01) *<100>!#	0.04 (0.01) *<99>	0.16 (0.02) *<100>!#	0.18 (0.01) *<100>!#	0.24 (0.01) *<100>!#	0.25 (0.01) *<100>!#	0.19 (0.01) *<100>!#	0.5 (0.02) *<100>!#	0.51 (0.01) *<100>!#	0.53 (0.01) *<100>!#	0.53 (0.01) *<100>!#	0.51 (0.01) *<100>!#	0.77 (0.01) *<100>!#	0.76 (0.01) *<100>!#	0.77 (0.01) *<100>!#	0.77 (0.01) *<100>!#	0.77 (0.01) *<100>!#	0.87 (0.01) *<100>!#	0.88 (0.01) *<100>!#	0.89 (0) *<100>!#	0.89 (0) *<100>!#	0.89 (0) *<100>!#

**Table 18:** Odds ratios between manic episodes and the input symptoms of major depressive episodes by assumed symptom prevalence and correlations NA = not applicable due to 0 case in the denominators for the calculation of odds ratios Mean (standard deviation) *<number> = the numbers of significant statistics (p < 0.05) in 100 simulations !# = p < 0.001 in all of the 100 simulations !@ = p < 0.001 in some of the 100 simulations mde = Major Depressive Episodes; mde_ma1 = Depressed mood for more than two weeks.; mde_ma2 = Loss of interest or pleasure in daily activities for more than two weeks.; mde_mi3 = Significant unintentional weight loss or gain; mde_mi3_1 = Significant unintentional weight gain; mde_mi3_2 = Significant unintentional weight loss; mde_mi3_bias = Information of the domain not explained by the input variables; mde_mi4 = Insomnia or sleeping too much; mde_mi4_1 = Insomnia; mde_mi4_2 = Sleeping too much; mde_mi4_bias = Information of the domain not explained by the input variables; mde_mi5 = Agitation or psychomotor retardation noticed by others; mde_mi5_1 = Agitation; mde_mi5_2 = Psychomotor retardation noticed by others; mde_mi5_bias = Information of the domain not explained by the input variables; mde_mi6 = Fatigue or loss of energy; mde_mi6_1 = Fatigue; mde_mi6_2 = Loss of energy; mde_mi6_bias = Information of the domain not explained by the input variables; mde_mi7 = Feelings of worthlessness or excessive guilt; mde_mi7_1 = Feelings of worthlessness; mde_mi7_2 = Feelings of excessive guilt; mde_mi7_bias = Information of the domain not explained by the input variables; mde_mi8 = Diminished ability to think or concentrate, or indecisiveness+; mde_mi8_1 = Diminished ability to think or concentrate; mde_mi8_2 = Indecisiveness; mde_mi8_bias = Information of the domain not explained by the input variables; mde_mi9 = Recurrent thoughts of death; mde_bias1 = Information due to top censoring by choosing three domains in minor criteria; mde_bias2 = Information due to top censoring by choosing four domains in minor criteria; mde_bias = Information of diagnosis not explained by the domain

Assumed symptom prevalence	0.05	0.1	0.3	0.5	0.7	0.05	0.1	0.3	0.5	0.7	0.05	0.1	0.3	0.5	0.7	0.05	0.1	0.3	0.5	0.7	0.05	0.1	0.3	0.5	0.7
Assumed symptom correlations	0	0	0	0	0	0.1	0.1	0.1	0.1	0.1	0.4	0.4	0.4	0.4	0.4	0.7	0.7	0.7	0.7	0.7	0.9	0.9	0.9	0.9	0.9
mde	0 (0)	0.38 (3.77) *<1>	1.01 (0.13) *<9>	1 (0.04) *<4>	1.01 (0.05) *<8>	0.78 (1.9) *<3>	0.96 (0.67) *<2>	1 (0.09) *<6>	1.01 (0.05) *<7>	1 (0.04) *<5>	1.02 (0.42) *<4>	1.01 (0.23) *<10>	1.01 (0.06) *<4>	1 (0.05) *<5>	1 (0.04) *<6>	0.99 (0.27) *<4>	0.98 (0.12) *<3>	0.99 (0.05) *<7>	1 (0.04) *<5>	1 (0.04) *<4>	0.97 (0.24) *<5>	1.02 (0.13) *<7>	1 (0.05) *<3>	1 (0.04)	1.01 (0.04) *<4>
mde_ma1	NA	1 (0.76) *<2>	1.01 (0.07) *<7>	1 (0.03) *<3>	1 (0.05) *<4>	0.93 (0.58) *<7>	1.02 (0.23) *<5>	1 (0.06) *<4>	1 (0.04) *<5>	0.99 (0.05) *<4>	1.05 (0.27) *<3>	1.02 (0.15) *<7>	1 (0.05) *<3>	1 (0.04) *<5>	1 (0.05) *<7>	0.98 (0.21) *<5>	0.98 (0.11) *<5>	1 (0.05) *<4>	1 (0.04) *<7>	1 (0.04) *<3>	0.97 (0.22) *<4>	1.01 (0.12) *<8>	1.01 (0.05) *<2>	1 (0.04) *<1>	1 (0.05) *<3>
mde_ma2	NA	1.06 (0.77) *<6>	1 (0.06) *<3>	1 (0.04) *<5>	1.01 (0.05) *<8>	1.02 (0.51) *<3>	0.97 (0.21) *<4>	1 (0.05) *<3>	1.01 (0.04) *<6>	1 (0.05) *<7>	0.99 (0.27) *<2>	1.01 (0.14) *<5>	1.01 (0.05) *<4>	1 (0.04) *<7>	1 (0.05) *<6>	1.01 (0.24) *<5>	0.99 (0.11) *<5>	1 (0.05) *<5>	1 (0.04) *<4>	1 (0.04) *<4>	0.98 (0.21) *<2>	1.02 (0.12) *<5>	1 (0.04) *<1>	1 (0.04) *<2>	1 (0.04) *<3>
mde_mi3	NA	0.99 (0.6) *<7>	1 (0.06) *<6>	1 (0.05) *<9>	1 (0.07) *<5>	1 (0.43) *<3>	1.01 (0.17) *<4>	1.01 (0.05) *<2>	1 (0.05) *<7>	1 (0.07) *<6>	1.07 (0.24) *<5>	1.02 (0.11) *<3>	1 (0.05) *<4>	1 (0.05) *<8>	1.01 (0.06) *<8>	1.01 (0.19) *<2>	0.99 (0.11) *<5>	1 (0.05) *<3>	1 (0.04) *<7>	1 (0.05) *<2>	0.97 (0.18) *<3>	1.01 (0.11) *<7>	1 (0.05) *<3>	1 (0.03) *<2>	1.01 (0.05) *<3>
mde_mi3_1	NA	0.91 (0.69) *<4>	1 (0.07) *<6>	1 (0.04) *<5>	1 (0.04) *<4>	1.08 (0.57) *<6>	1.06 (0.22) *<5>	1.01 (0.06) *<7>	1 (0.04) *<3>	1 (0.05) *<6>	1.07 (0.28) *<5>	1.02 (0.15) *<5>	1 (0.05) *<3>	1 (0.04) *<7>	1 (0.05) *<5>	1 (0.22) *<4>	0.99 (0.12) *<6>	1 (0.05) *<3>	1 (0.04) *<6>	1 (0.05) *<6>	0.96 (0.2) *<3>	1.02 (0.11) *<6>	1 (0.05) *<2>	1 (0.04) *<3>	1 (0.05) *<2>
mde_mi3_2	NA	1.04 (0.81) *<4>	0.99 (0.07) *<6>	1 (0.04) *<7>	1 (0.04) *<4>	0.96 (0.55) *<3>	0.96 (0.24) *<7>	1.01 (0.05) *<3>	1 (0.04) *<7>	1.01 (0.05) *<6>	1.05 (0.28) *<3>	1.02 (0.14) *<3>	1 (0.06) *<6>	1 (0.04) *<5>	1 (0.05) *<7>	1.01 (0.23) *<4>	0.99 (0.12) *<7>	1 (0.05) *<4>	1.01 (0.04) *<4>	1 (0.05) *<3>	0.97 (0.18) *<2>	1.02 (0.11) *<5>	1 (0.04) *<1>	1 (0.03) *<2>	1.01 (0.05) *<6>
mde_mi3_bias	NA	NA	1.02 (0.11) *<5>	1 (0.05) *<8>	1 (0.04) *<6>	NA	NA	1 (0.09) *<7>	1 (0.04) *<5>	1 (0.04) *<5>	1.18 (0.75) *<7>	1.02 (0.2) *<2>	1 (0.07) *<13>	1 (0.05) *<4>	1 (0.04) *<5>	1.09 (0.36) *<6>	1.04 (0.16) *<5>	1 (0.06) *<4>	0.99 (0.04) *<5>	1.01 (0.05) *<5>	1.09 (0.24) *<1>	1 (0.12) *<5>	1 (0.05) *<3>	1 (0.04) *<1>	1 (0.04) *<4>
mde_mi4	0.33 (1.48)	1.04 (0.62) *<4>	1.01 (0.05) *<4>	1 (0.05) *<3>	1 (0.08) *<4>	1.04 (0.42) *<5>	1.01 (0.17) *<1>	1 (0.05) *<3>	1 (0.05) *<8>	1 (0.07) *<7>	1.02 (0.21) *<4>	1.01 (0.11) *<3>	1 (0.05) *<6>	0.99 (0.04) *<3>	1.01 (0.06) *<5>	1 (0.21) *<6>	0.99 (0.11) *<6>	1 (0.05) *<3>	1.01 (0.04) *<4>	0.99 (0.05) *<1>	0.97 (0.19) *<4>	1.01 (0.11) *<7>	1 (0.05) *<2>	1 (0.04) *<1>	1.01 (0.05) *<7>
mde_mi4_1	0.69 (3.13) *<2>	1.01 (0.78) *<2>	1.01 (0.06) *<3>	1 (0.04) *<4>	1 (0.05) *<6>	1.02 (0.5) *<4>	1.01 (0.23) *<7>	1.01 (0.06) *<4>	1 (0.04) *<4>	1 (0.05) *<6>	1.01 (0.29) *<8>	1.02 (0.13) *<4>	1.01 (0.06) *<9>	0.99 (0.05) *<9>	1 (0.04) *<4>	1.01 (0.25) *<9>	0.98 (0.12) *<6>	1 (0.05) *<3>	1.01 (0.04) *<5>	0.99 (0.04) *<2>	0.97 (0.21) *<4>	1.01 (0.13) *<9>	1 (0.05) *<1>	1 (0.03) *<1>	1.01 (0.05) *<5>
mde_mi4_2	0 (0)	1.03 (0.67) *<3>	1 (0.06) *<2>	1 (0.04) *<7>	1 (0.04) *<6>	1.03 (0.59) *<4>	1.03 (0.22) *<3>	1 (0.06) *<7>	1 (0.04) *<6>	0.99 (0.04) *<2>	1.03 (0.27) *<5>	1.02 (0.15) *<4>	1 (0.06) *<7>	1 (0.05) *<9>	1.01 (0.05) *<5>	0.99 (0.24) *<4>	0.99 (0.11) *<3>	1 (0.05) *<4>	1 (0.04) *<2>	0.99 (0.04) *<6>	0.97 (0.2) *<1>	1.01 (0.11) *<5>	1 (0.05) *<1>	1 (0.04) *<2>	1.01 (0.05) *<7>
mde_mi4_bias	NA	NA	1.01 (0.11) *<6>	1 (0.04) *<2>	1 (0.04) *<4>	NA	1.35 (1.03) *<5>	1 (0.09) *<7>	1.01 (0.04) *<4>	1.01 (0.04) *<6>	NA	1 (0.21) *<7>	0.99 (0.08) *<11>	1 (0.05) *<9>	1 (0.04) *<6>	1.08 (0.33) *<7>	1.03 (0.14) *<4>	1 (0.05) *<4>	1 (0.04) *<3>	1.01 (0.04) *<4>	1.09 (0.26) *<3>	1.01 (0.13) *<6>	1 (0.04) *<2>	1 (0.03) *<3>	1 (0.05) *<6>
mde_mi5	NA	1.26 (0.65) *<7>	1 (0.06) *<8>	1 (0.05) *<5>	1 (0.09) *<6>	1.08 (0.44) *<6>	1.05 (0.21) *<10>	1 (0.06) *<5>	1 (0.04) *<3>	1.01 (0.06) *<4>	1.01 (0.22) *<7>	1 (0.11) *<4>	1 (0.05) *<4>	1 (0.05) *<5>	1.01 (0.07) *<10>	1 (0.2) *<3>	0.99 (0.11) *<5>	1 (0.04) *<4>	1 (0.04) *<9>	0.99 (0.05) *<7>	0.97 (0.19) *<2>	1 (0.11) *<2>	1 (0.05) *<3>	1 (0.04) *<4>	1.01 (0.05) *<4>
mde_mi5_1	NA	1.24 (0.8) *<5>	1 (0.07) *<8>	1 (0.04) *<3>	1 (0.05) *<7>	1.07 (0.59) *<4>	1.03 (0.25) *<6>	0.99 (0.06) *<6>	1 (0.04) *<4>	1 (0.04) *<1>	0.98 (0.29) *<6>	0.99 (0.12) *<3>	1 (0.05) *<8>	1 (0.04) *<9>	1 (0.05) *<6>	0.99 (0.22) *<3>	0.99 (0.12) *<4>	1 (0.05) *<4>	1 (0.04) *<8>	0.99 (0.05) *<3>	0.97 (0.2) *<1>	1.01 (0.12) *<4>	1 (0.05) *<4>	1 (0.04) *<2>	1.01 (0.05) *<4>
mde_mi5_2	0 (0)	1.21 (0.8) *<4>	1.01 (0.07) *<3>	1 (0.04) *<4>	1 (0.06) *<10>	1.1 (0.59) *<4>	1.04 (0.24) *<7>	1.01 (0.06) *<5>	1 (0.04) *<3>	1 (0.05) *<6>	1.01 (0.3) *<6>	1.02 (0.14) *<9>	1.01 (0.05) *<4>	1 (0.05) *<9>	1 (0.05) *<7>	1.03 (0.23) *<2>	1 (0.11) *<3>	1 (0.05) *<8>	1 (0.04) *<6>	0.99 (0.05) *<2>	0.96 (0.21) *<4>	1.01 (0.12) *<4>	1 (0.05) *<5>	1 (0.04) *<3>	1 (0.05) *<6>
mde_mi5_bias	NA	NA	0.99 (0.1) *<4>	1 (0.05) *<6>	1 (0.05) *<2>	NA	NA	1 (0.08) *<4>	1 (0.04) *<5>	1 (0.04) *<2>	NA	1.02 (0.21) *<5>	1 (0.07) *<6>	1 (0.05) *<8>	1 (0.05) *<9>	1.07 (0.32) *<4>	1.02 (0.14) *<5>	1.01 (0.05) *<5>	1 (0.04) *<6>	1.01 (0.05) *<5>	1.1 (0.27) *<4>	1 (0.13) *<8>	1 (0.05) *<1>	1 (0.03) *<1>	1 (0.05) *<4>
mde_mi6	NA	1.05 (0.51) *<1>	1 (0.06) *<9>	1.01 (0.04) *<2>	1.01 (0.08) *<5>	1.02 (0.43) *<6>	1.02 (0.17) *<2>	1 (0.05) *<2>	1 (0.05) *<1>	1 (0.07) *<5>	0.98 (0.21) *<2>	1.01 (0.12) *<8>	1 (0.05) *<5>	1 (0.04) *<7>	1 (0.06) *<6>	0.99 (0.19) *<1>	0.99 (0.1)	1 (0.04) *<3>	1 (0.04) *<6>	0.99 (0.04) *<4>	0.97 (0.19) *<3>	1.01 (0.11) *<7>	1 (0.05) *<4>	1 (0.03) *<4>	1 (0.04) *<2>
mde_mi6_1	NA	1.09 (0.73) *<4>	1 (0.06) *<6>	1 (0.04) *<6>	1 (0.05) *<8>	1.08 (0.54) *<1>	1.01 (0.22) *<4>	1 (0.06) *<4>	1 (0.04) *<6>	1 (0.04) *<1>	1.01 (0.26) *<2>	1.01 (0.14) *<6>	1 (0.05) *<3>	1.01 (0.04) *<7>	1 (0.04) *<2>	1 (0.24) *<5>	0.99 (0.12) *<5>	0.99 (0.04) *<3>	1 (0.04) *<6>	0.99 (0.04) *<6>	0.97 (0.21) *<2>	1.01 (0.12) *<5>	1 (0.05) *<2>	1 (0.04) *<5>	1.01 (0.05) *<4>
mde_mi6_2	NA	1.05 (0.73) *<1>	1 (0.07) *<6>	1.01 (0.04) *<4>	1.01 (0.05) *<5>	0.94 (0.57) *<3>	1.03 (0.21) *<2>	0.99 (0.06) *<3>	1 (0.04) *<3>	1 (0.05) *<5>	0.97 (0.26) *<3>	1.01 (0.16) *<9>	1 (0.05) *<3>	1 (0.04) *<10>	1.01 (0.05) *<4>	0.98 (0.22) *<5>	0.99 (0.11) *<2>	1 (0.05) *<3>	1.01 (0.04) *<7>	0.99 (0.05) *<6>	0.97 (0.2) *<2>	1.01 (0.12) *<6>	1 (0.05) *<3>	1 (0.03) *<1>	1 (0.04) *<2>
mde_mi6_bias	NA	NA	1.01 (0.12) *<11>	1 (0.05) *<11>	1 (0.04) *<4>	NA	1.35 (0.99) *<1>	1.01 (0.09) *<6>	1 (0.05) *<8>	1 (0.04) *<3>	1.18 (0.55) *<1>	1.05 (0.23) *<7>	1 (0.06) *<2>	0.99 (0.04) *<9>	1 (0.04) *<3>	1.11 (0.38) *<9>	1.04 (0.16) *<7>	1.01 (0.05) *<4>	1 (0.04) *<4>	1.01 (0.05) *<7>	1.09 (0.26) *<4>	1 (0.14) *<7>	1 (0.05) *<3>	1 (0.03) *<1>	1 (0.05) *<4>
mde_mi7	NA	1.09 (0.64) *<5>	1 (0.05) *<3>	0.99 (0.04) *<1>	1 (0.08) *<5>	1.01 (0.39) *<2>	1.01 (0.18) *<7>	1 (0.05) *<6>	1 (0.04) *<4>	1 (0.06) *<5>	1.03 (0.2) *<4>	1.03 (0.13) *<9>	1.01 (0.05) *<1>	1 (0.05) *<4>	1 (0.05) *<4>	1.02 (0.2) *<4>	1 (0.11) *<6>	1 (0.04) *<4>	1 (0.04) *<6>	0.99 (0.04) *<3>	0.97 (0.2) *<3>	1.01 (0.11) *<9>	1 (0.05) *<2>	1 (0.04) *<2>	1.01 (0.05) *<4>
mde_mi7_1	NA	1.06 (0.7) *<4>	1.01 (0.06) *<5>	1 (0.04) *<3>	1 (0.05) *<7>	0.95 (0.54) *<6>	1.02 (0.21) *<3>	0.99 (0.06) *<8>	1 (0.04) *<4>	1 (0.05) *<6>	1.03 (0.26) *<3>	1.02 (0.13) *<5>	1.01 (0.05) *<2>	1 (0.04) *<7>	1 (0.05) *<6>	1.02 (0.24) *<2>	1 (0.13) *<8>	1 (0.05) *<2>	1 (0.04) *<4>	0.99 (0.04) *<4>	0.97 (0.2) *<3>	1.01 (0.11) *<6>	1 (0.05) *<5>	1 (0.03) *<2>	1 (0.04) *<2>
mde_mi7_2	NA	1.11 (0.84) *<5>	1 (0.06) *<7>	1 (0.04) *<7>	1 (0.05) *<4>	1.04 (0.57) *<3>	0.99 (0.21) *<5>	1.01 (0.06) *<4>	1 (0.04) *<3>	0.99 (0.04) *<4>	1.01 (0.26) *<2>	1.02 (0.15) *<6>	1 (0.06) *<8>	1 (0.04) *<5>	1 (0.05) *<5>	1.01 (0.24) *<5>	0.98 (0.12) *<8>	1 (0.04) *<4>	1 (0.04) *<6>	0.99 (0.04) *<6>	0.98 (0.22) *<6>	1.01 (0.12) *<6>	1.01 (0.05) *<2>	1 (0.03) *<1>	1.01 (0.05) *<3>
mde_mi7_bias	NA	NA	1 (0.11) *<5>	1 (0.04) *<4>	1 (0.05) *<5>	NA	NA	1.01 (0.09) *<6>	1 (0.04) *<4>	1 (0.04) *<5>	1.22 (0.68) *<5>	1.02 (0.19) *<1>	1 (0.06) *<3>	1 (0.04) *<5>	1 (0.05) *<8>	1.07 (0.32) *<4>	1.04 (0.15) *<6>	1.01 (0.05) *<2>	1 (0.04) *<4>	1 (0.05) *<5>	1.09 (0.28) *<5>	1 (0.12) *<7>	1 (0.05) *<5>	1 (0.03) *<2>	1 (0.04) *<5>
mde_mi8	NA	1.02 (0.59) *<4>	0.99 (0.06) *<5>	1 (0.05) *<3>	1.02 (0.08) *<5>	1 (0.41) *<5>	1.03 (0.17) *<2>	1 (0.05) *<3>	1.01 (0.04) *<4>	0.99 (0.07) *<5>	1.03 (0.2) *<3>	1.01 (0.13) *<7>	1.01 (0.05) *<6>	0.99 (0.04) *<7>	1 (0.05) *<3>	0.98 (0.2) *<4>	0.99 (0.12) *<6>	1 (0.04) *<3>	1 (0.04) *<8>	0.99 (0.05) *<5>	0.97 (0.19) *<5>	1.01 (0.11) *<4>	1 (0.05) *<4>	1 (0.04) *<2>	1.01 (0.05) *<5>
mde_mi8_1	NA	1.03 (0.84) *<7>	0.99 (0.06) *<4>	1 (0.04) *<8>	1.01 (0.05) *<6>	1 (0.58) *<7>	1 (0.21) *<5>	1 (0.06) *<4>	1 (0.04) *<5>	1 (0.05) *<4>	1 (0.26) *<1>	1.01 (0.14) *<8>	1.01 (0.06) *<7>	1 (0.04) *<7>	1 (0.04) *<2>	1 (0.25) *<6>	0.99 (0.12) *<6>	1 (0.05) *<4>	1.01 (0.04) *<6>	0.99 (0.05) *<6>	0.98 (0.22) *<2>	1.02 (0.11) *<6>	1 (0.05) *<5>	1 (0.04) *<2>	1.01 (0.05) *<5>
mde_mi8_2	NA	1.03 (0.72) *<2>	1 (0.06) *<6>	1 (0.04) *<5>	1 (0.05) *<2>	1.02 (0.58) *<6>	1.03 (0.2) *<5>	1.01 (0.06) *<4>	1.01 (0.04) *<5>	1 (0.05) *<7>	1.04 (0.27) *<7>	1.01 (0.15) *<6>	1 (0.06) *<9>	1 (0.04) *<2>	1 (0.05) *<3>	0.99 (0.22) *<5>	1 (0.13) *<7>	1 (0.05) *<4>	1 (0.04) *<8>	0.99 (0.05) *<5>	0.97 (0.21) *<3>	1.01 (0.11) *<3>	1 (0.05) *<2>	1 (0.04) *<2>	1 (0.05) *<8>
mde_mi8_bias	NA	NA	1.02 (0.09) *<2>	1 (0.04) *<3>	1 (0.04) *<6>	NA	NA	1 (0.08) *<3>	0.99 (0.04) *<1>	1 (0.04) *<7>	1.23 (0.59) *<2>	1.06 (0.22) *<3>	1 (0.07) *<4>	1 (0.04) *<6>	1 (0.04) *<6>	1.08 (0.36) *<5>	1.02 (0.14) *<5>	1 (0.05) *<4>	1 (0.04) *<5>	1.01 (0.05) *<8>	1.08 (0.26) *<5>	0.99 (0.12) *<4>	1 (0.05) *<2>	1 (0.03) *<2>	1 (0.05) *<6>
mde_mi9	NA	1.05 (0.73) *<4>	1 (0.07) *<5>	1 (0.04) *<2>	1 (0.05) *<6>	0.98 (0.57) *<4>	1.05 (0.24) *<6>	1.01 (0.06) *<3>	1 (0.04) *<4>	1 (0.04) *<5>	1.03 (0.28) *<4>	1.01 (0.14) *<6>	1 (0.05) *<5>	1.01 (0.04) *<9>	1 (0.04) *<3>	1.01 (0.25) *<4>	0.99 (0.13) *<7>	1 (0.05) *<6>	1 (0.04) *<5>	1 (0.05) *<5>	0.98 (0.21) *<3>	1.01 (0.11) *<6>	1 (0.05) *<1>	1 (0.04) *<3>	1 (0.05) *<5>
mde_bias1	NA	NA	NA	NA	NA	NA	NA	NA	NA	NA	NA	NA	NA	NA	NA	NA	NA	NA	NA	NA	NA	NA	NA	NA	NA
mde_bias2	NA	NA	NA	NA	NA	NA	NA	NA	NA	NA	NA	NA	NA	NA	NA	NA	NA	NA	NA	NA	NA	NA	NA	NA	NA
mde_bias	NA	NA	NA	NA	NA	NA	NA	NA	NA	NA	NA	NA	NA	NA	NA	NA	NA	NA	NA	NA	NA	NA	NA	NA	NA
